# Single-cell multi-omics analysis of the immune response in COVID-19

**DOI:** 10.1038/s41591-021-01329-2

**Published:** 2021-04-20

**Authors:** Emily Stephenson, Gary Reynolds, Rachel A. Botting, Fernando J. Calero-Nieto, Michael D. Morgan, Zewen Kelvin Tuong, Karsten Bach, Waradon Sungnak, Kaylee B. Worlock, Masahiro Yoshida, Natsuhiko Kumasaka, Katarzyna Kania, Justin Engelbert, Bayanne Olabi, Jarmila Stremenova Spegarova, Nicola K. Wilson, Nicole Mende, Laura Jardine, Louis C. S. Gardner, Issac Goh, Dave Horsfall, Jim McGrath, Simone Webb, Michael W. Mather, Rik G. H. Lindeboom, Emma Dann, Ni Huang, Krzysztof Polanski, Elena Prigmore, Florian Gothe, Jonathan Scott, Rebecca P. Payne, Kenneth F. Baker, Aidan T. Hanrath, Ina C. D. Schim van der Loeff, Andrew S. Barr, Amada Sanchez-Gonzalez, Laura Bergamaschi, Federica Mescia, Josephine L. Barnes, Eliz Kilich, Angus de Wilton, Anita Saigal, Aarash Saleh, Sam M. Janes, Claire M. Smith, Nusayhah Gopee, Caroline Wilson, Paul Coupland, Jonathan M. Coxhead, Vladimir Yu Kiselev, Stijn van Dongen, Jaume Bacardit, Hamish W. King, Cambridge Institute of Therapeutic Immunology and Infectious Disease-National Institute of Health Research (CITIID-NIHR) COVID-19 BioResource Collaboration, Stephen Baker, John R. Bradley, Gordon Dougan, Ian G. Goodfellow, Ravindra K. Gupta, Christoph Hess, Nathalie Kingston, Paul J. Lehner, Nicholas J. Matheson, Willem H. Owehand, Caroline Saunders, Kenneth G. C. Smith, Charlotte Summers, James E. D. Thaventhiran, Mark Toshner, Michael P. Weekes, Ashlea Bucke, Jo Calder, Laura Canna, Jason Domingo, Anne Elmer, Stewart Fuller, Julie Harris, Sarah Hewitt, Jane Kennet, Sherly Jose, Jenny Kourampa, Anne Meadows, Criona O’Brien, Jane Price, Cherry Publico, Rebecca Rastall, Carla Ribeiro, Jane Rowlands, Valentina Ruffolo, Hugo Tordesillas, Ben Bullman, Benjamin J. Dunmore, Stuart Fawke, Stefan Gräf, Josh Hodgson, Christopher Huang, Kelvin Hunter, Emma Jones, Ekaterina Legchenko, Cecilia Matara, Jennifer Martin, Ciara O’Donnell, Linda Pointon, Nicole Pond, Joy Shih, Rachel Sutcliffe, Tobias Tilly, Carmen Treacy, Zhen Tong, Jennifer Wood, Marta Wylot, Ariana Betancourt, Georgie Bower, Aloka De Sa, Madeline Epping, Oisin Huhn, Sarah Jackson, Isobel Jarvis, Jimmy Marsden, Francesca Nice, Georgina Okecha, Ommar Omarjee, Marianne Perera, Nathan Richoz, Rahul Sharma, Lori Turner, Eckart M. D. D. De Bie, Katherine Bunclark, Masa Josipovic, Michael Mackay, Alice Michael, Sabrina Rossi, Mayurun Selvan, Sarah Spencer, Cissy Yong, Ali Ansaripour, Lucy Mwaura, Caroline Patterson, Gary Polwarth, Petra Polgarova, Giovanni di Stefano, John Allison, Helen Butcher, Daniela Caputo, Debbie Clapham-Riley, Eleanor Dewhurst, Anita Furlong, Barbara Graves, Jennifer Gray, Tasmin Ivers, Mary Kasanicki, Emma Le Gresley, Rachel Linger, Sarah Meloy, Francesca Muldoon, Nigel Ovington, Sofia Papadia, Isabel Phelan, Hannah Stark, Kathleen E. Stirrups, Paul Townsend, Neil Walker, Jennifer Webster, Anthony J. Rostron, A. John Simpson, Sophie Hambleton, Elisa Laurenti, Paul A. Lyons, Kerstin B. Meyer, Marko Z. Nikolić, Christopher J. A. Duncan, Kenneth G. C. Smith, Sarah A. Teichmann, Menna R. Clatworthy, John C. Marioni, Berthold Göttgens, Muzlifah Haniffa

**Affiliations:** 1 Biosciences Institute, Newcastle University, Newcastle upon Tyne, UK; 2 Wellcome - MRC Cambridge Stem Cell Institute, University of Cambridge, Cambridge, UK; 3 European Molecular Biology Laboratory, European Bioinformatics Institute (EMBL-EBI), Wellcome Genome Campus, Cambridge, UK; 4 Cancer Research UK Cambridge Institute, University of Cambridge, Cambridge, UK; 5 Molecular Immunity Unit, Department of Medicine, University of Cambridge, Cambridge, UK; 6 Wellcome Sanger Institute, Wellcome Genome Campus, Cambridge, UK; 7 UCL Respiratory, Division of Medicine, University College London, London, UK; 8 Translational and Clinical Research Institute, Newcastle University, Newcastle upon Tyne, UK; 9 NIHR Newcastle Biomedical Research Centre, Newcastle Hospitals NHS Foundation Trust, Newcastle upon Tyne, UK; 10 Department of Infection and Tropical Medicine, Newcastle upon Tyne Hospitals NHS Foundation, Newcastle upon Tyne, UK; 11 Cambridge Institute of Therapeutic Immunology and Infectious Disease, Jeffrey Cheah Biomedical Centre, Cambridge Biomedical Campus, Cambridge, UK; 12 Department of Medicine, University of Cambridge, Cambridge Biomedical Campus, Cambridge, UK; 13 University College London Hospitals NHS Foundation Trust, London, UK; 14 Royal Free Hospital NHS Foundation Trust, London, UK; 15 UCL Great Ormond Street Institute of Child Health, London, UK; 16 Department of Dermatology, Newcastle Hospitals NHS Foundation Trust, Newcastle upon Tyne, UK; 17 The Innovation Lab Integrated COVID Hub North East, Newcastle Upon Tyne, UK; 18 School of Computing, Newcastle University, Newcastle Upon Tyne, UK; 19 Centre for Immunobiology, Blizard Institute, Queen Mary University of London, London, UK; 20 Integrated Critical Care Unit, Sunderland Royal Hospital, South Tyneside and Sunderland NHS Foundation Trust, Sunderland, UK; 21 Theory of Condensed Matter Group, Cavendish Laboratory/Department of Physics, University of Cambridge, Cambridge, UK; 22 Cambridge Institute for Therapeutic Immunology and Infectious Disease, Cambridge Biomedical Campus, Cambridge, UK; 23 NIHR Cambridge Biomedical Research Centre, Cambridge, UK; 24 Present Address: Department of Pediatrics, Dr. von Hauner Children’s Hospital, University Hospital, Ludwig-Maximilians-Universität Munich, Munich, Germany; 25 NIHR BioResource, Cambridge University Hospitals NHS Foundation, Cambridge Biomedical Campus, Cambridge, UK; 26 Division of Virology, Department of Pathology, University of Cambridge, Addenbrooke’s Hospital, Cambridge, UK; 27 Department of Biomedicine, University and University Hospital Basel, Basel, Switzerland; 28 Botnar Research Centre for Child Health (BRCCH), University Basel & ETH Zurich, Basel, Switzerland; 29 Department of Haematology, University of Cambridge, Cambridge Biomedical Campus, Cambridge, UK; 30 Cambridge Clinical Research Centre, NIHR Clinical Research Facility, Cambridge University Hospitals NHS Foundation Trust, Addenbrooke’s Hospital, Cambridge, UK; 31 Metabolic Research Laboratories, Wellcome Trust-Medical Research Council Institute of Metabolic Science, University of Cambridge, Cambridge, UK; 32 Royal Papworth Hospital NHS Foundation Trust, Cambridge Biomedical Campus, Cambridge, UK; 33 Addenbrooke’s Hospital, Cambridge, UK; 34 Department of Obstetrics & Gynaecology, The Rosie Maternity Hospital, Robinson Way, Cambridge, UK; 35 Department of Paediatrics, University of Cambridge, Cambridge Biomedical Campus, Cambridge, UK; 36 Cambridge Institute for Medical Research, University of Cambridge, Cambridge Biomedical Campus, Cambridge, UK; 37 Department of Veterinary Medicine, Cambridge, UK; 38 Department of Biochemistry, University of Cambridge, Cambridge, UK; 39 Cancer Molecular Diagnostics Laboratory, Department of Oncology, University of Cambridge, Cambridge, UK; 40 Department of Surgery, Addenbrooke’s Hospital, Cambridge, UK; 41 Department of Clinical Biochemistry and Immunology, Addenbrooke’s Hospital, Cambridge, UK; 42 Department of Public Health and Primary Care, School of Clinical Medicine, University of Cambridge, Cambridge Biomedical Campus, Cambridge, UK

**Keywords:** SARS-CoV-2, Haematopoiesis, Viral infection, RNA sequencing, Infection

## Abstract

Analysis of human blood immune cells provides insights into the coordinated response to viral infections such as severe acute respiratory syndrome coronavirus 2, which causes coronavirus disease 2019 (COVID-19). We performed single-cell transcriptome, surface proteome and T and B lymphocyte antigen receptor analyses of over 780,000 peripheral blood mononuclear cells from a cross-sectional cohort of 130 patients with varying severities of COVID-19. We identified expansion of nonclassical monocytes expressing complement transcripts (*CD16*^*+*^*C1QA/B/C*^*+*^) that sequester platelets and were predicted to replenish the alveolar macrophage pool in COVID-19. Early, uncommitted CD34^+^ hematopoietic stem/progenitor cells were primed toward megakaryopoiesis, accompanied by expanded megakaryocyte-committed progenitors and increased platelet activation. Clonally expanded CD8^+^ T cells and an increased ratio of CD8^+^ effector T cells to effector memory T cells characterized severe disease, while circulating follicular helper T cells accompanied mild disease. We observed a relative loss of IgA2 in symptomatic disease despite an overall expansion of plasmablasts and plasma cells. Our study highlights the coordinated immune response that contributes to COVID-19 pathogenesis and reveals discrete cellular components that can be targeted for therapy.

## Abstract (web summary)

Transcriptomic and proteomic profiling of blood samples from individuals with COVID-19 reveals immune cell and hematopoietic progenitor cell alterations that are differentially associated with disease severity.

## Main

The COVID-19 global pandemic has caused >120 million infections and 2.6 million deaths (as of 17 March 2021)^1,2^. Symptoms vary in severity and include acute respiratory distress syndrome, thrombosis and organ failure^3^. COVID-19 is caused by severe acute respiratory syndrome coronavirus 2 (SARS-CoV-2), a single-stranded RNA betacoronavirus that enters host cells through receptors such as angiotensin-converting enzyme 2 (ACE2) and neuropilin (NRP1), which are expressed widely, including in nasal epithelium^4–6^.

Several studies have highlighted a complex network of peripheral blood immune responses in COVID-19 infection^7,8^. A reduction in T cells with disease severity and reduced interferon (IFN)-γ production by lymphocytes have been reported^9^. However, an expansion of highly cytotoxic effector T cell subsets in moderate to severe disease^10,11^ and higher expression of exhaustion markers programmed cell death protein 1 and Tim-3 on CD8^+^ T cells have been described in patients receiving intensive care therapy^5^. In severe cases, classical monocytes have been shown to display a type 1 IFN inflammatory signature^6^; however, low levels of IFNα coupled with a reduction in plasmacytoid dendritic cells (DCs) have been reported in patients with critical disease^12^. Emergency and dysregulated myelopoiesis, and expanded activated megakaryocytes have also been reported^13–15^. Proliferating plasmablasts and extrafollicular B cell activation are present in critically ill patients, but despite high levels of SARS-CoV-2-specific antibodies and antibody-secreting cells, many of these patients do not recover^8,15^. To better understand the coordinated systemic immune response in individuals with asymptomatic and symptomatic COVID-19, we performed combined single-cell transcriptome, cell-surface protein and lymphocyte antigen receptor repertoire analysis of peripheral blood of a cross-sectional patient cohort and integrated results across three UK medical centers.

## Results

### Altered cellular profiles across COVID-19 severities

**Fig. 1:**
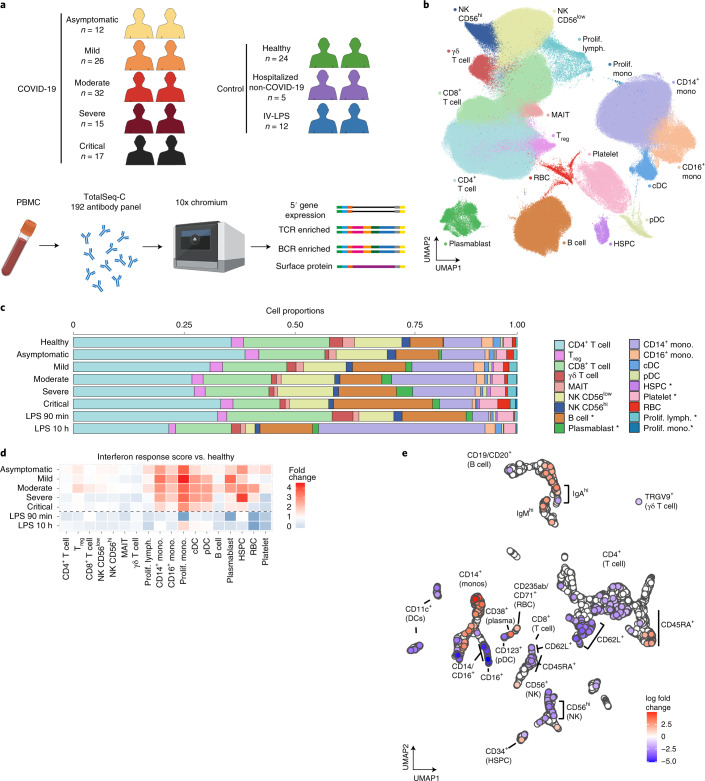
Single-cell multi-omics analysis of PBMCs from individuals with COVID-19 and controls. **a**, Overview of the participants included and the samples and data collected. Figure was created using BioRender.com. **b**, UMAP visualization of all 781,123 cells after QC. Leiden clusters based on 5′ gene expression shown and colored by cell type. Lymph, lymphocyte; mono, monocyte; prolif, proliferating. **c**, Bar plot of the proportion of cell types shown in **b**, separated by condition and COVID-19 severity status. Hypothesis testing was performed using quasi-likelihood *F*-test comparing healthy controls to individuals with COVID-19 for linear trends across disease severity groups (healthy > asymptomatic > mild > moderate > severe > critical). Differentially abundant cell types were determined using a 10% FDR and are marked with an asterisk. **d**, Enrichment of interferon response of each cell state separated by severity. IFN response was calculated using a published gene list (GO:0034340) **e**, UMAP computed using batch-corrected mean staining intensities of 188 antibodies for 4,241 hyperspheres. Each hypersphere represents an area in the 188-dimensional space and is colored by significant (spatial FDR < 0.05) severity-associated changes in abundance of cells within that space.

**Extended Data Fig. 1:**
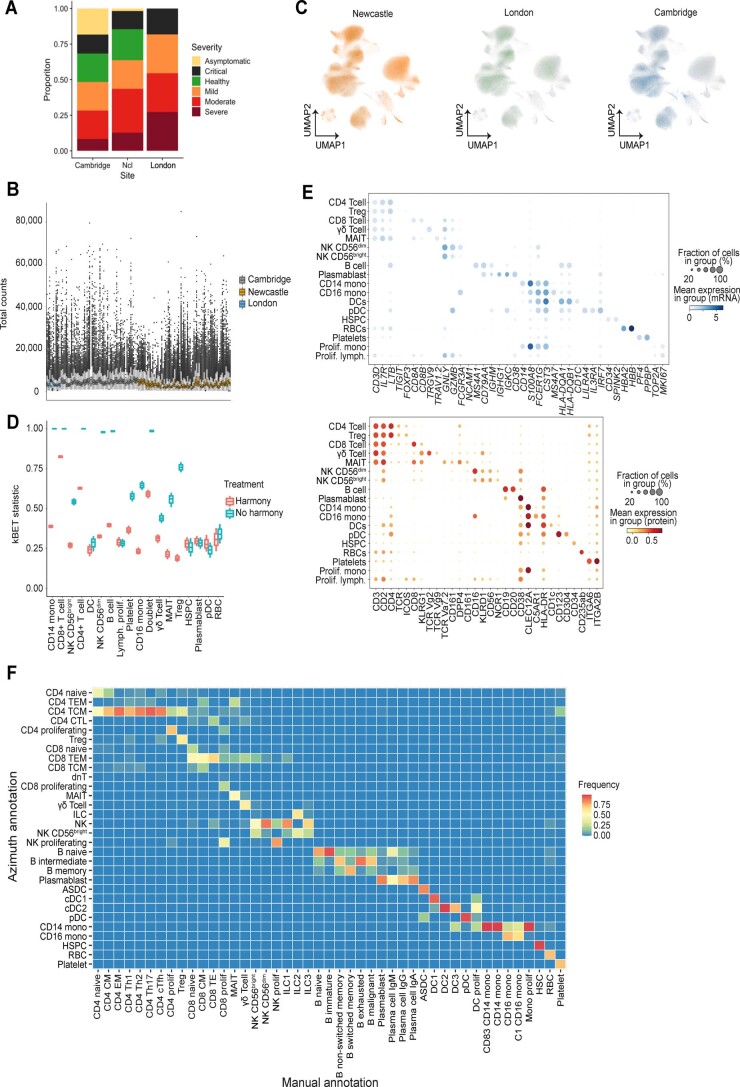
Single-cell analysis quality control and cell type definition for COVID PBMC single cell analysis. **a**, Bar chart showing the composition of sample severities across the three sites (n = 23 mild, n = 30 moderate, n = 11, severe, n = 10 critical biologically independent samples). **b**, Scatter plot displaying the total number of gene counts per sample from each site. **c**, UMAP visualisations from Fig. 1b coloured by site. **d**, Boxplot of kBET results calculated both before and after batch correction with Harmony for each cluster in Fig. 1b kBET statistic calculation using patient ID as the batch factor (n = 130 biologically independent samples, n = 627,172 cells in 1 experiment). Hinges indicate to 25th and 75th percentile and whiskers to lowest and highest value in 1.5*interquartile range. **e**, Dot plots of 5’ gene expression (top; blue) and surface protein (bottom; red) expression for populations shown in Fig. 1a where the colour is scaled by mean expression and the dot size is proportional to the percent of the population expressing the gene/protein, respectively. **f**, Tile plot showing percentage concordance between COVID-19 PBMC annotation (x-axis) and Azimuth annotation (y-axis) (https://satijalab.org/azimuth/).

We generated single-cell combined transcriptome and surface proteome data from peripheral blood mononuclear cells (PBMCs) from individuals with asymptomatic, mild, moderate, severe and critical^16^ COVID-19 across three UK centers in Newcastle, Cambridge and London (Fig. 1a, Extended Data Fig. 1a and Supplementary Table 1). Controls included healthy volunteers, individuals with non-COVID-19 severe respiratory illness and healthy volunteers administered with intravenous lipopolysaccharide (IV-LPS) as a surrogate for an acute systemic inflammatory response (Fig. 1a and Supplementary Table 2). We sequenced 1,141,860 cells from 143 samples. Following computational doublet removal, 781,123 cells passed quality control (QC; minimum of 200 genes and <10% mitochondrial reads per cell; Extended Data Fig. 1b). Data were integrated using Harmony^17^ with good mixing of cells by the kBET statistic calculated for each cluster across samples (rejection rate improved from 0.62 to 0.36 following integration, *P* < 2.1 × 10^−8^ by Wilcoxon paired signed-rank test; Extended Data Fig. 1c,d).

**Fig. 2:**
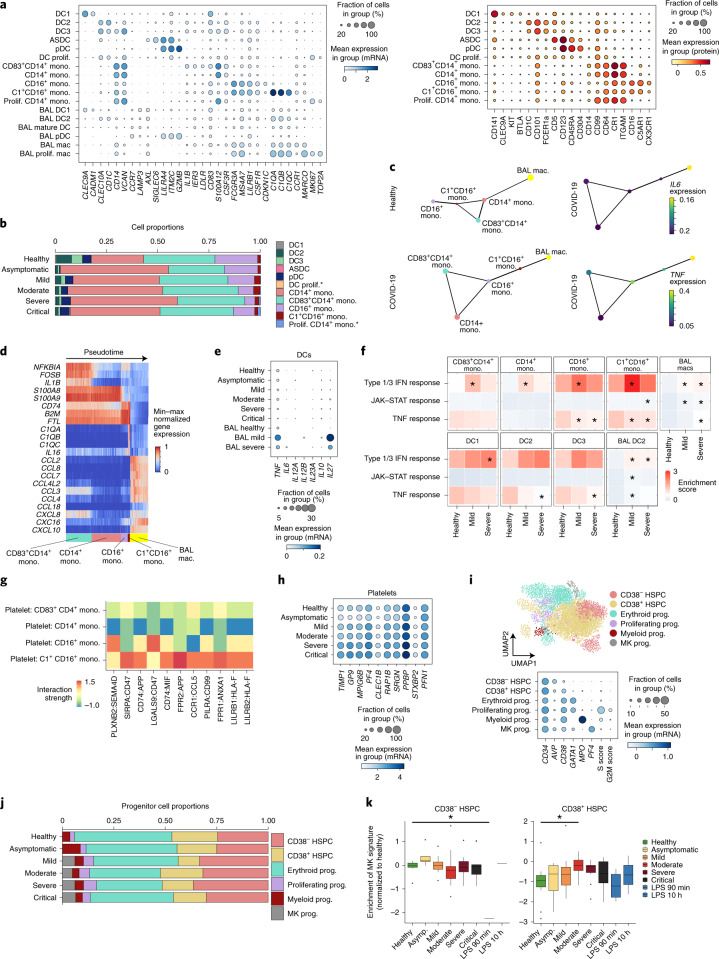
Expansion of complement-expressing nonclassical monocytes and megakaryocyte-primed progenitor cells and increased platelet activation with COVID-19 disease severity. **a**, Dot plots of gene (left) and surface protein (right) expression for myeloid populations. **b**, Bar plot of the proportion of myeloid populations from the Newcastle and London sites. Hypothesis testing was performed using a quasi-likelihood *F*-test comparing healthy controls to individuals with COVID-19. Differentially abundant cell types were determined using a 10% FDR and are marked with an asterisk. **c**, PAGA graph representing connectivity between clusters defined in **a** for healthy (top left) and COVID-19 (bottom left) monocytes and BAL macrophages (mac). Expression of *IL6* (top right) and *TNF* (bottom right) in each cluster along the predicted path for COVID-19 monocytes. **d**, Expression of differentially expressed cytokines between CD83^+^CD14^+^ monocytes and BAL macrophages shown by cells ordered by pseudotime calculated for cells from **c**. **e**, Dot plot of gene expression of DC-derived T cell polarizing cytokines in peripheral blood DC2 cells and mature BAL DCs. **f**, Heat map displaying gene-set enrichment scores for type 1/3 IFN response, TNF response and JAK–STAT signatures in the myeloid populations. Asterisks indicate significance compared to healthy controls. Absolute values and other comparisons are provided in Supplementary Table 7. **g**, Heat map of predicted ligand–receptor interactions between platelets and monocyte subsets, using RNA data. **h**, Dot plot of significant differentially expressed genes between samples from healthy donors and individuals with COVID-19 filtered for platelet activation markers. **i**, UMAP representation of HSPCs (top) and dot plot of gene expression markers used to annotate clusters (bottom). MK, megakaryocyte; prog, progenitor. **j**, Bar chart depicting the proportion of progenitors. **k**, Box plots displaying the enrichment of a megakaryocyte progenitor signature in CD34^+^CD38^+^ HSPCs (right) and CD34^+^CD38^−^ (left), averaged per donor scores. Comparisons were made by an analysis of variance (ANOVA) with pairwise comparisons using Tukey’s test. Asterisks above bars indicate significance and are colored by the severity for which they were compared to. Absolute values are provided in Supplementary Table 8. Boxes denote the interquartile range (IQR), and the median is shown as horizontal bars. Whiskers extend to 1.5 times the IQR, and outliers are shown as individual points (*P* values: CD38-negative cells in healthy versus LPS group (90 min), 0.3 × 10^−3^; CD38-positive cells in healthy versus moderate group, 0.7 × 10^−3^).

**Fig. 3:**
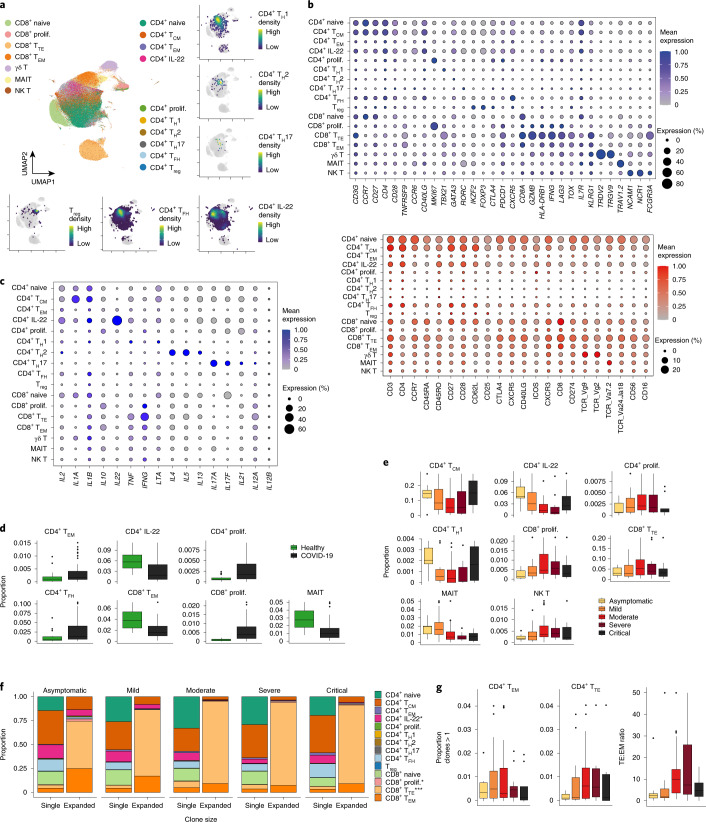
Compositional and clonal analyses of T lymphocytes illustrate the expansion of effector subsets. **a**, UMAP visualization of 309,617 T cells based on gene expression shown and colored by cell type. Insets show the two-dimensional kernel density estimates of select T cell types in UMAP space. **b**, Dot plots of gene (top) and surface protein (bottom) expression for populations shown in **a. c**, Dot plots of gene expression of cytokine genes for populations shown in **a. d**, Box plots of cell type proportions that are differentially abundant between healthy donors and individuals with COVID-19. Boxes denote the IQR, and the median is shown as horizontal bars. Whiskers extend to 1.5 times the IQR and outliers are shown as individual points (*n* = 24 healthy, n = 86 COVID-19 biologically independent samples). **e**, Box plots of the proportion of cell types shown in **a**. Only cell types showing trends of changes by severity status are shown. Boxes denote IQR with median shown as horizontal bars. Whiskers extend to 1.5 times the IQR, and outliers are shown as individual points (*n* = 9 asymptomatic, *n* = 23 mild, *n* = 30 moderate, *n* = 13 severe, *n* = 10 critical biologically independent samples). **f**, Bar plots show the frequency of clonal T cells. Expanded clones denote clonotypes observed more than once. Asterisks indicate significance after multiple-testing correction (logistic regression using two-sided *t*-test with Benjamini–Hochberg FDR correction; CD4^+^ T_CM_ adjusted *P* = 0.119, CD4^+^ T_EM_ adjusted *P* = 0.472, CD4^+^IL-22^+^ adjusted *P* = 0.01, CD4^+^ prolif. adjusted *P* = 0.993, CD4^+^ T_H_1 adjusted *P* = 0.993, CD4^+^ T_FH_ adjusted *P* = 0.109, T_reg_ adjusted *P* = 0.993, CD8^+^ prolif. adjusted *P* = 0.016, CD8^+^ T_TE_ adjusted *P* = 2.49 × 10^−15^, CD8^+^ T_EM_ adjusted *P* = 0.259). **g**, Box plots of the proportion of clonally expanded CD8^+^ T_EM_ cells (left), effector CD8^+^ T cells (middle) and the ratio of effector CD8^+^ T cells to CD8^+^ T_EM_ cells (right). Boxes denote the IQR, and the median is shown as horizontal bars. Whiskers extend to 1.5 times the IQR, and outliers are shown as individual points. Legend is as in **e**.

**Fig. 4:**
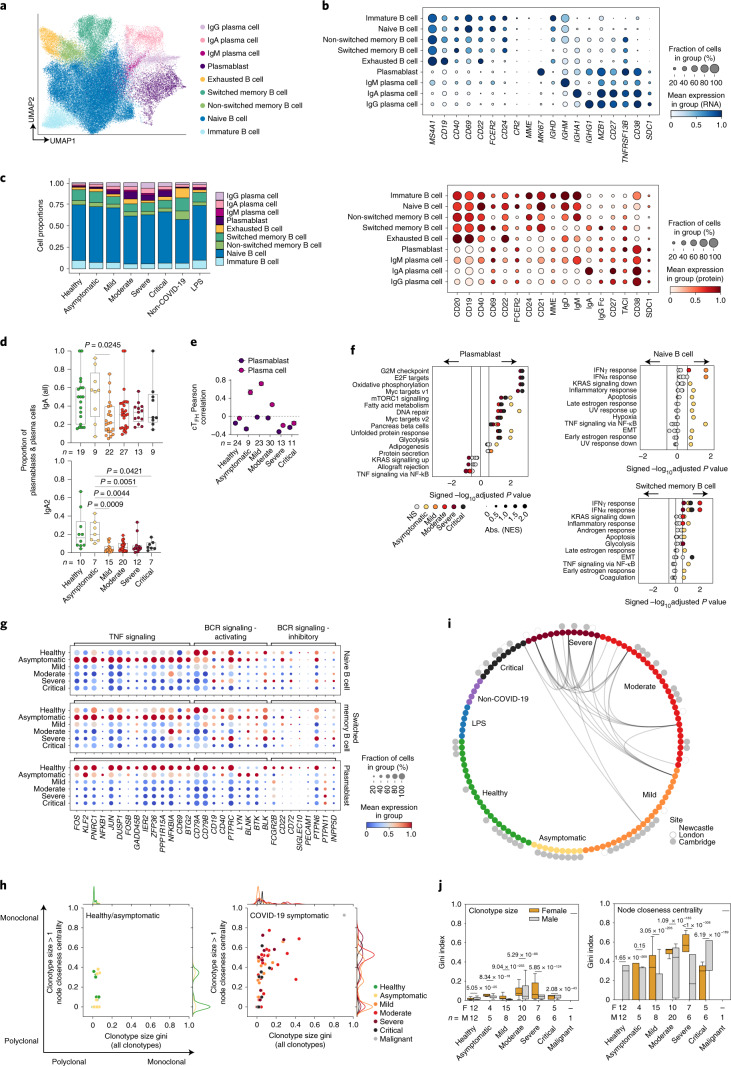
Single-cell analysis of B lymphocytes and BCR repertoire reveal plasmablast expansion and clonality differences between genders. **a**, UMAP visualization of 74,019 cells in the B cell lineage identified from gene expression data. **b**, Dot plots of gene (top) and surface protein (bottom) expression for populations shown in **a. c**, Bar plot of the mean proportion of cell types shown in **a**. **d**, Proportion of total IgA and IgA2 in plasmablast and plasma cells based on BCR data. Kruskal–Wallis test with Benjamini–Hochberg correction. **e**, Coordinated changes between T_FH_ and B cells assessed by differential correlation analysis (empirical *P* ≤ 0.1). Shown is the Pearson correlation (± bootstrap s.e.m.) between T_FH_ proportions and plasmablast or plasma cell (combined); only significant trends are shown. **f**, GSEA of MSigDB hallmark signatures in naive B cells, switched memory B cells and plasmablasts for asymptomatic/symptomatic COVID-19 versus healthy groups. Size of circles indicate (absolute) normalized enrichment score (NES). GSEA (permutation) nominal *P* < 0.05 and FDR < 0.25 denoted by non-gray colored dots. EMT, epithelial–mesenchymal transition; UV, ultraviolet. **g**, Dot plots of genes related to TNF signaling and BCR signaling in naive B cells, switched memory B cells and plasmablasts. Size of circles indicates the percentage of cells expressing the gene, and color gradient corresponds to increasing mean expression value. **h**, Scatterplot of clonotype size by node closeness centrality gini indices with marginal histograms indicating the distribution. Each dot represents an individual. **i**, BCR overlap incidence plot. Nodes correspond to individual donors colored by (inner ring) severity and (outer ring) site from which samples were collected. Edges indicate if at least one cell from each individual displayed an identical combination of heavy and light-chain V and J gene usage with CDR3 similarity allowance (≥85%). **j**, Clonotype size (left) and node closeness centrality gini indices (right) separated by gender. Mann–Whitney *U* test with Benjamini–Hochberg correction between the gender groups within each severity status. Color of adjusted *P* values indicates the gender group with the higher mean value. The box portion of the box plots extends from the 25th to 75th percentiles, whiskers extend from the smallest to largest values, and the middle line corresponds to the median. NS, not significant.

Following Leiden clustering, cells were manually annotated based on the RNA expression of known marker genes supported by surface protein expression of markers employed in flow cytometry to discriminate subpopulations (Extended Data Fig. 1e). We defined 18 cell subsets (Fig. 1b), with an additional 27 cell states identified following subclustering (Figs. 1b, 2a, 3a,b and 4a,b and Supplementary Table 3). Our annotation was further validated using Azimuth, whereby more than 50% of cells were mapped and matched to a unique cluster in 32/33 of the clusters defined in the Azimuth PBMC dataset (Methods; proliferating CD8^+^ T cells mapped across two clusters). Clusters unique to our data included proliferating monocytes, innate lymphoid cell subpopulations and isotype-specific plasma cells (Extended Data Fig. 1f).

**Extended Data Fig. 2:**
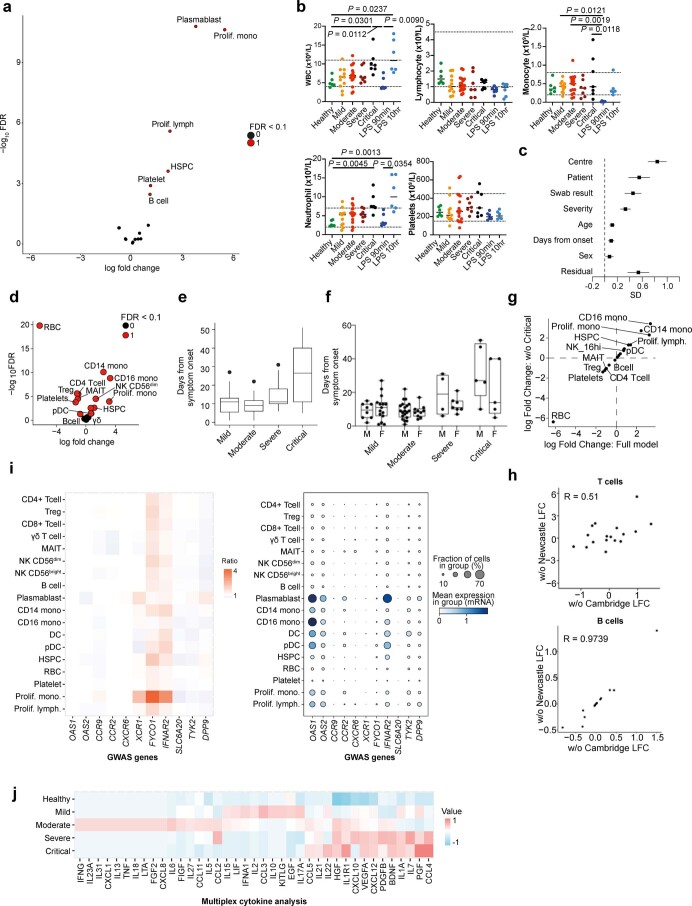
Differential abundance analysis and expression of GWAS hits related to cytokines, chemokines and growth factors. **a**, Volcano plots showing differential abundance testing performed using quasi-likelihood F-test comparing healthy controls to cases for linear trends across disease severity groups. Differentially abundant cell types were determined using a 10% false discovery rate (FDR). **b**, Scatter plots showing blood counts for Newcastle data grouped by severity status (n = 6 healthy and LPS samples; n = 11 mild, n = 17 moderate, n = 7 severe, and n = 7 critical biologically independent samples). Dotted lines mark the normal ranges. Significance determined using Kruskal-Wallis with Dunn’s post hoc corrected for multiple comparison. WBC, whole blood count. **c**, Forest plot showing the standard deviation of each clinical/technical factor estimated by the Poisson generalised linear mixed model. Error bars show the standard error estimated from the Fisher information matrix (n = 130 biologically independent samples) SD, standard deviation. **d**, Volcano plots showing differential abundance testing according to time since symptom onset. Differentially abundant (FDR 10%) points are shown in red. **e**, Box plots displaying the duration of COVID-19 symptoms from the onset by severity (n = 23 mild, n = 30 moderate, n = 11, severe, n = 10 critical biologically independent samples). Boxes denote IQR with median shown as horizontal bars. Whiskers extend to 1.5x the IQR; outliers are shown as individual points. **f**, Box plots displaying the duration of COVID-19 symptoms from the onset split by severity and sex (n = 23 mild, n = 30 moderate, n = 11, severe, n = 10 critical biologically independent samples). Boxes denote IQR with median shown as horizontal bars. Whiskers extend to 1.5x the IQR; outliers are shown as individual points. **g**, Correlated log fold-changes of cell type abundance changes as a function of symptom duration with (x-axis) and without critically ill patients (y-axis). **h**, Differential abundance testing with a leave-one-out analysis for the T cells (top) of B cells (bottom) (FDR10%). **i**, Heat map displaying fold change over healthy (left) and dot plot of gene expression where the colour is scaled by mean expression and the dot size is proportional to the percent of the population expressing the gene (right) for genes associated with COVID-19 identified in recent GWAS studies^18,19^ for the cell populations in Fig. 1b. **j**, Heat map displaying normalised values of cytokine, chemokine and growth factors in serum of patients with COVID-19.

We observed a relative expansion of proliferating lymphocytes, proliferating monocytes, platelets and mobilized hematopoietic stem and progenitor cells (HSPCs) with worsening disease. Plasmablasts and B cells were expanded in severe and critical disease (Fig. 1c and Extended Data Fig. 2a). These changes matched trends in clinical blood cell counts (Extended Data Fig. 2b and Supplementary Table 4). To assess the broader impacts of patient characteristics and clinical metadata on the altered proportion of cell types/states, we used a Poisson linear mixed model (Methods), which predicted the COVID-19 swab result (Bonferroni-corrected logistic regression (BF-corrected LR), *P* = 1.1 × 10^−3^; Methods), disease severity at blood sampling (BF-corrected LR, *P* = 8.9 × 10^−8^) and center (contributed by increased red blood cells (RBCs) and reduced monocytes in the Cambridge patient cohort; (BF-corrected LR, *P* = 2.0 × 10^−142^) as the main contributing factors among seven different clinical/technical factors (Extended Data Fig. 2c). PBMC composition varied depending on symptom duration, with increased relative frequency of plasmacytoid dendritic cells (pDCs), natural killer (NK) cells, CD14^+^ and CD16^+^ monocytes (false discovery rate (FDR), 10%) and decreased relative frequencies of B cells, regulatory T (T_reg_) cells, RBCs, platelets and CD4^+^ T cells, with a longer symptomatic interval before hospital admission (Extended Data Fig. 2d). These changes may be due to a subset of individuals in the critically ill category who reported a longer time since symptom onset, consistent with a protracted course of infection in critical disease (Extended Data Fig. 2e,f). However, concordant changes in immune cell composition were observed when excluding patients with either the longest symptom durations (>24 d) or critical disease (Extended Data Fig. 2g), indicating that disease severity changes were not driven by symptom duration. Cell abundance results were also in agreement when performing a leave-one-out analysis (Extended Data Fig. 2h).

We observed expression of type I/III interferon response genes in monocytes, DCs and HSPCs across the spectrum of COVID-19 severity, but not in individuals challenged with IV-LPS, in keeping with the importance of type I and III interferons in innate immune responses to viral infection (Fig. 1d). Type I/III interferon response-related genes were recently implicated in genome-wide association studies (GWAS) for COVID-19 susceptibility^18,19^. *IFNAR2* was both upregulated in individuals with COVID-19 compared to healthy controls in most circulating cell types and highly expressed by plasmablasts, monocytes and DCs (Extended Data Fig. 2i).

Multiplexed analysis of 45 proteins in serum showed two contrasting profiles between mild/moderate and severe/critical patients. CCL4, CXCL10, interleukin (IL)-7 and IL-1α were associated with severe and critical disease, suggesting an augmented drive for monocyte and NK lymphocyte recruitment, as well as support for T cell activity/pathology (Extended Data Fig. 2j and Supplementary Table 5).

We used Cydar^20^ to characterize the immune landscape changes with disease severity based on surface protein expression by dividing cells into phenotypic hyperspheres. We quantified the number of cells from each severity group within the hyperspheres, identifying 608 hyperspheres that differed significantly in abundance with increasing severity (spatial FDR < 0.05; Fig. 1e). Differentially abundant hyperspheres were present in all major immune compartments. Notably, we found an increase in B cells (CD19^+^/CD20^+^), plasma cells (CD38^+^) and HSPCs (CD34^+^), as well as remodeling of the myeloid compartment^13^ (Fig. 1e).

### Mononuclear phagocytes and HSPC changes

**Extended Data Fig. 3:**
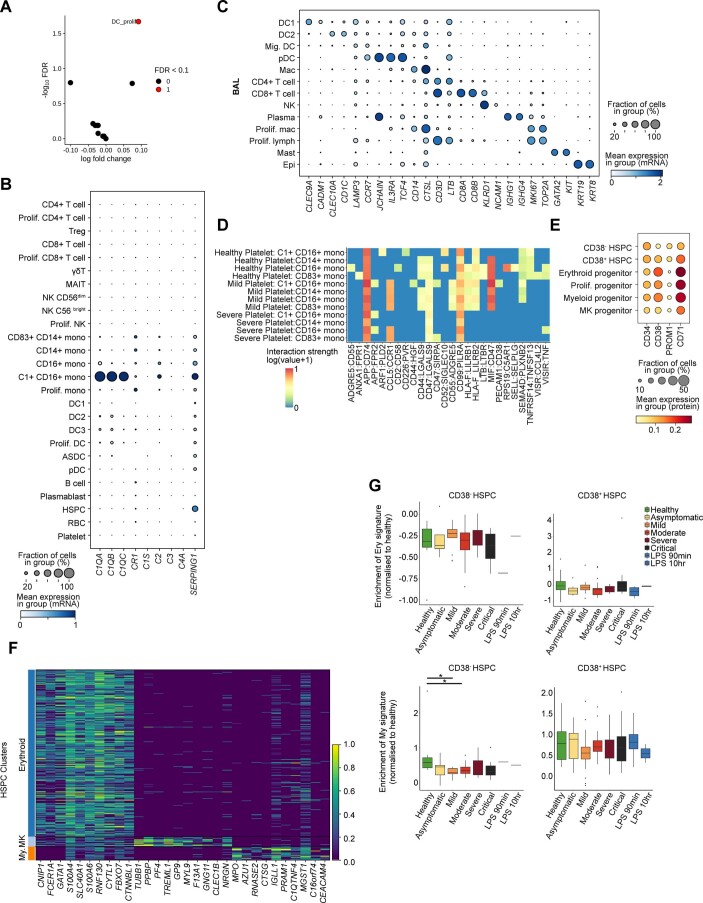
Myeloid comparisons with bronchial alveolar lavage dataset and receptor-ligand interaction analysis between megakaryocyte, myeloid and progenitor cells. **a**, Volcano plot showing differential abundance testing according to time since symptom onset for the myeloid populations. Differentially abundant (FDR 10%) points are shown in red. **b**, Dot plots of gene expression of C1 complement components for cells in Fig. 1b where the colour is scaled by mean expression and the dot size is proportional to the percent of the population expressing the gene. **c**, Dot plots of gene expression of a recently published BAL dataset (accession number GSE145926^22^) for genes in Fig. 2a where the colour is scaled by mean expression and the dot size is proportional to the percent of the population expressing the gene. **d**, Heat map of CellPhoneDB predicted ligand:receptor interactions between platelets and monocyte subsets, based on the protein data. **e**, Dot plots of expression of protein markers used to annotate clusters in Fig. 2i. MK, Megakaryocyte **f**. Heatmap of differentially expressed genes between megakaryocyte, myeloid and erythroid progenitor clusters. MK, megakaryocyte; My, myeloid. **g**, Box plots displaying enrichment of an erythroid signature (top) and a myeloid signature (bottom) found in CD34^−^CD38^−^ (left) and CD34^+^CD38^+^ HSPCs (right), separated by severity. Asterisks above bars indicate significance and are coloured by the severity for which they were compared to. Absolute values are provided in Supplementary Tables 8 and 9. (n = 120 biologically independent samples, n = 3297 cells in 1 experiment). Boxes denote IQR with median shown as horizontal bars. Whiskers extend to 1.5x the IQR; outliers are shown as individual points (n = 24 healthy, n = 86 COVID-19 biologically independent samples) (p-values: Myeloid signature in CD38 negative HSPCs, Healthy vs. Mild: 0.8 x 10^-3^, Healthy vs Moderate 0.02).

Transcriptome and surface proteome analysis of blood mononuclear phagocytes (MPs) identified known DC subsets (pDC, ASDC (*AXL*^+^*SIGLEC6*^+^ DC), DC1, DC2 and DC3) and several monocyte states (Fig. 2a,b). Three CD14^+^ monocyte states were present (proliferating, classical CD14^+^ and activated CD83^+^) in addition to two CD16^+^ monocyte states (nonclassical CD16^+^ and C1QA/B/C^+^CD16^+^; Fig. 2a,b). Proliferating monocytes and DCs expressing *MKI67* and *TOP2A* were increased with disease severity (Fig. 2a,b). In contrast, the frequencies of DC2 and DC3 were reduced. Proliferating monocytes, previously reported by flow cytometry analysis of blood from patients with COVID-19^14^, transcriptionally resembled CD14^+^ monocytes and was the only population to change significantly with symptom duration. (Fig. 2a,b and Extended Data Fig. 3a). Proliferating DCs resembled the DC2 subset (Fig. 2a,b). Rare *C1QA/B/C*-expressing CD16^+^ monocytes were the only source of C1 complement components (Fig. 2b and Extended Data Fig. 3b).

We previously demonstrated egress of blood DCs and monocytes to the alveolar space with rapid acquisition of a lung molecular profile following human inhalational LPS challenge^21^. To investigate the relationship between circulating and lung alveolar MPs in COVID-19, we compared the transcriptome profile of blood DCs and monocytes with their bronchoalveolar lavage (BAL) counterparts using recently published data (GSE145926)^22^ (Extended Data Fig. 3d). Partition-based graph abstraction (PAGA) suggested transcriptional similarity between circulating CD14^+^ monocytes and BAL macrophages in health, aligning with recent data demonstrating that BAL macrophages can arise from circulating CD14^+^ monocytes (Fig. 2c)^23^. In COVID-19, there was greater transcriptional similarity between BAL macrophages and circulating C1QA/B/C^+^CD16^+^ monocytes (Fig. 2c), suggesting a differential origin of alveolar macrophages in healthy donors and individuals with COVID-19. Both BAL macrophages and C1QA/B/C^+^CD16^+^ monocytes express *FCGR3A* and *C1QA/B/C* and type I interferon response genes (Fig. 2a). Myeloid hyperinflammation causing lung and peripheral tissue damage via secretion of inflammatory cytokines such as IL-6 and tumor necrosis factor (TNF) in COVID-19 has been reported and in our analysis were primarily expressed by tissue rather than blood MPs (Fig. 2c). Genes differentially expressed by blood monocytes identified *S100A8*, previously reported in COVID-19 as contributing to the cytokine storm in severe infection^24^. BAL macrophages expressed leukocyte-recruiting chemokines including *CCL2*, *CCL4*, *CCL7* and *CCL8* (Fig. 2d).

Tissue DCs respond to local inflammation and pathogen challenge by migrating to the draining lymph node to activate naïve T cells. BAL macrophages contain a population of mature, migratory DCs that express *CCR7* and *LAMP3* but downregulate DC-specific markers, such as *CD1C* and *CLEC9A* (Extended Data Fig. 3c). These migratory DCs express *IL10* in health, but *TNF* and the common IL-12 and IL-23 subunit *IL12B* in COVID-19, suggesting altered capacity for T cell polarization (Fig. 2e). In peripheral blood, C1QA/B/C^+^CD16^+^ monocytes expressed the highest amount of type 1 IFN response genes compared to all myeloid cells (Fig. 2f and Supplementary Tables 6 and 7). We detected minimal TNF-mediated or IL-6-mediated JAK–STAT signaling activation in circulating monocytes and DCs, but this was upregulated by COVID-19 BAL MPs (Fig. 2f, Supplementary Tables 6 and 7).

Coagulation abnormalities and monocyte–platelet aggregates have been previously reported in COVID-19 (refs. ^25,26^), leading us to investigate predicted receptor–ligand interactions between monocytes and platelets using the CellPhoneDB repository. The expression levels of *SIRPA:CD47, FPR1:ANXA1, FPR2:APP* between monocytes:platelets were highest in the C1QA/B/C^+^CD16^+^ monocytes (Fig. 2g). Using protein data, we identified ICAM1 interactions on platelets with CD11a/b/c/CD18 primarily on C1QA/B/C^+^CD16^+^ monocytes and CD16^+^ monocytes (Extended Data Fig. 3d), accompanied by increased expression of surface proteins indicative of platelet activation (Fig. 2h).

Our large dataset of 781,123 PBMCs allowed us to interrogate 3,297 CD34^+^ HSPCs. Leiden clustering and uniform manifold and projection (UMAP) visualization showed a cloud-like representation, consistent with a stem/progenitor cell landscape previously described for bone marrow HSPCs^27^ (Fig. 2i and Extended Data Fig. 3e). Absence of CD38 mRNA and protein expression marks the most immature cells within the CD34^+^ compartment, while expression of markers such as *GATA1*, *MPO* and *PF4* characterizes distinct erythroid, myeloid and megakaryocytic progenitor populations, respectively (Fig. 2i). Accordingly, we were able to annotate six transcriptional clusters as CD34^+^CD38^−^ HSPCs, CD34^+^CD38^+^ early progenitor HSPCs and CD34^+^CD38^+^ erythroid, megakaryocytic and myeloid progenitors, as well as a small population distinguished by the expression of cell cycle (S phase) genes (Fig. 2i). Megakaryocyte progenitors were absent in healthy and asymptomatic individuals but made up ~5% of CD34^+^ cells in all symptomatic individuals (Fig. 2j). As peripheral blood is not a site for hematopoiesis^28^, this finding likely reflects COVID-19-mediated perturbation of normal homeostatic functioning of the bone marrow HSPC compartment.

In light of our earlier observations of platelet activation and enhanced C1QA/B/C^+^CD16^+^ monocyte–platelet interactions (Fig. 2g,h), the appearance of CD34^+^ megakaryocyte progenitors was of particular interest, as it suggested a rebalancing of the stem/progenitor cell compartment. To further explore this hypothesis, we generated differential gene expression signatures between the megakaryocyte, myeloid and erythroid progenitor clusters to interrogate potential early activation or priming in the most immature HSPC clusters (Extended Data Fig. 3f). We observed enrichment of the megakaryocyte progenitor signature in the CD38^+^ HSPC populations in moderate COVID-19 compared to the healthy condition (Fig. 2k and Supplementary Table 8), but no enrichment of erythroid or myeloid signatures in either CD38^−^ or CD38^+^ HSPCs (Extended Data Fig. 3g and Supplementary Table 8). Our earlier observation of increased platelet activation within the context of normal platelet counts (Fig. 2h and Extended Data Fig. 2b) is thus consistent with a model whereby a rebalancing of the HSPC compartment toward megakaryopoiesis may be compensating for peripheral platelet consumption in COVID-19.

### T lymphocytes and T cell receptor changes

**Extended Data Fig. 4:**
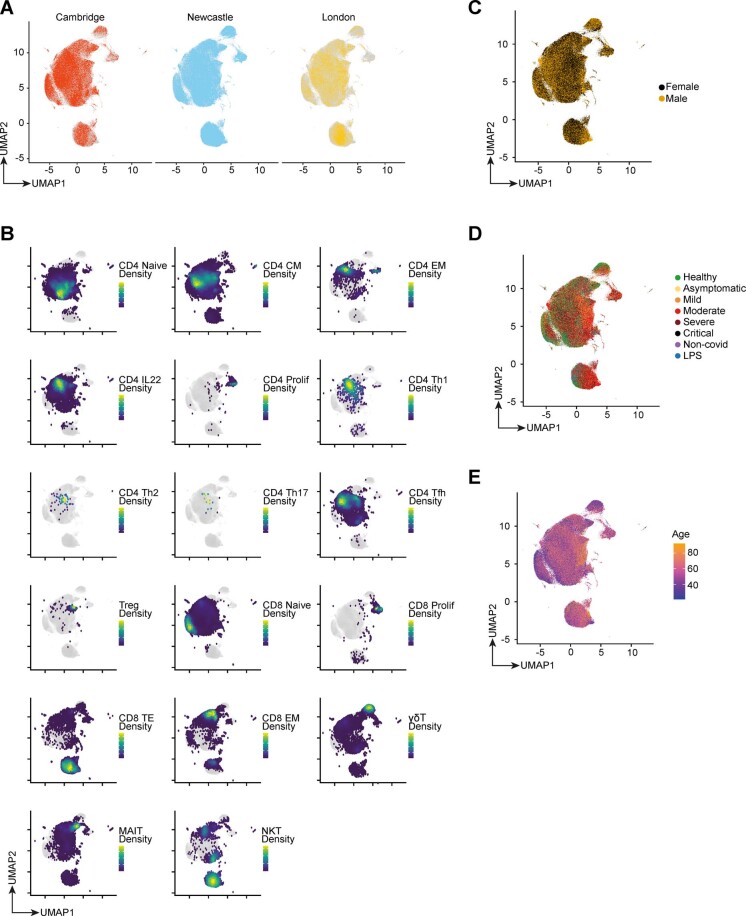
UMAP embedding of T lymphoid clusters showing cell type estimation and covariates. **a**, UMAP visualisation of 309,617 T cells separated by sources of donors. **b**, UMAP visualisation showing 2-dimensional kernel density estimates of each T cell type in UMAP space. **c**.-**e**. UMAP visualisation of T cells coloured by gender (**c**), disease severity status (**d**) and age (**e**).

Fine-resolution clustering of mRNA profiles revealed 11 initial clusters of CD4^+^ T cells, CD8^+^ T cells and innate-like T cells including γδ T cells, NK T cells and mucosal-associated invariant T (MAIT) cells (Fig. 3a and Extended Data Fig. 4). Annotations were refined further using RNA expression of effector cytokines and surface protein expression (Fig. 3a–c).

**Extended Data Fig. 5:**
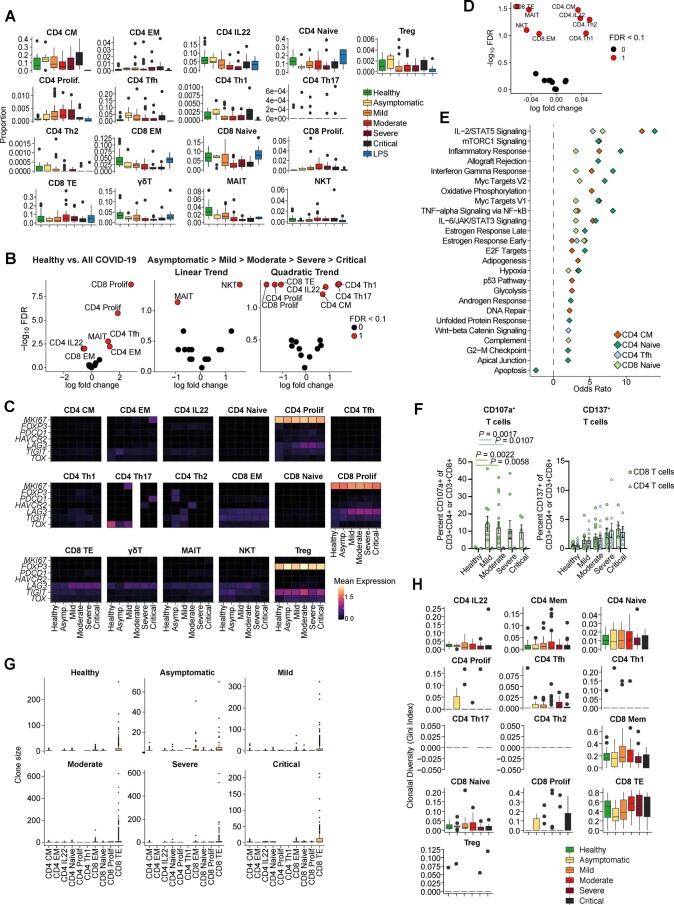
Differential abundance testing, gene set enrichment analysis and clonal diversity analysis of T lymphoid compartment. **a**, Box plots showing the proportion of cell types shown in Fig. 3a, n = 108 biologically independent samples. Boxes denote IQR with median shown as horizontal bars. Whiskers extend to 1.5x the IQR; outliers are shown as individual points. **b**, Volcano plots showing results of differential abundance testing. Abundance counts were modelled either comparing healthy vs. COVID-19, or as a function of severity. Hypothesis testing was performed using quasi-likelihood F-test comparing healthy controls to cases, or for either a linear or quadratic trend across disease severity. Differentially abundant cell types were determined using a 10% false discovery rate (FDR). **c**, Heatmaps showing mean expression levels across T cell subsets for suppressive (*FOXP3*), proliferating (*MKI67*) and exhaustion markers (*PDCD1, HAVCR2, LAG3, TIGIT, TOX*). Columns denote the mean log-normalised expression within each severity category and healthy controls. **d**, Volcano plot showing differential abundance testing according to time since symptom onset for the T cell populations. Differentially abundant (FDR 10%) points are shown in red. **e**, Gene set enrichment (Methods) in each T cell type based on differential gene expression (DGE) analysis was performed across COVID-19 disease severity groups, ordered from healthy > asymptomatic > mild > moderate > severe > critical. Statistically significant DE genes were defined with FDR < 1%. Significant enrichments were defined with 10% FDR. **f**, Bar plots showing percent (mean + /- SEM) of CD3^+^CD4^+^ (blue) and CD3^+^CD8^+^ (green) T cells expressing CD107a (left) and CD137 (right) in response to SARS-CoV-2 S peptide stimulation (n = 12 healthy, n = 10 mild, n = 17 moderate, n = 9 severe, and n = 5 critical biologically independent samples). Significance determined using Kruskal-Wallis with Dunn’s post-hoc corrected for multiple comparison. **g**, Box plots showing clone size distribution for each T cell subset (n = 9 asymptomatic, n = 19 mild, n = 29 moderate, n = 12 severe, n = 10 critical biologically independent samples). Boxes denote IQR with median shown as horizontal bars. Whiskers extend to 1.5x the IQR; outliers are shown as individual points. **h**, Box plots slowing clonal diversity for each T cell subset (n = 22 healthy, n = 12 asymptomatic, n = 22 mild, n = 31 moderate, n = 14 severe, n = 13 critical biologically independent samples). Boxes denote IQR with median shown as horizontal bars. Whiskers extend to 1.5x the IQR; outliers are shown as individual points. Source data

Cellular composition of the T cell compartment varied between healthy and infected groups (Fig. 3d). Based on their relative proportions and differential abundance testing (FDR 10%), we found activated CD4^+^ T cells expressing *IL22*, circulating follicular helper T (T_FH_) cells, type 1 helper T (T_H_1) cells, CD8^+^ effector memory T (T_EM_) cells and MAIT cells relatively enriched in individuals with asymptomatic and mild infection, with NKT, proliferating CD8^+^ and CD4^+^, and CD8^+^ terminal effector T (T_TE_) cells enriched in individuals with more severe infection (Fig. 3e and Extended Data Fig. 5a,b). Treating disease severity as an ordinal variable (Methods), multiple cell populations displayed nonlinear differences across disease severity (proliferating CD4^+^ and CD8^+^ T cells, CD8^+^ T_TE_, CD4^+^ T_H_1, CD4^+^ T_H_17, CD4^+^ central memory T (T_CM_) and IL-22^+^CD4^+^ T cells), illustrating the complex compositional changes to peripheral T cells that occur with COVID-19 (Fig. 3e and Extended Data Fig. 5b). IL-22-expressing CD4^+^ T cells seen in asymptomatic and mild disease could be associated with tissue-protective responses that may restrict immunopathology (Fig. 3e) as previously shown for IL-22 in influenza A virus infection^29^ and lower viral load in COVID-19 patients’ lungs^30^. Proliferating CD4^+^ and CD8^+^ T cells coexpressed exhaustion marker genes *LAG3* and *TOX* (Extended Data Fig. 5c), in keeping with previous studies of patients with severe COVID-19 (ref. ^5^). In contrast to disease severity, CD4^+^ T_H_1, CD4^+^ T_H_2, CD4^+^IL-22^+^ and CD4^+^ T_CM_ cells were enriched among individuals with longer symptom duration, while effector populations with a cytotoxic phenotype (CD8^+^ T_TE_, CD8^+^ T_EM_, MAIT and NK T cells) were enriched in individuals with shorter symptom duration (Extended Data Fig. 5d).

Differential gene expression analysis across disease severity (FDR 1%) and gene-set enrichment analysis (GSEA) identified pathways associated with inflammation and T cell activation across multiple subsets, including IL-2–STAT5 signaling, mTORC1 signaling, inflammatory response, IFNγ response, and IL-6–JAK–STAT3 signaling (Extended Data Fig. 5e). The increased activation and cytotoxic phenotype in T cells from individuals with COVID-19 was functionally validated by flow cytometry analysis of PBMCs stimulated ex vivo with SARS-CoV-2 peptide showing upregulation of CD137 and CD107α (Extended Data Fig. 5f).

T cell receptor (TCR) clonality analysis showed that effector CD8^+^ T cells were the most clonally expanded (odds ratio (OR) (95% confidence interval (CI)) 1.81 (1.58–2.10)), *P* = 2.49 × 10^−15^) and their relative proportion increased with disease severity (Fig. 3f,g and Extended Data Fig. 5g,h and Supplementary Tables 6 and 7). Conversely, the relative proportion of clonally expanded effector memory CD8^+^ T_EM_ cells decreased in individuals with more severe disease (OR (95% CI) 0.87 (0.72–1.04), *P* = 0.26; Fig. 3f,g and Supplementary Tables 9 and 10). These clonal alterations were primarily driven by severity rather than differences in symptom duration for more severely ill patients, as CD8^+^ T_EM_ clones expanded in individuals who had longer symptom duration, in line with a more developed infection trajectory in these individuals (per day, OR (95% CI) 1.02 (1.01–1.03), *P* = 2.66 × 10^−10^). The ratio of effector CD8^+^ T cells to CD8^+^ T_EM_ cells (TE:EM ratio) correlated with disease severity (linear model *β* 2.97, *P* = 2.92 × 10^−18^; Fig. 3g and Supplementary Tables 9 and 10), suggesting that CD8^+^ T cell differentiation outcome may contribute to both antiviral protection and immunopathology, as previously reported in animal models^31^, although bystander T cell activation cannot be excluded.

### B lymphocytes and B cell receptor changes

**Extended Data Fig. 6:**
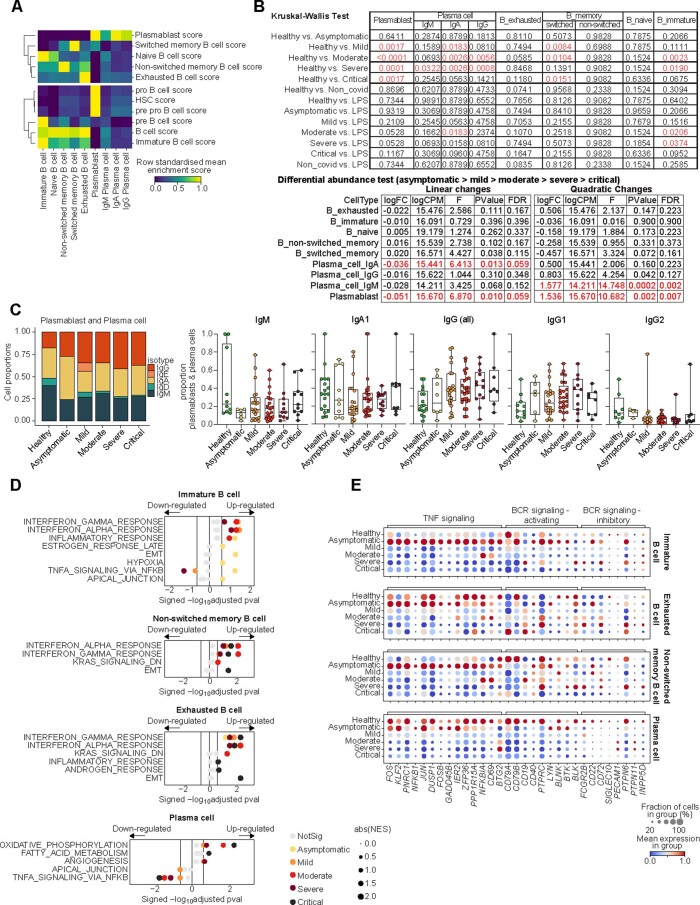
Single-cell analysis of B lymphocytes proportion and gene set differences across COVID-19 severity. **a**, Heatmap of mean gene set enrichment scores of (top) adult peripheral blood B cell signatures^32^ and (bottom) bone marrow B cell signatures^68^. Row enrichment value is scaled from 0-1 and colour gradient corresponds to increasing mean enrichment score. **b**, (Top) Kruskal-Wallis test results with Benjamini-Hochberg false discovery correction for cell type proportion differences in plasmablast/plasma cells between severities. Significance is denoted by Benjamini-Hochberg corrected *P* < 0.05 (red text). (Bottom) Cell type abundance counts were modelled as a function of severity. Hypothesis testing was performed using quasi-likelihood F-test comparing asymptomatic to symptomatic covid, for either a linear or quadratic trend across severities. Differentially abundant cell types were determined using a 10% false discovery rate (FDR). **c**, (Left) Bar plot showing the mean proportion of plasmablast/plasma cells expressing IgA, IgD, IgE, IgG or IgM, based on V(D)J information. (Right) Proportion of IgM, IgA1, total IgG, IgG1, and IgG2 subclass in plasmablast and plasma cells. Kruskal-Wallis test results with Benjamini-Hochberg false discovery correction for cell type proportion differences in plasmablast/plasma cells between severities showed no significant differences. n = 10, 6, 21, 24, 11, 11 (IgM), n = 18, 9, 21, 27, 12, 9 (IgA1), n = 16, 8, 23, 27, 13, 8 (IgG (all)), n = 11, 6, 22, 25, 11, 8 (IgG1) and n = 8, 4, 16, 23, 9, 7 (IgG2) biologically independent patients/samples for healthy, asymptomatic, mild, moderate, severe and critical respectively. **d**, GSEA of pathways in B cell subsets for asymptomatic/symptomatic COVID versus healthy. Size of circles indicate (absolute) normalised enrichment score (NES). Pathways were considered statistically significant if GSEA (permutation) nominal *P* < 0.05 and FDR < 0.25 (denoted by non-grey coloured dots; boundary lines in the middle marks FDR = 0.25). EMT, Epithelial-mesenchymal transition. **e**, Dot plots of TNF signalling molecules, activating and inhibitory BCR signaling molecules (5’ gene expression data) in immature B cells, non-switched memory B cells, ‘exhausted’ B cells and plasma cells. Size of circles indicate percent of cells expressing the gene and colour gradient corresponds to increasing mean expression value.

Re-clustering of B cells and plasma cells identified nine clusters that were annotated according to canonical marker expression (Fig. 4a,b), and previously published transcriptional signatures (Extended Data Fig. 6a). This included immature, naïve, switched and non-switched memory B cells, and a cluster of cells that enriched for markers previously described in exhausted memory B cells^32,33^ (Fig. 4a,b and Extended Data Fig. 6a). We also found a large population of plasmablasts with negative expression of CD19 and CD20, with high expression of the proliferation marker *MKI67*, consistent with previous reports on severe SARS-CoV-2 infection^15,24^, as well as IgM^+^, IgG^+^ and IgA^+^ plasma cells (Fig. 4a,b). In individuals with symptomatic COVID-19, there was a significant expansion of plasmablasts and plasma cells (Fig. 4c and Extended Data Fig. 6b). The magnitude of this expansion increased from mild to moderate disease but was attenuated in severe to critical disease. This observation persisted even after accounting for days from symptom onset (Extended Data Fig. 6b). IgA^+^ cells were decreased in individuals with symptomatic COVID-19 due to a significant decrease of the IgA2 subclass (Fig. 4d and Extended Data Fig. 6b,c), suggestive of the maintenance of an effective mucosal humoral response in asymptomatic individuals. In parallel, we observed the greatest expansion of circulating follicular helper T (cT_FH_) cells in asymptomatic individuals and a strong positive correlation between cT_FH_ cells and plasma cells in individuals with asymptomatic/mild disease that was lost with increasing disease severity (Figs. 3e and 4e and Extended Data Fig. 5a,b). Together, this suggests the presence of coordinated T cell and B cell responses in asymptomatic and mild disease, generating effective antiviral humoral immunity that becomes uncoupled in severe and critical disease. This is consistent with previous findings relating to the requirement of T_FH_ cells for optimal antibody responses and high-quality neutralizing antibodies in viral infection^34^.

GSEA analysis identified interferon alpha response and interferon gamma response pathway genes enriched in all B cell subsets in individuals with COVID-19, and this was more marked in those with asymptomatic or mild disease, and attenuated in severe and critical disease (Fig. 4f and Extended Data Fig. 6d). The magnitude of type 1 interferon transcriptional response in B cells mirrored serum IFNα levels, which were highest in individuals with mild disease (Extended Data Fig. 2j), suggesting that the low expression of IFN response genes in B cells in severe or critical disease does not reflect an inability of B cells to respond to IFNα, but rather attenuation of IFNα. This may be because the initial antiviral response has waned in patients with severe or critical disease or because these individuals fail to sustain adequate IFNα production by myeloid cells and pDCs following symptom onset as previously reported^7^. Longitudinal sampling would be required to distinguish these two possibilities.

In asymptomatic individuals, TNF signaling via nuclear factor kappa B (NF-κB) pathway genes was enriched in immature, naïve and switched memory B cells, but decreased in immature B cells and plasma cells in critical and severe disease (Fig. 4f and Extended Data Fig. 6d). Assessment of the leading-edge genes in this pathway demonstrated their markedly higher expression in all B cell and plasmablast/cell subsets in asymptomatic individuals with COVID-19 compared with those with symptomatic disease (Fig. 4g and Extended Data Fig. 6e). TNF was barely detectable in COVID-19 serum samples and highest in individuals with moderate disease (Extended Data Fig. 2j), suggesting that another cytokine, for example IL-6, or stimulus may be responsible for NF-κB activation in asymptomatic individuals with COVID-19.

Hypoxia pathway genes were enriched in immature and naïve B cells only in asymptomatic individuals (Fig. 4f and Extended Data Fig. 6d). Since these individuals are unlikely to be hypoxic (given their lack of symptoms), we postulated that this signature may reflect another hypoxia inducible factor-activating stimulus, which includes B cell receptor (BCR) cross-linking^35^. We assessed the expression of genes associated with BCR activation, such as *CD79A* and *CD79B*, and downstream kinases such as *BTK* in B cell subsets. Overall, BCR activation-associated genes were most highly expressed in B cells in healthy controls, followed by asymptomatic individuals with COVID-19, with lower expression observed in all symptomatic COVID-19 groups (Fig. 4g and Extended Data Fig. 6e). BCR activation threshold is also modulated by immune tyrosine inhibitory motif-containing receptors that recruit phosphatases, increasing the activation threshold of B cells^36^. BCR inhibitory gene expression was limited, but *CD22* was detectable across B cell subsets in asymptomatic COVID-19, while *FCGR2B*, *CD72* and *PTPN6* expression was evident in B cells in severe COVID-19 (Fig. 4g and Extended Data Fig. 6e). Together, this analysis suggests that B cells in asymptomatic individuals with COVID-19 and those with mild disease have a more pronounced response to interferons, increased NF-κB activation and a higher expression of genes associated with BCR activation signaling, suggesting a potential for greater BCR activation. Longitudinal analysis of patient samples will be required to establish if this is due to avid responses early in disease that prevent progression to a more severe phenotype or the immune response in early disease.

Following activation, B cells differentiate into antibody-producing plasma cells, accompanied by a progressive increase in oxidative metabolism^37,38^. We observed differences in metabolic gene pathway expression in plasmablasts and plasma cells between disease severity categories, with enrichment of oxidative phosphorylation pathway genes in all disease groups, and a relative increase in glycolysis pathway genes in asymptomatic patient plasmablasts when compared to symptomatic disease groups (Fig. 4f and Extended Data Fig. 6e).

**Extended Data Fig. 7:**
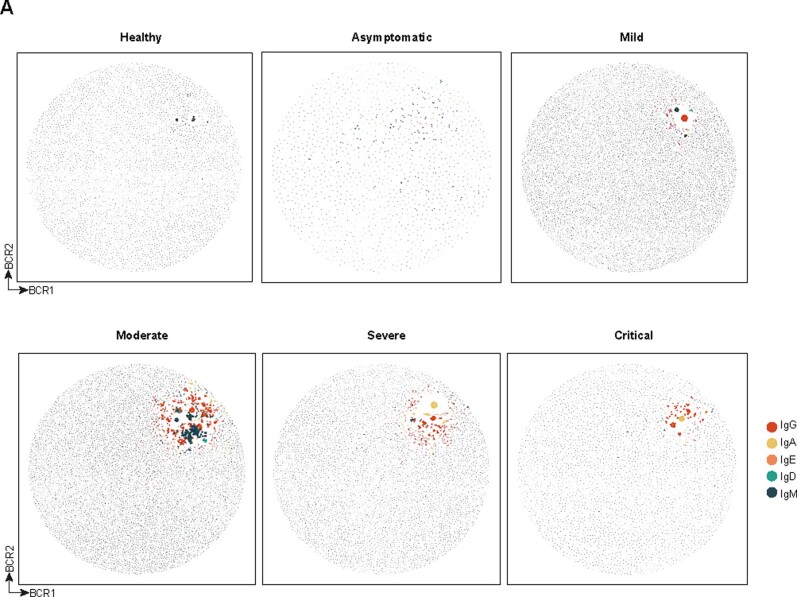
Single-cell BCR networks across COVID-19 severity. Single-cell BCR network plots for each severity status coloured by heavy chain isotype class (IgM, IgD, IgA, IgE, or IgG). Each circle/node corresponds to a single B cell with a corresponding set of BCR(s). Each clonotype is presented as a minimally connected graph with edge widths scaled to 1/*d* + 1 for edge weight *d* where *d* corresponds to the total (Levenshtein) edit distance of BCRs between two cells. Size of nodes is scaled according to increasing node closeness centrality scores that is nodes that are highly central to a clonotype network will be larger.

**Extended Data Fig. 8:**
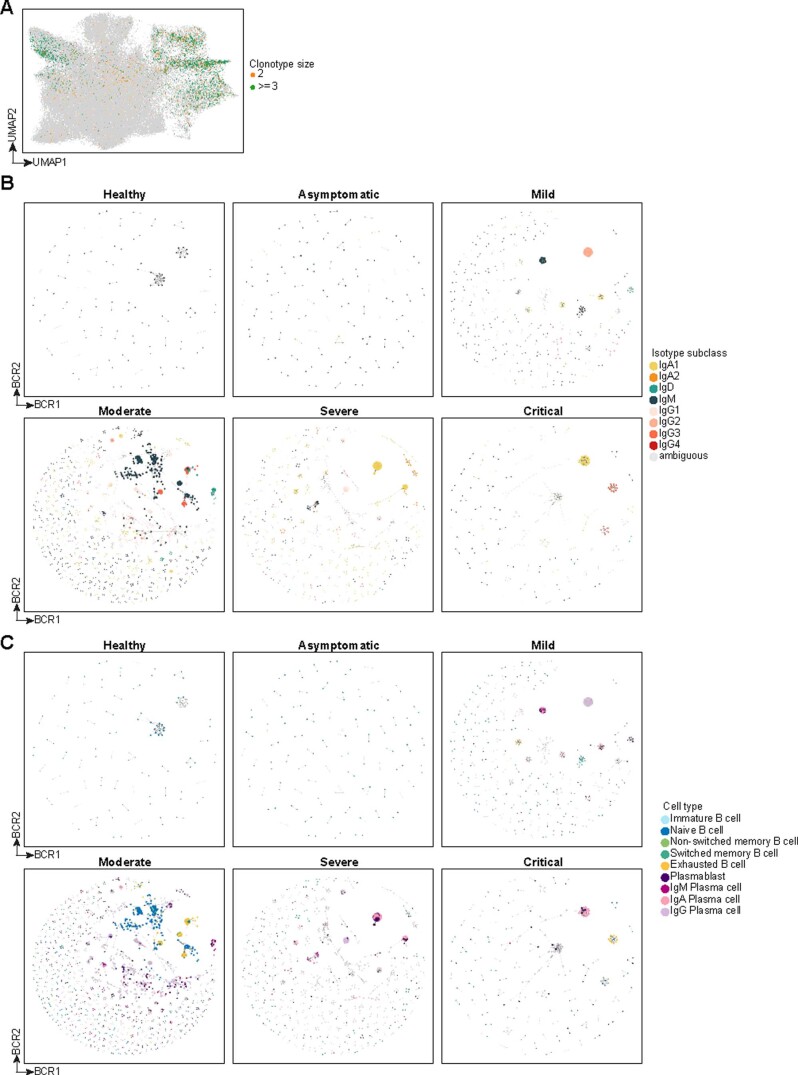
Single-cell BCR clonotype expansion analysis across COVID-19 severity. **a**, UMAP visualisation of B cell lineage and coloured by clonotype size in the V(D)J data. Only expanded clonotypes are coloured (clonotype size > 2). **b**, Single-cell BCR network plots for each severity status coloured by assigned cell type. **c**, Single-cell BCR network plots for each severity status coloured by heavy chain isotype subclass (IgM, IgD, IgA1, IgA2, IgE, IgG1, IgG2, IgG3 or IgG4). Each circle/node corresponds to a single B cell with a corresponding set of BCR(s). Each clonotype is presented as a minimally connected graph with edge widths scaled to 1/*d* + 1 for edge weight *d* where *d* corresponds to the total (Levenshtein) edit distance of BCRs between two cells. Size of nodes is scaled according to increasing node closeness centrality scores that is nodes that are highly central to a clonotype network will be larger.

**Extended Data Fig. 9:**
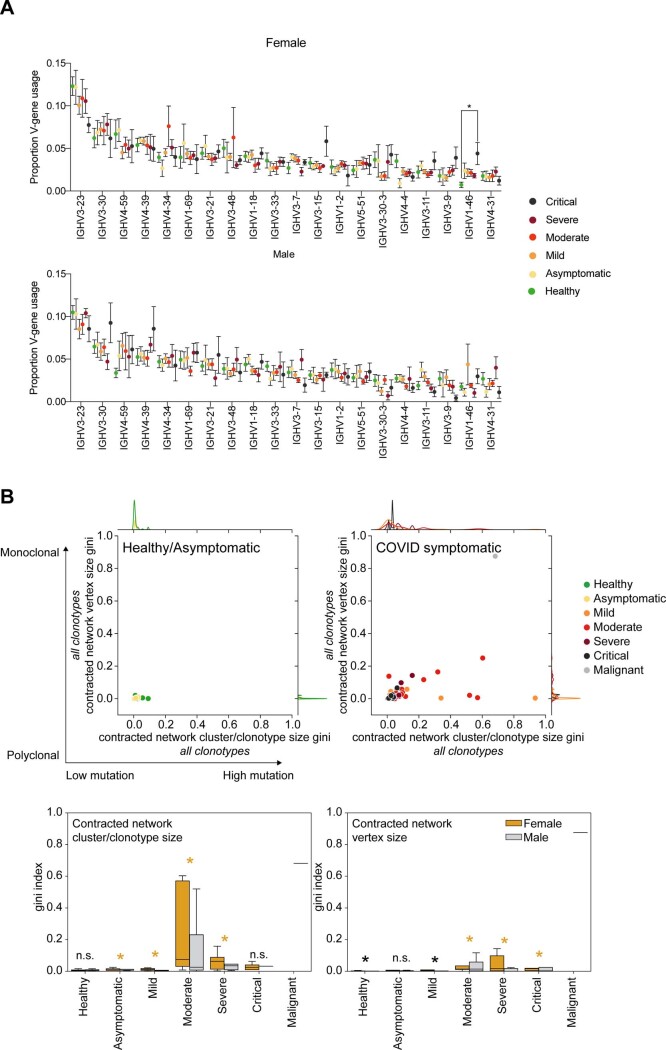
Single-cell BCR analysis between genders across COVID-19 severity. **a**, Summary plot for BCR IGHV gene usage split by gender (top: female; bottom: male). Each data point is presented as the mean gene usage proportion + /- standard error of the mean for samples within each sample group. Kruskal-Wallis test with Benjamini-Hochberg *P < 0.05 (adjusted p = 0.035) n = 12, 5, 8, 20, 6 and 6 biologically independent male patients/samples and n = 12, 4, 15, 10, 7 and 5 biologically independent female patients/samples for healthy, asymptomatic, mild, moderate, severe and critical respectively. **b**, (Top) Scatter plot of clonotype/cluster size by vertex size gini indices computed from contracted BCR networks (identical nodes are merged and counted). Each dot represents the gini indices of an individual coloured by severity status. Marginal histograms indicate the distribution of samples in a given severity status along the axes. (Bottom, left) Cluster/clonotype size (contracted network) gini indices separated by gender. (Bottom, right) Vertex size (contracted network) gini indices separated by gender. The boxes extend from the 25th to 75th percentiles. The whiskers go down to the smallest value and up to the largest. The line in the middle of the boxes is plotted at the median. Statistical tests were performed with non-parametric Mann-Whitney U test between the gender groups within each severity status and were considered statistically significant if Benjamini-Hochberg corrected *P* < 0.05 (denoted by *; n.s. denotes not significant). The Benjamini-Hochberg adjusted p-values are as follows: cluster/clonotype size (contracted network) gini indices comparisons: 3.42x10^-1^, 1.91x10^-25^, 7.71x10^-272^, 1.31x10^-169^, 3.84x10^-113^ and 2.98x10^-1^; vertex size (contracted network) gini indices comparisons: 1.12x10^-57^, 2.17x10^-1^, <1x10^-308^, 3.64x10^-49^, 2.69x10^-53^ and 2.37x10^-04^ - for healthy, asymptomatic, mild, moderate, severe and critical respectively. Colour of asterisks indicates which gender group displays a higher mean gini index (yellow: female; grey: male). n = 12, 5, 8, 20, 6 and 6 biologically independent male patients/samples and n = 12, 4, 15, 10, 7 and 5 biologically independent female patients/samples for healthy, asymptomatic, mild, moderate, severe and critical respectively. n = 1 for malignant (male).

We next assessed BCR clonality using *‘*dandelion’, a single-cell BCR-sequencing analysis package (Methods), and found substantially more clonal expansion in symptomatic individuals with COVID-19 (Fig. 4h and Extended Data Fig. 7). Expanded clonotypes were found across all major cell types with larger clonotypes present primarily in plasmablast/plasma cell clusters (Extended Data Fig. 8a,b). Within the expanded clonotypes, there was some evidence of class switching within symptomatic COVID-19 groups but not in the asymptomatic/healthy individuals (Extended Data Fig. 8c). Unlike other large-scale single-cell RNA-sequencing (scRNA-seq) studies with BCR profiling^15,24^, there was no obvious bias of immunoglobulin heavy-chain variable (*IGHV*) gene usage in general (Extended Data Fig. 9a). Disaggregating the *IGHV* gene usage data to individual gender groups showed that only *IGHV1-46* was significantly increased in women with critical COVID-19 relative to healthy controls (Extended Data Fig. 9a). Some related BCRs were present in different individuals, with more incidence of V and J gene usage and related amino acid sequences of heavy-chain and light-chain CDR3s observed in individuals with severe or critical disease, and in individuals from one center (Newcastle; Fig. 4i), which could arise due to local variants of the virus driving expansion of specific B cell clones. We note that none of these related BCRs were found to be expanded in the individuals, which was expected as only a relatively small number of B cells per individual were sampled. It would have been unlikely to find exactly matching heavy-chain and light-chain sequences across different individuals (even when allowing for somatic hypermutation variation), given the expected low coverage that arises from a small number of single cells (relative to bulk BCR sequencing). Finally, we observed disproportionate distribution in clonotype size, whether considering expanded or all clonotypes, and increased BCR mutations between men and women with COVID-19, with greater levels of both in women compared with men (Fig. 4j and Extended Data Fig. 9b). These differences in clonal expansion of B cells are consistent with a role in previous reports of worse outcomes in COVID-19 in men^39,40^.

**Fig. 5:**
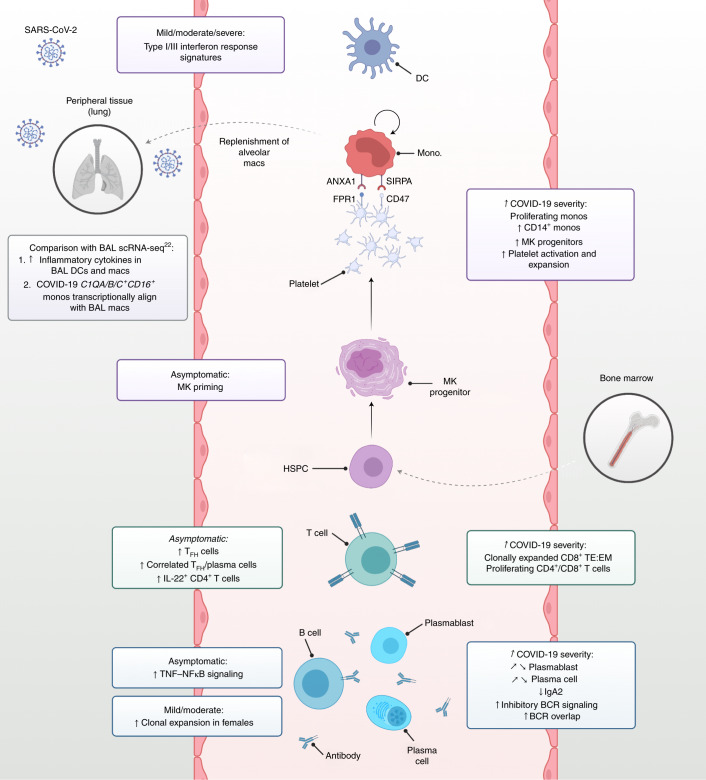
Integrated framework of the peripheral immune response in COVID-19. Schematic illustration of study highlights. Created with BioRender.com.

We summarize the immunological cellular and molecular profiles observed in our study distinguishing features between asymptomatic/mild disease from severe/critical disease (Fig. 5). Future longitudinal studies may enable us to distinguish if the distinct responses in asymptomatic and milder disease prevent progression to severe phenotypes.

## Discussion

Our cross-sectional multi-omics PBMC survey revealed several new insights into COVID-19 pathogenesis. Firstly, peripheral blood monocytes and DCs exhibit an interferon response to infection. We identified CD1QA/B/C^+^CD16^+^ monocytes, coexpressing receptors and ligands for interactions with platelets, that are predicted to replenish alveolar macrophages in COVID-19. Secondly, altered hematopoiesis is evident in the peripheral circulation with megakaryocyte-primed gene expression in the earliest CD34^+^CD38^+^ HSPCs, and expanded megakaryocyte progenitors in the response to COVID-19. We reveal a balance in protective versus immunopathogenic adaptive immune responses in COVID-19 patients. Previous studies have reported expanded proliferative CD4^+^ and CD8^+^ T cells with disease severity^24^, but a reduction in ɣδ T cells^24,41^, consistent with our study. In addition, we observed enrichment of T_H_1 cells in asymptomatic donors, consistent with previously reported IFNγ and IL-2 antigen-specific T cells in asymptomatic individuals^42^. We report expansion of CD8^+^ effector T cells, which likely include antigen-specific short-lived effector cells that could lead to uncontrolled inflammation and immunopathology, expanding on previous reports^43–46^.

The expansion of plasmablasts and plasma cells is less evident in critical and severe disease than in moderate and mild disease, in contrast to previous studies that reported the diminished plasmablast expansion in convalescent stages and not within active disease^15^. This response is paralleled by the T_FH_ profile in individuals with COVID-19 and is consistent with postmortem observations showing a lack of germinal centers in lymph nodes and spleen in individuals with fatal COVID-19 and a decrease in T_FH_ cells^44^. Our data revealed a significant decrease in IgA2 in symptomatic COVID-19 compared to asymptomatic donors, suggesting that maintenance of a robust mucosal humoral immune response may influence the fate of individuals infected with SARS-CoV-2. We observed a diminished IFNα response in the B cell compartments of individuals with critical and severe disease, further emphasizing a crucial role of these responses in outcomes, as previously reported in patients with COVID-19 who had type I IFN antibodies^47^. Our data also suggest differential BCR clonality and mutation frequencies between gender groups, which may contribute to the differing clinical outcomes observed between men and women with COVID-19^48^. Our study provides a valuable resource, exploitable for translational studies, and a template for future integrative meta-analysis of single-cell multi-omics datasets from individuals with COVID-19 worldwide.

## Methods

### Ethics and sample collection

#### Newcastle

Participants were recruited and consented under the Newcastle Biobank (Research Ethics Committee (REC) no. 17/NE/0361; Integrated Research Application System (IRAS) no. 233551) study and ethical governance. For the COVID-19-positive samples and healthy controls, peripheral blood was collected in EDTA tubes and serum separator tubes and processed within 4 h of collection.

For the IV-LPS control samples, ethical approval was granted by a REC (17/YH/0021). Healthy volunteers provided informed, written consent and passed the inclusion criteria as set out in the study protocol (Supplementary File 1.) LPS was obtained from Clinical Center Reference Endotoxin (94332B1, National Institutes of Health) and injected intravenously as a bolus dose of 2 ng kg^−1^. Blood samples were taken before IV-LPS administration (baseline), at 90 min, and at 10 h after challenge. Venous blood was drawn from an 18-gauge venous cannula and was collected into EDTA and serum separator tubes. Only samples from 90 min and 10 h were analyzed in this study.

#### Cambridge

Study participants were recruited between 31 March 2020 and 20 July 2020 from patients attending Addenbrooke’s Hospital with suspected COVID-19 or a confirmed diagnosis of COVID-19 by nucleic acid amplification test (including point-of-care testing), patients admitted to Royal Papworth Hospital NHS Foundation Trust or Cambridge and Peterborough Foundation Trust with a confirmed diagnosis of COVID-19, together with health-care workers identified through staff screening with a positive PCR test for SARS-CoV-2. Controls were recruited among hospital staff attending Addenbrooke’s serology screening program, and selected to cover the whole age spectrum of COVID-19-positive study participants, across gender groups. Only controls with negative serology results (45 of 47) were subsequently included in the study. Recruitment of inpatients at Addenbrooke’s Hospital and health-care workers was undertaken by the National Institute for Health Research (NIHR) Cambridge Clinical Research Facility outreach team and the NIHR BioResource research nurse team. Ethical approval was obtained from the East of England Cambridge Central Research Ethics Committee (NIHR BioResource, REC no. 17/EE/0025; ‘Genetic variation and altered leukocyte function in health and disease (GANDALF)’, REC no. 08/H0308/176). Informed consent was obtained from all participants. Each participant provided 27 ml of peripheral venous blood collected into a 9-ml sodium citrate tube.

#### London

Participants aged 18 years and older were recruited from two large hospital sites in London, United Kingdom, namely University College London (UCL) Hospitals NHS Foundation Trust and Royal Free London NHS Foundation Trust during the height of the pandemic in the United Kingdom (April to July 2020). All participants provided informed consent. Ethical approval was obtained through the Living Airway Biobank, administered through UCL Great Ormond Street Institute of Child Health (REC no. 19/NW/0171, IRAS project no. 261511), as well as by the local R&D departments at both hospitals. At daily virtual COVID-19 coordination meetings, suitable participants were chosen from a list of newly diagnosed and admitted patients within the preceding 24 h (based on a positive nasopharyngeal swab for SARS-CoV-2). Participants with typical clinical and radiological COVID-19 features but with a negative screening test for SARS-CoV-2 were excluded. Other exclusion criteria included active hematological malignancy or cancer, known immunodeficiencies, sepsis from any cause and a blood transfusion within 4 weeks. Maximal severity of COVID-19 was determined retrospectively by identifying the presence of symptoms, the need for oxygen supplementation and the level of respiratory support. Peripheral blood sampling was performed before inclusion to any pharmacological interventional trials.

Samples were collected and transferred to a category level 3 facility at UCL and processed within 2 h of sample collection. Peripheral blood was centrifuged after adding Ficoll-Paque Plus, and PBMCs, serum and neutrophils were separated, collected and frozen for later processing.

### Clinical status assignment

Clinical metadata were collected at the point-of-care sample collection, including current oxygen requirements and location. This was used to assign disease severity status. Participants based on a ward and not requiring oxygen were defined as having ‘mild’ disease. Participants outside of an intensive care unit (ICU) environment requiring oxygen were defined as having ‘moderate’ disease. All patients in ICU and/or requiring noninvasive ventilation were defined as having ‘severe’ disease. Participants requiring intubation and ventilation were defined as having ‘critical’ disease. There were no patients in ICU that did not require supplemental oxygen.

### PBMC isolation and dead cell removal

#### Newcastle

PBMCs were isolated from blood samples using Lymphoprep (StemCell Technologies) density gradient centrifugation according to the manufacturer’s instructions. Single-cell suspensions were then washed with Dulbecco’s PBS (Sigma) and frozen in aliquots containing 5–10 million cells in 90% (vol/vol) heat-inactivated FCS (Gibco) and 10% (vol/vol) DMSO (Sigma-Aldrich). On the day of the experiment, the cells were thawed for 1 min, transferred to wash buffer (PBS supplemented with 2% (vol/vol) FCS and 2 mM EDTA) and centrifuged at 500*g* for 5 min. Resuspended cells were passed through a 30-μm filter and counted before live-cell magnetic-activated cell sorting (MACS) enrichment with the dead cell removal kit (Miltenyi Biotech), per the manufacturer’s instructions. Cell pellets were resuspended in microbeads and incubated at room temperature for 15 min. Each stained sample was passed through an LS column and rinsed with binding buffer (all from Miltenyi Biotec) before centrifugation. Cell pellets were resuspended in wash buffer and counted for antibody staining by cellular indexing of transcriptomes and epitopes by sequencing (CITE-seq).

#### Cambridge

PBMCs were isolated using Leucosep tubes (Greiner Bio-One) with Histopaque 1077 (Sigma) by centrifugation at 800*g* for 15 min at room temperature. PBMCs at the interface were collected, rinsed twice with autoMACS running buffer (Miltenyi Biotech) and cryopreserved in FBS with 10% DMSO. All samples were processed within 4 h of collection. Purified PBMCs were thawed at 37 °C, transferred to a 50-ml tube, and ten volumes of prewarmed thawing medium (IMDM; Gibco, 12440-053) with 50% (vol/vol) FCS (not heat inactivated; PAN-Biotech, P40-37500) and 0.1 mg ml^−1^ DNase I (Worthington, LS002139)) were added slowly and dropwise, followed by centrifugation at 500*g* for 5 min. The pellet was resuspended in 1 ml of FACS buffer (PBS; Sigma, D8537) with 3% (vol/vol) heat-inactivated FCS, and the viability of each sample was assessed by counting in an improved Neubauer chamber using Trypan blue. Pools of four samples were generated by combining 0.5 million live cells per individual (2 million live cells in total). The pools were washed twice in FACS buffer (10 ml and 2 ml, respectively) followed by centrifugation for 5 min at 500*g*. The pellet was then resuspended in 35 μl of FACS buffer and the viability of each pool was assessed.

#### London

Peripheral whole blood was collected in EDTA tubes and processed fresh via Ficoll-Paque Plus separation (GE Healthcare, 17144002). The blood was first diluted with 5 ml 2 mM EDTA-PBS (Invitrogen, 1555785-038), before 10–20 ml of diluted blood was carefully layered onto 15 ml of Ficoll in a 50-ml falcon tube. If the sample volume was less than 5 ml, blood was diluted with an equal volume of EDTA-PBS and layered onto 3 ml Ficoll. The sample was centrifuged at 800*g* for 20 min at room temperature. The plasma layer was carefully removed and the PBMC layer collected using a sterile Pasteur pipette. The PBMC layer was washed with three volumes of EDTA-PBS by centrifugation at 500*g* for 10 min. The pellet was suspended in EDTA-PBS and centrifuged again at 300*g* for 5 min. The PBMC pellet was collected and the cell number and viability assessed using Trypan blue. Cell freezing medium (90% FBS and 10% DMSO) was added dropwise to PBMCs slowly on ice and the mixture cryopreserved at −80 °C until further full-sample processing.

### TotalSeq-C antibody staining and 10x Chromium loading

#### Newcastle

Approximately 200,000 cells from each donor were stained with Human TruStain FcX Fc Blocking Reagent (BioLegend, 422302) for 10 min at room temperature. The cells were then stained with the custom panel TotalSeq-C (BioLegend, 99813; Supplementary Table 1) for 30 min at 4 °C. Cells were then washed twice with PBS supplemented with 2% (vol/vol) FCS and 2 mM EDTA (Sigma) before resuspending in PBS and counting. Approximately 20,000–30,000 cells per sample were loaded onto the 10x Chromium controller using Chromium NextGEM Single Cell V(D)J Reagent kits v1.1 with Feature Barcoding technology for Cell-Surface Protein (10x Genomics) according to the manufacturer’s protocol.

#### Cambridge

Half a million viable cells were resuspended in 25 μl of FACS buffer and incubated with 2.5 μl of Human TruStain FcX Fc blocking reagent (BioLegend, 422302) for 10 min at 4 °C. The TotalSeq-C antibody cocktail (BioLegend 99813; Supplementary Table 1) was centrifuged at 14,000*g* at 4 °C for 1 min, resuspended in 52 μl of FACS buffer, incubated at room temperature for 5 min and centrifuged at 14,000*g* at 4 °C for 10 min. Around 25 μl of solution was subsequently added to each sample pool and incubated for 30 min at 4 °C in the dark. Pools were washed three times with 27 volumes (1.4 ml) of FACS buffer, followed by centrifugation at 500*g* for 5 min. The pellet was resuspended in 62.5 µl of 1× PBS + 0.04% BSA (Ambion, AM2616), filtered through a 40-μm cell strainer (Flowmi, H13680-0040), and viable cells of each sample pool were counted in an improved Neubauer chamber using Trypan blue. Around 50,000 live cells (up to a maximum of 60,000 total cells) for each pool were processed using Single Cell V(D)J 5′ version 1.1 (1000020) together with Single Cell 5′ Feature Barcode library kit (1000080), Single Cell V(D)J Enrichment Kit, human B cells (1000016) and Single Cell V(D)J Enrichment Kit, human T cells (10x Genomics, 1000005) according to the manufacturer’s protocols.

#### London

Frozen PBMC samples were thawed quickly in a water bath at 37 °C. Warm RPMI 1640 medium (20–30 ml) containing 10% FBS was added slowly to the cells before centrifuging at 300*g* for 5 min. The pellet was then washed with 5 ml RPMI 1640-FBS and centrifuged again (300*g* for 5 min). The PBMC pellet was collected and cell number and viability determined using Trypan blue. PBMCs from four different donors were then pooled together at equal numbers (1.25 × 10^5^ PBMCs from each donor) to make up 5.0 × 10^5^ cells in total. The remaining cells were used for DNA extraction (Qiagen, 69504). The pooled PBMCs were stained with TotalSeq-C antibodies (BioLegend, 99814) according to manufacturer’s instructions. After incubating with half a vial of TotalSeq-C for 30 min at 4 °C, PBMCs were washed three times by centrifugation at 500*g* for 5 min at 4 °C. PBMCs were counted again and processed immediately for 10x 5′ single-cell capture (Chromium Next GEM Single Cell V(D)J Reagent Kit v1.1 with Feature Barcoding technology for Cell-Surface Protein-Rev D protocol). Two lanes of 25,000 cells were loaded per pool on a 10x chip.

### Library preparation and sequencing

#### Newcastle and London

Gene expression, TCR-enriched and BCR-enriched libraries were prepared for each sample according to the manufacturer’s protocol (10x Genomics). Cell-surface protein libraries were subjected to double the manufacturer’s recommended primer concentration and seven to eight amplification cycles during the sample index PCR to reduce the likelihood of daisy chains forming. Libraries were pooled per participant using a ratio of 6:2:1:1 for gene expression, cell-surface protein, TCR-enriched and BCR-enriched libraries, respectively. All libraries were sequenced using a NovaSeq 6000 (Illumina) to achieve a minimum of 50,000 paired-end reads per cell for gene expression and 20,000 paired-end reads per cell for cell-surface protein, TCR-enriched and BCR-enriched libraries.

#### Cambridge

The samples were subjected to 12 cycles of cDNA amplification and 8 cycles for the cell-surface protein library construction. Following this, the libraries were processed according to the manufacturer’s protocol. Libraries were pooled per sample using a ratio of 9:2.4:1:0.6 for gene expression, cell-surface protein expression, and TCR and BCR enrichment libraries, respectively. Samples were sequenced using a NovaSeq 6000 (Illumina), using S1 flow cells.

### Alignment and quantification

Droplet libraries were processed using Cell Ranger v4.0. Reads were aligned to the GRCh38 human genome concatenated to the SARS-CoV-2 genome (NCBI SARS-CoV-2 isolate Wuhan-Hu-1) using STAR^49^ (v2.5.1b) and unique molecular identifiers (UMIs) deduplicated. CITE-seq UMIs were counted for GEX and ADT libraries simultaneously to generate feature-X droplet UMI count matrices.

### Doublet identification

#### Newcastle

Scrublet (v0.2.1) was applied to each sample to generate a doublet score. These formed a bimodal distribution so the tool’s automatic threshold was applied.

#### Cambridge

Non-empty droplets were called within each multiplexed pool of donors using the emptyDrops function implemented in the Bioconductor package DropletUtils (v1.10.3), using a UMI threshold of 100 and FDR of 1%. The probability of being a doublet was estimated for each cell per sample (that is, one 10x lane) using the ‘doubletCells’ function in ‘scran’ based on highly variable genes (HVGs). Next, we used ‘cluster_walktrap’ on the shared nearest neighbor graph that was computed on HVGs to form highly resolved clusters per sample. Per-sample clusters with either a median doublet score greater than the median + 2.5× median absolute deviation (MAD) or clusters containing more than the median + 2.5× MAD genotype doublets were tagged as doublets. This was followed by a second round of highly resolved clustering across the whole dataset, in which again cells belonging to clusters with a high proportion (>60%) of cells previously labeled as doublets were also defined as doublets.

#### London

For pooled donor CITE-seq samples, the donor ID of each cell was determined by genotype-based demultiplexing using souporcell (v2)^50^. Souporcell analyses were performed with ‘skip_remap’ enabled and a set of known donor genotypes given under the ‘common_variants’ parameter. The donor ID of each souporcell genotype cluster was annotated by comparing each souporcell genotype to the set of known genotypes. Droplets that contained more than one genotype according to souporcell were flagged as ‘ground-truth’ doublets for heterotypic doublet identification. Ground-truth doublets were used by DoubletFinder (v2.0.3)^51^ to empirically determine an optimal ‘p*K*’ value for doublet detection. DoubletFinder analysis was performed on each sample separately using ten principal components (PCs), a ‘p*N*’ value of 0.25, and the ‘nExp’ parameter estimated from the fraction of ground-truth doublets and the number of pooled donors.

### CITE-seq background signal removal

Background antibody-specific and nonspecific staining was subtracted from ADT counts in each data set from the 3 data acquisition sites separately. Antibody-derived tag (ADT) counts for each protein were first normalized using counts per million and log transformed, with a pseudocount of +1. To estimate the background signal for each protein, a two-component Gaussian mixture model, implemented in the R package function ‘mclust’ (v5.4.7), was fit across the droplets with a total UMI count of >10 and <100 from each experimental sample separately. The mean of the first Gaussian mixture model component for each protein was then subtracted from the log counts per million from the QC-passed droplets in the respective experimental sample.

### Quality control, normalization, embedding and clustering

Combined raw data from the three centers was filtered to remove cells that expressed fewer than 200 genes and >10% mitochondrial reads. Data were normalized (scanpy: normalize_total), log + 1 corrected (scanpy: log1p) and HVGs identified using the Seurat vst algorithm (scanpy: highly_variable_genes). Harmony was used to adjust PCs by sample ID and used to generate the neighborhood graph and embedded using UMAP. Clustering was performed using the Leiden algorithm with an initial resolution of 3. For initial clustering, differentially expressed genes were calculated using the Wilcoxon rank-sum test.

### Cluster differential abundance testing

Numbers of cells of each cell subtype were quantified in each participant and control sample (donors) to compute a matrix of cell type × donor counts. Cell type abundance counts were modeled as a function of either disease severity (as an ordinal variable: healthy < asymptomatic < mild < moderate < severe < critical) or days from symptom onset, adjusting for age, gender, batch and days from onset, in a negative binomial generalized linear model (NB GLM), implemented in the Bioconductor package edgeR. Counts were normalized in the model using the (log) of the total numbers of all cells captured for each donor. Hypothesis testing was performed using a quasi-likelihood *F*-test for either a linear or a quadratic trend across disease severity groups (asymptomatic > mild > moderate > severe > critical), or comparing healthy controls to SARS-CoV-2-infected donors (healthy versus all asymptomatic, mild, moderate, severe and critical groups). Differentially abundant cell types were determined using a 10% FDR. Due to compositional differences across sites, when analyzing differential abundance of myeloid populations (Fig. 2), only samples from Newcastle and London were included.

### Relative importance of metadata on cell type composition

The number of cells for each sample (*N* = 110 samples in total with complete metadata) and cell type (18 different cell types in total) combination was modeled with a generalized linear mixed model with a Poisson outcome. The five clinical factors (COVID-19 swab result, age, gender, disease severity at day 0 and days from onset) and the two technical factors (patient and sequencing center) were fitted as random effects to overcome the collinearity among the factors. The effect of each clinical/technical factor on cell type composition was estimated by the interaction term with the cell type. The likelihood ratio test was performed to assess the statistical significance of each factor on cell type abundance by removing one interaction term from the full model at a time. The number of factors was used to adjust multiple testing with the Bonferroni approach. The ‘glmer’ function in the lme4 package implemented on R was used to fit the model. The standard error of variance parameter for each factor was estimated using the ‘numDeriv’ package.

### Cydar analysis

We utilized cydar to identify changes in cell composition across the different severity groups based on the protein data alone. First, the background-corrected protein counts from the three different sites were integrated using the ‘fastMNN’ method (k = 20, d = 50, cos.norm = TRUE) in batchelor (v1.6.2). The batch-corrected counts for 188 proteins (four rat/mouse antibody isotypes were removed) were then used to construct hyperspheres using the ‘countCells’ function (downsample = 7) with the tolerance parameter chosen so that each hypersphere had at least 20 cells, estimated using the ‘neighborDistances’ function. To assess whether the abundance of cells in each hypersphere were associated with disease status, hypersphere counts were analyzed using the quasi-likelihood method in edgeR (v3.32.1). After filtering out hyperspheres with an average count per sample below five, we fitted a mean-dependent trend to the NB dispersion estimates. The trended dispersion for each hypersphere was used to fit an NB GLM using the log-transformed total number of cells as the offset for each sample and blocking for gender, age and batch. The quasi-likelihood *F*-test was used to compute *P* values for each hypersphere, which were corrected for multiple testing using the spatial FDR method in cydar.

### Comparisons of PBMC annotation using the Azimuth tool

The final annotation of PBMCs was compared to a published PBMC annotation using the Azimuth tool (http://azimuth.satijalab.org/app/azimuth/). Because of size restrictions of 100,000 cells, our data were subsampled to 10% of the total cells. After running the algorithm, results with a prediction score < 0.5 were removed (5.8% of total removed). For each cluster in the COVID-19 PBMC data, the percentage of cells mapped to each cluster in the Azimuth annotation was calculated.

### Interferon, TNF and JAK–STAT response scoring

A list of genes related to response to type I interferons was obtained from the GSEA Molecular Signatures Database (MSigDB; GO:0034340). Enrichment of the interferon score was measured using the ‘tl.score_genes’ tool in ‘scanpy’, which subtracts the average expression of all genes in the dataset from the average expression of the genes in this list. The scores were averaged across clusters and clinical status and expressed as a fold change over the interferon score in the equivalent healthy cluster.

### kBET analysis

The kBET^52^ algorithm (https://github.com/theislab/kBET/) was run for each cluster (Fig. 1) using the UMAP coordinates generated from Harmony-adjusted PCs and the sample number as the batch factor. The same procedure was then performed with the same annotation but using the UMAP coordinates generated from non-Harmony-adjusted PCs. The resultant rejection rates were averaged across clusters and compared using a Wilcoxon paired signed-rank test.

### Bronchoalveolar lavage data analysis

scRNA-seq data from BAL was obtained from the Gene Expression Omnibus (accession no. GSE145926)^22^. Raw data were analyzed using the same pipeline as for PBMC data, specifically using the same QC cutoffs (minimum of 200 genes and <10% mitochondrial reads per cell), and data were batch corrected using Harmony by donor ID. To gain greater resolution of MPs, the DCs and macrophages were analyzed with further rounds of subclustering to identify DC1, DC2 and mature DC subsets.

### PAGA analysis of blood monocytes and BAL macrophages

Annotated raw expression datasets of BAL macrophages and COVID-19 PBMCs were merged and data log normalized and scaled as for the original datasets. The top 3,000 HVGs were chosen using the Seurat ‘vst’ method and used for downstream analysis. PCs were batch corrected by donor and used to build a neighborhood graph. The PAGA tool in scanpy (tl.paga) was used to generate the abstracted graph between clusters.

### CellphoneDB

CellphoneDB^53^ was used to assess putative interactions between monocytes (CD14_mono, CD83_CD14_mono, C1_CD16_mono, CD16_mono and Prolif_mono) and platelets. The tool was run for 100 iterations, and an expression threshold of 0.25 (limiting the analysis to genes expressed by 25% of cells). For downstream analysis, we focused on interactions between platelets and any monocyte subset.

### Platelet activation

Differentially expressed genes in platelets between healthy control and COVID-19 samples were filtered for those predicted to be involved in platelet activation (https://www.gsea-msigdb.org/gsea/msigdb/cards/REACTOME_PLATELET_ACTIVATION_SIGNALING_AND_AGGREGATION).

### HSPC commitment scoring

HSPCs were subsetted from the data and Leiden clusters generated using the same pipeline and parameters as for the whole PBMC dataset. Differentially expressed genes between the HSPC clusters that showed evidence of lineage commitment (megakaryocyte, erythroid and myeloid) were calculated using the FindAllMarkers tool in Seurat (with thresholds of genes expressed by 25% of cells and with a log fold change of 0.25), and genes with an adjusted *P*-value cutoff of 0.05 were used to generate gene signatures for each. Enrichment scores of these signatures in the CD38-negative and CD38-positive HSPC clusters were calculated using the tl.score_genes tool in scanpy (v1.6.0). The average expression of these enrichment scores in the CD38-negative and CD38-positive HSPC clusters was averaged by donor then compared across clinical states. Differences between groups were assessed using ANOVA with pairwise comparisons using Tukey’s test.

### Multiplex cytokine analysis

Serum was obtained from peripheral blood in red-topped serum Vacutainers (BD, 367815) and allowed to clot for at least 30 min before centrifugation (800*g* for 10 min) to separate the serum. After collection, serum was frozen at −80 °C and thawed on ice on the day of experiment. The assay was carried out using the Cytokine/Chemokine/Growth Factor 45-Plex Human ProcartaPlex Panel 1 kit (Invitrogen, EPX450-12171-901), utilizing the Luminex xMAP technology and according to the manufacturer’s protocol. Each sample was run in duplicate. The values of each analyte were detected using the MAGPIX system and analyzed using the ProcartaPlex Analyst v1.0 Software (Thermo Fisher Scientific).

### Restimulation of PBMCs with SARS-Cov-2 peptide S

Purified PMBCs were thawed at 37 °C, transferred into a 15-ml tube with 10 ml prewarmed complete culture media RPMI 1640 medium (Sigma-Aldrich, R0883) supplemented with 10% (vol/vol) FCS (Gibco, 10270-106) and 1% (vol/vol) penicillin–streptomycin (100 U ml^−1^ and 100 μg ml^−1^, respectively; Sigma-Aldrich, P0781) and 1% (vol/vol) l-glutamine (2 mM; Sigma-Aldrich, G7513), referred to as RPMI 10, followed by centrifugation at 500*g* for 5 min. Cell pellet was resuspended in 500 μl RPMI 10 with added DNase (1 μg ml^−1^; Merck, 10104159001), divided into five wells of a round-bottom 96-well plate and left to rest at 37 °C for 1 h. Cells were stimulated with SARS-CoV-2 PepTivator peptide S for pan-HLA (2 μg ml^−1^; Miltenyi Biotec, 136-126-700) and PMA/ionomycin as a control (2 μl ml^−1^ Cell Activation cocktail; BioLegend, 423301), and incubated at 37 °C for 2 h. Negative controls were left untreated. Brefeldin A (2 μg ml^−1^; GolgiPlug, BD Bioscience, 555029) and anti-CD107a-BB700 (1:50 dilution; clone H4A3; BD Bioscience, 566558) was added for an additional 4 h into all conditions. Cells were stained for detection of activation-induced markers and intracellular cytokines 6 h after stimulation and subjected to flow cytometry.

### Flow cytometry of stimulated cells

PBMCs stimulated for 6 h with the SARS-CoV-2 peptide were washed with PBS, and cell-surface stained for 1 h at room temperature: anti-CD14-FITC (1:50 dilution; clone M5E2; BD Biosciences, 555397), anti-CD19-FITC (1:50 dilution; clone 4G7; BD Biosciences, 345776), anti-CD137-PE-Dazzle594 (1:50 dilution; clone 4B4-1; BioLegend, 309826), anti-CCR7-PE-Cy7 (1:50 dilution; clone G043H7; BioLegend, 353226), anti-CD45RO-APC-H7 (1:50 dilution; clone UCHL1; BD Biosciences, 561137), anti-CD28-BV480 (1:50 dilution; clone CD28.2; BD Biosciences, 566110), anti-CD4-BV785 (1:100 dilution; clone SK3; BioLegend, 344642), anti-CD3-BUV395 (1:50 dilution; clone UCHT1; BD Biosciences, 563546), anti-CD8-BUV496 (1:100 dilution; clone RPA-T8; BD Biosciences, 564804), anti-CD25-BUV737 (1:100 dilution; clone 2A3; BD Biosciences, 612806) and viability dye Zombie Yellow (1:200 dilution; BioLegend, 423104). Cells were washed with PBS 2% (vol/vol) FCS, fixed with 4% (wt/vol) paraformaldehyde (Thermo Fisher Scientific, 28908) and kept at 4 °C overnight. Subsequently, cells were washed with PBS, permeabilized with Perm/Wash buffer (BD Biosciences, 554723) according to the manufacturer’s instructions, and stained with intracellular antibodies for 1 h on ice: anti-IL10-PE (1:10 dilution; clone JES3-19F1; BD Biosciences, 559330), anti-IFN-APC (1:25 dilution; Miltenyi Biotec, 130-090-762), anti-TNF-AF700 (1:50 dilution; clone MAb11; BioLegend, 502928), anti-IL-2-BV421 (1:100 dilution; clone 5344.111; BD Biosciences, 562914) and anti-CD154-BV605 (1:50 dilution; clone 24-31; BioLegend, 310826). Cells were washed, transferred to flow cytometry 5-ml tubes, and acquired on a Symphony A5 flow cytometer (BD Biosciences). Data were analyzed by FlowJo v10 (BD Biosciences).

### GSEA analysis

Preranked gene-set analysis on MSigDB v7.2 Hallmark gene sets^54^ was performed using preranked gene lists with the fgsea^55^ package in R. Genes were preranked according to signed −log_10_ *P* values for all preranked gene-set analysis procedures. For B cells, generation of the rank gene list was performed using a Wilcoxon rank-sum test (via ‘tl.rank_genes_groups’ in scanpy) with each day 0 COVID-19 statuses (asymptomatic to symptomatic critical) as the ‘tests’ versus day 0 healthy samples as the ‘reference/controls’.

### T cell clustering, annotation and visualization

Droplets labeled as T cells (CD4, CD8, T_reg_, MAIT and γδ T) were subset from those in Fig. 1b and reclustered using a set of HVGs calculated within each batch, the union of which was used to estimate the first 50 PCs across cells using the ‘irbla’ R package (v2.3.3). Batch effects were removed across the first 30 PCs using the fastMNN^56^ implementation in the Bioconductor package batchelor (v1.6.2; *k* = 50). A *k*-nearest-neighbor graph (*k* = 20) was computed across these 30 batch-integrated PCs using the ‘buildKNNGraph’ function implemented in the Bioconductor package scran (v1.18.3), which was then used to group cells into connected communities using Louvain^57^ clustering implemented in the R package ‘igraph’ (v1.2.6). Clusters that displayed mixed profiles of T and other lymphoid lineages, that is, CD19, CD20 and immunoglobulin genes, were classed as doublets and removed from downstream analyses. Clusters indicative of NK cells (CD3^−^CD56^+^) were subsequently annotated as such and removed from T cell analyses. Remaining clusters were annotated using a combination of canonical protein and mRNA markers for major αβ T cells (CD4, CD8, CCR7, CD45RA, CD45RO, CD62L, CD27, CD38, CD44, CXCR5, CD40LG, *CCR7*, *FOXP3* and *IKZF2*), γδ T cells (Vγ9, Vγ2, *TRGV9* and*TRDV2*) and invariant T cells, MAIT (Vα24-Jα18 and *TRAV1.2*) and NK T (CD3, CD16, CD56, *NCAM1*, *NCR1* and *FCGR3A*) cells. Polarized CD4^+^ T cell annotations were refined using the combination of transcription factor genes and expressed cytokines for the respective T_H_ cell types: T_H_1 (*IFNG*, *TBX21* and *TNFA*), T_H_2 (*GATA3*, *IL4* and *IL5*) and T_H_17 (*RORC*, *IL17A*, *IL17F* and *IL21*). Where clusters appeared heterogeneous in their expression of T cell lineage markers, single-cell annotations were refined based on the coexpression of specific marker gene and protein pairs. Dot plots to visualize marker protein and mRNA expression across clusters were generated using the R package ‘ggplot2’ (v3.3.3). UMAP^58^ was used to project all single T cells into a two-dimensional space (*k* = 31) using the first 30 batch-integrated PCs as input with the R package ‘umap’. R v4.0.3 and Bioconductor v3.12 were used for all analyses.

### T cell differential gene expression analysis

Differential gene expression analysis was performed across COVID-19 disease severity groups, ordered from healthy > asymptomatic > mild > moderate > severe > critical. Donor pseudo-bulk samples were first created by aggregating gene counts for each annotated T cell type, within each donor, where there were at least 20 cells of that type. Genes with fewer than three counts in any given pseudo-bulk sample, or fewer than five counts in total across donor pseudo-bulk samples, were removed before analysis. Differential gene expression testing was performed using an NB GLM implemented in the Bioconductor package edgeR^59,60^ (v3.32.1). Statistically significant differentially expressed genes were defined with FDR < 0.1. Functional annotation enrichment was performed using the Bioconductor package enrichR^61^(v3.0). Upregulated and downregulated differentially expressed genes in each T cell type were used as input, testing separately against the MSigDB Hallmark 2020 and Transcription Factor Protein–Protein Interactions gene sets. Significant enrichments were defined with 1% FDR.

### T cell receptor analysis

Single-cell TCRs were computed from the TCR-sequencing data using Cell Ranger v4.0.0. The unfiltered outputs of reconstructed TCR contigs across all three sites (Newcastle, Cambridge and London) were combined before filtering using: (1) full-length CDR3, (2) each cell droplet barcode matched a TCR droplet barcode and (3) productive CDR3 spanning V + J genes. Chain-specific TCR clones were defined for each observed α-chain and β-chain by first concatenating the V, J and identical CDR3 nucleotide sequences. For each single T cell, these chains were then combined to form a single clonotype, removing cells that contained: (1) greater than two β-chains and greater than two α-chains and (2) a single α-chain or a single β-chain only. T cells with exactly two β-chains and one α-chain, or those with exactly two α-chains and one β-chain were retained. TCR clonotypes were counted within each donor sample, and expanded clones were defined where more than one cell was assigned to the TCR clonotype.

The proportion of expanded clones as a function of a linear trend across disease severity groups was modeled using logistic regression, adjusted for age, gender and batch. A separate model was run for each T cell subtype that contained at least five cells assigned to the expanded TCR clonotypes. Linear trend *P* values were corrected for multiple testing using the Benjamini–Hochberg procedure^62^.

The TE:EM ratio was calculated within each donor, using the number of observed expanded clonotypes. The TE:EM ratio change across COVID-19 severity was tested using a robust linear model implemented in the R package ‘robustbase’ (v0.93-7), regressing the TE:EM ratio on either disease severity as an ordered linear variable (asymptomatic > mild > moderate > severe > critical) or symptom duration, adjusted for age, gender and batch. Statistical significance was defined based on the linear trend across disease severity (*P* ≤ 0.01). An equivalent analysis was performed, restricted to participants with a shorter symptom duration (≤24 d).

### Differential correlation analysis

Changes in the correlations between PBMC cell types were computed using a differential correlation analysis, implemented in the R package *DCARS*^63^ (v0.3.5). Cell type proportions were computed by normalizing the counts of each cell type within each donor by the total number of cells captured for that donor sample. Donor samples were ranked according to their disease severity (healthy > asymptomatic > mild > moderate > severe > critical). Differential correlation analysis was then performed between CD4.T_FH_ versus all B cell types. Statistically significant differentially correlated cell types were defined with empirical *P* value ≤ 0.1, estimated from 10,000 permutations.

### BCR V(D)J analysis

Single-cell V(D)J data from the 5′ Chromium 10x kit were initially processed with Cell Ranger vdj pipeline (4.0.0). BCR contigs contained in ‘filtered_contigs.fasta’ and ‘filtered_contig_annotations.csv’ from all three sites were then preprocessed using ‘immcantation’ inspired preprocessing pipeline^64^ implemented in the dandelion Python package; dandelion is a new single-cell BCR-seq analysis package for 10x Chromium 5′ data. All steps outlined below were performed using dandelion v0.0.27.post2 and are available at https://github.com/clatworthylab/dandelion/.

### BCR preprocessing

Individual BCR contigs were reannotated with ‘igblastn’ v1.1.15 using the IMGT reference database (downloaded on 30 June 2020)^65^ by calling changeo’s ‘AssignGenes.py’ script, and reannotated contigs in ‘blast’ format were parsed into the Adaptive Immune Receptor Repertoire standards 1.3 format with changeo’s ‘MakeDB.py’ script. Amino acid sequence alignment information not present in the output from blast format was retrieved from reannotation with igblastn in AIRR format. Heavy-chain V-gene alleles were corrected for individual genotypes with TIgGER^66^ (v1.0.0) using a modified ‘tigger-genotype.R’ script from ‘immcantation’ suite. Germline sequences were reconstructed based on the genotype corrected V-gene assignments using changeo’s (v1.0.1) ‘CreateGermines.py’ script; contigs which failed germline sequence reconstruction were removed from further analysis. Constant genes were reannotated using blastn (v2.10.0+) with CH1 regions of constant gene sequences from IMGT followed by pairwise alignment against curated sequences to correct assignment errors due to insufficient length of constant regions.

### BCR filtering

Contigs assigned to cells that passed QC on the transcriptome data were retained for further QC assessment, which included the following checks: (1) contigs with mismatched locus and V, J and constant gene assignments were removed from the analysis; (2) cell barcodes with multiple heavy-chain contigs were flagged for filtering (exceptions to this were (a) when the multiple heavy-chain contigs were assessed to have identical V(D)J sequences but assigned as different contigs belonging to the same cell by Cell Ranger vdj pipeline, (b) when there was a clear dominance (assessed by difference in UMI count) by a particular contig, and (c) if and when there was presence of one IgM and one IgD contig assigned to a single cell barcode; in the first two cases, the contig with the highest UMI count was retained); (3) cell barcodes with multiple light-chain contigs were flagged for filtering; and (4) in situations where cell barcodes were matched with only light-chain contigs, the contigs were dropped from the V(D)J data but the transcriptome barcode was retained.

### B cell clone/clonotype definition

BCRs were grouped into clones/clonotypes based on the following sequential criteria that apply to both heavy-chain and light-chain contigs: (1) identical V and J gene usage, (2) identical junctional CDR3 amino acid length, and (3) at least 85% amino acid sequence similarity at the CDR3 junction (based on hamming distance). Light-chain pairing was performed using the same criteria within each heavy-chain clone. Only samples collected at day 0 of the study were analyzed from this step onwards and clones/clonotypes were called across the entire dataset; the sample from one of the donors who was subsequently found to have a B cell malignancy was separated from the analysis and processed independently.

### B cell clone/clonotype network

Single-cell BCR networks were constructed using adjacency matrices computed from pairwise Levenshtein distance of the full amino acid sequence alignment for BCR(s) contained in every pair of cells within each disease severity cohort. Construction of the Levenshtein distance matrices were performed separately for heavy-chain and light-chain contigs, and the sum of the total edit distance across all layers/matrices was used as the final adjacency matrix. To construct the BCR neighborhood graph, a minimum-spanning tree was constructed on the adjacency matrix for each clone/clonotype, creating a simple graph with edges indicating the shortest edit distance between a B cell and its nearest neighbor. Cells with identical BCRs, that is, cells with a total pairwise edit distance of zero, were then connected to the graph to recover edges trimmed off during the minimum-spanning-tree construction step. Fruchterman–Reingold graph layout was generated using a modified method to prevent singletons from flying out to infinity in ‘networkx’ (v2.5). Visualization of the resulting single-cell BCR network was achieved via transfer of the graph to relevant ‘anndata’ slots, allowing for access to plotting tools in scanpy.

The use of the BCR network properties for computing gini indices was inspired from bulk BCR-sequencing network analysis methods where distributions of clone sizes and vertex sizes (sum of identical BCR reads) in BCR clone networks were used to infer the relationships between BCR clonality, somatic hypermutation and diversity^67^. However, there are challenges with native implementation of this approach for single-cell data. Firstly, to enable calculation of network-based clone/cluster and vertex/node size distribution, BCR networks needed to be reduced such that nodes/cells with identical BCRs had to be merged and counted; this required the reconstruction of BCR networks per sample and discarding single-cell-level information. Furthermore, the process of node contraction and counting of merging events requires substantial computation time and resource. Secondly, this approach is dependent on sufficient coverage of the BCR repertoire, as the BCRs from the number of cells sampled (after QC) may not necessarily recapitulate the entire repertoire, which may underrepresent or overrepresent merged counts for gini index calculation. We propose the use of node closeness centrality computed on each expanded clone (clone size > 1) as an alternative metric to emulate the statistics to adapt to the single-cell nature of the data; closeness centrality defines how close and central each node is with respect to other nodes in the graph; therefore, cells with identical BCRs will have high closeness centrality scores, due to the way the BCR network is constructed in dandelion. Thus, we can quickly calculate if cells across clones, and/or samples overall, in the entire graph display proportionately/disproportionately high or low closeness centrality scores. One caveat to the current implementation is that it is only meaningful if there are clonotypes with at least two cells, as scores will only be computed for non-singleton components of the graph. Gini indices are computed using ‘skbio.diversity.alpha.gini_index’ (scikit-bio v0.5.6) with the ‘trapezoids’ method after clone definition and network generation. Summary visualization was performed using plotting tools in ‘seaborn’ (v0.11.0).

### Definition of BCR convergence across participants

BCR overlap was determined by collapsing sharing incidence of V and J gene usage and CDR3 amino acid sequences, in both heavy and light chains, between individuals into a binarized format (1 or 0). The information is turned into an adjacency matrix where an edge is created between two individuals if there is at least one clonotype (at least one cell from each individual displays an identical combination of heavy-chain and light-chain V and J gene usage with allowance for somatic hypermutation at the CDR3 junctional region) that is similar between the two individuals. Visualization was achieved using the ‘CircosPlot’ function from ‘nxviz’ package (v0.6.2).

### Reporting Summary

Further information on research design is available in the Nature Research Reporting Summary linked to this article.

## Online content

Any methods, additional references, Nature Research reporting summaries, source data, extended data, supplementary information, acknowledgements, peer review information; details of author contributions and competing interests; and statements of data and code availability are available at 10.1038/s41591-021-01329-2.

## Supplementary information

Supplementary Information Supplementary File 1 and 2 and Supplementary Fig. 1

Reporting Summary

Supplementary Tables 1–10 Supplementary Table 1: CITE-seq panel. List of TotalSeq-C antibodies, including clone and barcode. Supplementary Table 2: patient metadata. Status summary is based on the WHO COVID-19 classification (WHO/2019-nCoV/clinical/2020.5; https://www.who.int/publications/i/item/clinical-management-of-covid-19/). NA, not applicable. Not-known listed where information was unavailable. O_2_, supplemental oxygen via nasal cannulae, face mask or non-rebreather mask. NIV, noninvasive ventilation under continuous (CPAP) or bilevel (BiPAP) positive airway pressure. Supplementary Table 3: number of cells and donors contributing to each cell cluster. Supplementary Table 4: clinical whole-blood counts for Newcastle samples. Number of cells × 10^9^ per liter of blood. WBC, white blood cells. Supplementary Table 5: concentration in pg ml^−1^ of 45 analytes measured in serum. ≤0, below the limit of detection; asterisks indicate anti-inflammatory cytokines. Supplementary Table 6: type I/III interferon, JAK–STAT and TNF response enrichment scores for MP populations shown in Fig. 2f. Supplementary Table 7: statistical analysis values for Fig. 2f. Supplementary Table 8: statistical analysis values for Fig. 2k and Extended Data 3f. Supplementary Table 9: T cell clonality logistic regression model results across disease severity groups. *P* values calculated from a two-sided *t*-test, with Benjamini–Hochberg FDR correction Supplementary Table 10: T cell clonality logistic regression model results across symptom duration. *P* values calculated from a two-sided *t*-test, with Benjamini–Hochberg FDR correction.

## Data Availability

The dataset from our study can be explored interactively through the web portal https://covid19cellatlas.org/. The data object, as a h5ad file, can also be downloaded from https://covid19cellatlas.org/. The processed data are available to download from Array Express under accession number E-MTAB-10026. Source data are provided with this paper.

## Source data

Source Data Extended Data Fig. 5 Cell percentages for flow cytometry.

## Code availability

All data analysis scripts are available on https://github.com/scCOVID-19/COVIDPBMC/.
