# Supplementary material for: Single-cell multi-omics analysis of the immune response in COVID-19
Source: Nat Med. 2021 Apr 20;27(5):904–16. doi: 10.1038/s41591-021-01329-2 (PMC8121667; doi:10.1038/s41591-021-01329-2)
Supplement: Supplementary file 1 — and 2 and Supplementary Fig. 1 [file 41591_2021_1329_MOESM1_ESM.pdf]

nature portfolio

https://doi.org/10.1038/s41591-021-01329-2

Supplementary information

Single-cell multi-omics analysis of the immune response in COVID-19

In the format provided by the authors and unedited

Study Protocol

Does mitochondrial haplogroup predict the inflammatory response in a model of human endotoxaemia? (Does the DNA of our cell's batteries influence our response to bacteria?)

Chief Investigator	Prof John Simpson
Investigators	Dr Anthony Rostron<br>Dr Alistair Roy<br>Prof Muzlifah Haniffa<br>Dr John Widdrington<br>Dr Gavin Hudson<br>Dr Angela Pyle<br>Mr Jonathan Scott<br>Dr Marie-Helene Ruchaud-Sparagano<br>Dr Kathryn Musgrave<br>Julie Furneval<br>Ashley Allan<br>Dr Sarah Wiscombe<br>Dr Patrick Kesteven<br>Prof Patrick Chinnery<br>Prof Paul Corris
Funder	Newcastle University
Sponsor	Newcastle upon Tyne Hospitals NHS Foundation Trust
Protocol	Human endotoxaemia model v1.0 (29/07/2016)

Contents

	Page number
Contents	2
Funder and Sponsor Information	3
Funder contact details	3
Sponsor contact details	3
Research Team and Contacts	3
Chief Investigator	3
Members of the research team	4
Introduction	5
Experimental Plan using the Human Endotoxaemia Model	6
Objectives	7
Primary Objective	7
Secondary Objectives	8
Study Design	8
Participant Enrolment and Selection	8
Screening Visit	8
Setting	9
Study population	9
Inclusion criteria	9
Exclusion criteria	9
Consent	10
Ineligible and non-recruited participants	10
Study visits	10
Study Assessments	11
Data Analysis	11
Risk Assessments	11
Adverse Events	13
Discharge Criteria	14
End of study	15
Process if new information becomes available	15
Criteria for terminating the project	15
References	15

Funder and Sponsor Information

Funder contact details

Ms Amanda Topping
Institute of Cellular Medicine
Newcastle University
Framlington Place
Newcastle upon Tyne
NE2 4HH

Sponsor Information

Mrs Susan Ridge
Joint Research Office
Level 6 Leazes Wing
Royal Victoria Infirmary
Queen Victoria Road
Newcastle upon Tyne
NE1 4LP

Tel: 0191 2825213
Email: amanda.tortice@nuth.nhs.uk

Research Team and Contacts

Chief Investigator

Prof John Simpson
Professor of Respiratory Medicine
Institute of Cellular Medicine, 3rd Floor, William Leech Building
Medical School
Framlington Place
Newcastle upon Tyne
NE2 4HH

Tel: 0191 2227770
Fax: 0191 2220723
Email: j.simpson@ncl.ac.uk

Members of the research team

Dr Anthony Rostron, Academic Clinical Lecturer in Intensive Care Medicine, Newcastle University, Newcastle upon Tyne

Dr Alistair Roy, Consultant in Anaesthesia and Intensive Care Medicine, City Hospitals Sunderland Foundation Trust, Sunderland

Prof Muzlifah Haniffa, Wellcome Trust Senior Research Fellow in Clinical Science, Lister Institute Prize Fellow, Senior Lecturer and Honorary Consultant Dermatologist, Institute of Cellular Medicine, Newcastle University, Newcastle upon Tyne

Dr John Widdrington, Clinical Research Fellow, Institute of Cellular Medicine, Newcastle University, Newcastle upon Tyne

Dr Gavin Hudson, Research Fellow, Institute of Genetic Medicine, Newcastle University, International Centre for Life, Newcastle upon Tyne

Dr Angela Pyle, Senior Research Associate, Institute of Genetic Medicine, Newcastle University, International Centre for Life, Newcastle upon Tyne

Mr Jonathan Scott, Research Technician, Institute of Cellular Medicine, Newcastle University, Newcastle upon Tyne

Dr Marie-Helene Ruchaud Sparagano, Research Associate, Institute of Cellular Medicine, Newcastle University, Newcastle upon Tyne

Dr Kathryn Musgrave, Clinical Research Fellow, Institute of Cellular Medicine, Newcastle University, Newcastle upon Tyne

Julie Furneval, Band 6 Research Nurse, Integrated Critical Care Unit, City Hospitals Sunderland

Ashley Allan, Band 6 Research Nurse, Integrated Critical Care Unit, City Hospitals Sunderland

Dr Sarah Wiscombe, Clinical Research Fellow, Institute of Cellular Medicine, Newcastle University, Newcastle upon Tyne

Dr Patrick Kesteven, Consultant Haematologist, Newcastle upon Tyne Hospitals Foundation Trust

Prof Patrick Chinnery, Professor of Neurology and Head of the Department of Clinical Neurosciences, University of Cambridge, Cambridge

Prof Paul Corris, Professor of Thoracic Medicine, Institute of Cellular Medicine, Newcastle University, Newcastle upon Tyne

Introduction

Although sepsis is one of the oldest syndromes recognised within medicine, understanding of its biology remains incomplete. The healthcare burden from infection remains substantial. Severe sepsis is found in approximately 2% of hospital admissions in the USA, half of which require treatment in the intensive care unit (ICU)(1). As many as 30% of patients have sepsis on admission or during their ICU stay. The mortality associated with sepsis seems to be substantially higher than that of other syndromes leading to ICU admission (2) and it is the most common cause of death in non-surgical ICUs (3).

Risk factors for the development of severe sepsis are premorbid health status and physiological reserve of the host, causative organism, timeliness of intervention and host genetic characteristics. Gram-negative infections account for a greater proportion of severe sepsis than Gram-positive infections (4) and they are also associated with a worse outcome (5). Studies exploring genetic factors have concentrated on polymorphisms in nuclear genes involved in the innate inflammatory response (6), coagulation and fibrinolysis (1).

Impaired cellular respiration is important in the development of multi-organ dysfunction in severe sepsis (7). Natural variation in mitochondrial DNA (mtDNA) is an understudied area likely to yield insights into how the host genome may influence outcome in severe sepsis. Human mtDNA is maternally inherited and codes for 13 essential protein components of the mitochondrial respiratory chain. Individuals can be divided into mtDNA haplogroups on the basis of specific single nucleotide polymorphisms (SNPs) in their mitochondrial genome. The most common subdivision of mtDNA in Europe, haplogroup H, is associated with enhanced oxidative phosphorylation and is a strong independent predictor of survival after admission to the ICU with severe infection (8). The frequency of haplogroup H is approximately 40% in North East England (9).

Human survival from infection requires an appropriate inflammatory response: an unbalanced, hyperinflammatory response predisposes patients to overwhelming inflammation, while protracted immunosuppression is associated with organ dysfunction and heightened risk of nosocomial and opportunistic infections (10). An observational study performed in Newcastle suggested that patients with haplogroup H had a survival advantage, and higher fevers, when compared with non- H haplogroups (8). Recent data from our laboratory (manuscript in preparation) suggest that mitochondrial genes regulate the expression of TLR-4 and triggering receptor expressed on myeloid cells type 1 (TREM1) on human monocytes through interferon gamma-dependent pathways, which in turn influences the early inflammatory response to lipopolysaccharide (LPS).

Previous research on sepsis has focused on isolated mediators using a reductionist approach often derived from animal models (11). A genome wide study has questioned the validity of using murine models (12) whilst experimental human endotoxaemia shows much greater correlation with transcriptomic changes due to inflammatory stresses in human disease (13). Injection of LPS, a non-infectious Gram-negative bacterial cell-wall product, is a well-recognised, safe, investigational technique that has been used experimentally for over 50 years(14). The dose used is adjusted to body weight to ensure a standardised reaction in all participants. While we recognise that administration of low dose endotoxin is not a clinical model of sepsis, it does represent a significant improvement in experimental modeling over animal studies. Human endotoxaemia reproduces the earliest features of the pathogenesis of sepsis (which are almost impossible to study in the clinical setting), paving the way to define mechanisms of pathogenesis, and "drugable" targets.

The prevailing frequency of haplogroup H in the general population and the human endotoxaemia model together provide a unique opportunity to begin to explore the contribution of mitochondrial function to the early inflammatory response in sepsis. The aim of this seed application is therefore to test the hypothesis that ***mitochondrial haplogroup H leads to a more pronounced pro-inflammatory response to intravenous lipopolysaccharide in a model of human endotoxaemia.***

Experimental Plan using the Human Endotoxaemia Model

The human intravenous endotoxaemia model has been used for more than 50 years as a model of acute systemic inflammation, encountered in conditions such as sepsis and trauma. Low dose purified lipopolysaccharide (LPS, also referred to as endotoxin) from the cell membrane of *Escherichia coli* (*E.coli*) is administered to healthy volunteers resulting in transient, flu-like symptoms, and an acute systemic inflammatory response, which, at least partially, mimics the inflammatory response of early sepsis. The doses needed in humans to mimic the clinical entity of severe sepsis are ethically unacceptable.

The effects of Good Manufacturing Practice (GMP) grade LPS from *E.coli* are highly reproducible (15). Within an hour of the intravenous administration of LPS, volunteers experience varying degrees of flu-like symptoms e.g. chills, headache, myalgia , nausea, photophobia and sleepiness. In general the response is dose-dependent. Most subjects only experience symptoms for about two to six hours. The core temperature increases within one hour of administration and peaks at three to five hours and meets in the SIRS criterion for fever in most participants. Any drop in blood pressure is prevented by the administration of intravenous fluid, but an increase in heart rate of 20bpm within the first four hours is consistently seen. No severe cardiovascular complications resulting from endotoxin administration have been reported (15). Whilst, in some subjects the respiratory rate and minute ventilation increases with administration of high

doses of endotoxin (4ng/kg), this is less often seen using lower doses (14). The change in white blood cell count is a little more delayed, but in most participants the peak is seen by nine hours and fulfilling the SIRS leukocytosis criterion.

Our group has considerable previous (16) and on-going experience with LPS challenge studies. Our close liaison with van der Poll's group in Amsterdam and Gilroy's group in London give us confidence that we can safely extend our investigations to an intravenous model of human endotoxaemia using GMP grade endotoxin from the NIH endotoxin repository.

Following ethical approval, we shall recruit 30 healthy, non-smoking, volunteers aged 18 - 40. The sample size is pragmatic (given the novelty of this experimental design), informed by a previous observational, cohort study and by the distribution of haplogroups within the population (8). We shall dichotomise the study cohort into H and non-H haplogroups for analysis. We will attempt to recruit as many volunteers as possible up to a maximum of 30, but believe that analysis of data from 12 volunteers will be informative.

We will advertise the study at Newcastle University using mechanisms that have served us well in current and previous inhaled LPS studies. Participants providing informed, written consent will undergo an initial health screen and will be required to have no significant medical history or recent febrile illness, as well as normal physical examination, electrocardiography, oxygen saturation, full blood count, urea and electrolytes, and liver function tests. Blood will be drawn for determination of mitochondrial haplogroup, but we will be blinded to the results until study completion.

Volunteers will be asked to refrain from caffeine or alcohol for 24 hours before the study and will spend a full day (10 hours) in the Intensive Care Unit at City Hospitals Sunderland. Vital signs (pulse rate, blood pressure, temperature, respiratory rate and oxygen level) will be monitored at baseline and every 30 minutes throughout the day. A venous line will be inserted into each arm. Systemic inflammation will be induced by intravenous injection of a bolus of 2ng/kg of U.S reference *E. coli* endotoxin made available by the National Institute of Health (Bethesda, USA). Doses of up to 4ng/kg have been used but the inflammatory response appears to be similar whether 2ng/kg or 4ng/kg is used (15), Therefore in an attempt to reduce any unpleasant symptoms for volunteers the lower dose will be used. Participants can eat and drink at any point following injection of endotoxin. 20ml of blood will be drawn at baseline and at 90 minutes, 4, 6 and 10 hours. Volunteers will attend for blood sampling and follow-up the day after the study and then 7 days following the study.

Objectives

Primary Objective

The primary objective is to test the hypothesis that mitochondrial haplogroup predicts the inflammatory response in a model of human endotoxaemia, the primary outcome measure used will be the TNFα level 90 minutes after endotoxin administration.

Secondary Objectives

(i) To determine the expression of HLA-DR, TLR4 and TREM-1 by monocyte subsets in different haplogroups.
(ii) To determine the release of reactive oxygen species in blood in different haplogroups.
(iii) To determine the profile of additional proinflammatory and anti-inflammatory mediators in plasma in different haplogroups.
(iv) To determine mitochondrial DNA (mtDNA) copy number in plasma in different haplogroups.
(v) To determine the changes in leukocyte dynamics and function in response to intravenous LPS.
(vi) To measure the clinical response (temperature, pulse rate, blood pressure, respiratory rate and oxygen saturation) to endotoxin in different haplogroups.

Study Design

Participant Enrolment and Selection

An advert will be placed on Newcastle University email lists and notice boards. Potential participants will be asked to make contact with the research team only if they consider themselves to be healthy. Interested individuals will be invited to contact the research team who will send out information on the study (participant information sheets and screening consent form). Participants sent such information will be invited to contact the research team to arrange a screening visit (see below) or to decline participation. If no reply is received after 2 weeks, the research team will telephone the volunteer as a reminder.

Screening Visit

Healthy volunteers will be recruited from within Newcastle University. The screening visit will take the form of:

- a short history
- vital signs measurement (temperature, pulse rate, blood pressure)
- measurement of oxygen saturation breathing room air
- cardiorespiratory examination
- electrocardiogram
- blood sample for full blood count

- blood sample for urea & electrolytes assay, liver function tests and C-reactive protein
- urinary pregnancy test in women

Eligibility will be based on the results of this and inclusion and exclusion criteria (see inclusion/exclusion criteria).

Setting

All volunteers will be recruited through advertisement within Newcastle University. Volunteers will be screened at the preassessment clinic at City Hospitals Sunderland Foundation Trust. If found eligible for the study, they will be given time to consider if they wish to continue to study entry (minimum 24 hours) and invited to re-attend on a set day. Participants will be asked to attend the integrated critical care unit (ICCU, City Hospitals Sunderland Foundation Trust) for LPS administration. Downstream preparation and analysis of samples generated by the study will be performed in the Simpson lab, in the flow cytometry facilities (Centre for Life and Institute of Cellular Medicine), in the Wellcome Trust Centre for Mitochondrial Research (Centre for Life, eg for assessment of mitochondrial DNA), all Newcastle University. Processing of screening blood tests and routine clinical tests following administration of LPS will take place in the clinical laboratories at City Hospitals Sunderland.

Study Population

30 healthy participants will be recruited.

Inclusion criteria

- Healthy adult volunteers aged between 18 and 40 years of age
- Able to give informed consent

Exclusion criteria

A volunteer will not be eligible for inclusion in the study if any of the following criteria apply at entry:

1. Age <18 or >40 years.
2. Needle phobia.
3. Current participation in a clinical trial.
4. Known history of mitochondrial disease.
5. Past history of chronic respiratory disease.
6. Past or current history of conditions known to affect immunity or cardiac function (e.g. diabetes, ischaemic heart disease, congenital heart disease, valvular heart disease, cirrhosis, chronic renal impairment, recurrent urinary tract infection).

7. Known history of immunodeficiency.
8. Known history of hepatitis B/C or HIV.
9. History of an acute intercurrent cardiorespiratory illness.
10. Pregnant or breastfeeding.
11. Any current medication (except oral contraceptive pill).
12. Current history of smoking.
13. Reported alcohol intake >21 units per week.
14. Abnormal physical signs detected at cardiorespiratory examination.
15. Temperature >37.3 degrees celsius.
16. Oxygen saturation <95% breathing room air.
17. Haemoglobin outside the laboratory reference range.
18. Platelet count less than 100 x 10^9/l or greater than 650 x 10^9/l.
19. Total white cell count outside the laboratory reference range.
20. Any deviation of greater than 20% from normal in the differential white cell count.
21. Serum sodium, potassium, creatinine outside the laboratory reference range.
22. Blood urea greater than 10mg/dl.
23. Bilirubin greater than 30micromol/l.
24. Alanine transferase greater than twice the upper limit of the laboratory reference range.
25. Allergy to the any of the constituents of Hartmann's solution.
26. Currently participating in a clinical trial that the chief investigator feels would interfere with the analysis carried out as a result of this study.

Consent

All eligible volunteers will be given written and verbal information regarding study participation. All will be asked to give written consent with a minimum 24 hours to consider entering the study. Consent will be taken by Dr Rostron or Dr Roy. Pseudoanonymised data (linked by a unique study code) will be entered on an excel database.

Ineligible and non-recruited participants

For volunteers found to be ineligible at screening, or eligible but not subsequently entered into the study, the reason for ineligibility or non-recruitment will be entered on the excel database. Only anonymised data will be entered on to the database and this will include gender, age, “ineligible” or “non-recruitment” and the associated reason. If found ineligible, permission will be sought from participants to contact their GP with the results of screening tests and any further action required.

Study visit 1 (day 0)

Volunteers satisfying study criteria, wishing to proceed with the study, and providing written informed consent will attend the integrated critical care unit

(ICCU), City Hospitals Sunderland on an agreed day. Volunteers will be asked to refrain from caffeine or alcohol for 24 hours before the study and during study visit 1. Volunteers will spend a full day (10 hours) in the Intensive Care Unit at City Hospitals Sunderland. A brief history (regarding symptoms of any acute illness) will be sought. In female participants, a repeat urinary pregnancy test will be performed and will be reviewed by a medical practitioner who will offer appropriate advice. Only those participants who have a negative test will be allowed to proceed with the study. Vital signs (pulse rate, blood pressure, temperature, respiratory rate and oxygen level) will be monitored at baseline and every 30 minutes throughout the day. A venous cannula will be inserted into each arm. Intravenous fluid (Hartmann's solution, a solution with concentration of salts similar to that of blood) will be administered via one of the intravenous cannulae. Systemic inflammation will be induced by intravenous injection of a bolus of 2ng/kg of U.S reference E. coli endotoxin into the other intravenous cannula. Participants can eat and drink at any point following injection of endotoxin, but will be asked to refrain from caffeine and alcohol during the day of study visit 1. 80ml will be drawn at baseline, 90 minutes, 6 and 10 hours. 20ml of blood will be drawn at 4 hours following injection of endotoxin. Volunteers are advised to use a suitable method of contraception for 48 hours following injection of LPS.

Study visit 2 (day 1)

Volunteers will be asked to attend the ICCU at City Hospitals Sunderland. All will undergo a brief history, examination and blood sampling (20mls).

Study visit 3 (day 7)

Volunteers will be asked to attend the ICCU at City Hospitals Sunderland. All will undergo a brief history, examination and blood sampling (20mls).

Study Assessments: processing and analysis of blood samples

Screening visit blood samples will be sent to the hospital laboratories at City Hospitals Sunderland for assessment of full blood count, urea and electrolytes, liver function tests and C-reactive protein (CRP) (8mls). A sample will also be taken for mitochondrial haplogroup typing (2mls) which will be performed at the Centre for Life, Newcastle University

Blood samples will be taken at baseline, 90 minutes, 4, 6 and 10 hours after administration of intravenous LPS. Blood sample volume that will be drawn will be approximately 80mls will be drawn at baseline, 90 minutes, 6 and 10 hours. 20mls at 4 hours. Full blood count analysis and measurement of serum CRP and procalcitonin (PCT) will be done in the hospital laboratories at City Hospitals Sunderland NHS Foundation Trust. All other sampling will be performed at Newcastle University. Assessment of free mitochondrial DNA (mtDNA) will performed at the Centre for Life, all other scientific processing and analysis will be undertaken in the Medical School, Newcastle University.

Sampling at baseline, 90 minutes, 6 and 10 hours:

- Full blood count (2mls)
- Serum (9 mls) for CRP, PCT, cytokines and free mtDNA
- Flow cytometry (4 mls)
- Prothrombotic markers (5mls)
- Transcriptome (RNA sequencing), epigenome (ATAC sequencing) and proteome analysis (mass cytometry) of peripheral blood leukocytes (60mls) isolated by flow cytometry.

Sampling at 4 hour, 24 hour and 7 day time points:

- Full blood count (2mls)
- Serum (9 mls) for CRP, PCT, cytokines and free mtDNA
- Flow cytometry (4 mls)
- Prothrombotic markers (5mls)

The total volume of blood sampled over the study period (390ml) will be similar to the volume of blood drawn for the donation of blood. Scientific samples will be stored in a locked freezer, in swipe-card protected premises in the Institute of Cellular Medicine. Freezers are accessible to the research team, and to individuals who maintain university research freezers and their governance. Samples will be destroyed after five years, unless we believe there is advantage to keeping them, in which case we will take advice from sponsor as how to proceed.

Data Analysis

Data from analysis of blood samples will be compared between haplogroup H and non-H haplogroups for all time-points along with changes from baseline.

Risk Assessments

Blood sampling

Blood sampling can be accompanied by discomfort or by vasovagal symptoms. Risks are minimised through all samples being taken in a fully supported medical facility. Blood will be drawn whilst the volunteer is positioned on a bed or in a self-reclining chair. Volunteers feeling syncopal will be positioned supine and venepuncture will be discontinued. The total volume of blood to be sampled over the entire study is less than the volume required for a standard blood donation.

Intravenous administration of LPS

LPS will be administered to volunteers whilst they are monitored in the integrated critical care unit (ICCU) at City Hospitals Sunderland. Participants will be observed for 10 hours following LPS administration.

Expected symptoms include chills, headache, photophobia (aversion to bright lights), myalgia (muscle aches), arthralgia (joint pains), nausea (feeling sick), and rarely, vomiting. Peak symptom intensity occurs around 1 - 2 hr post-injection, abating afterwards to baseline by 6 - 8 hr. No severe or sustained adverse effects secondary to endotoxin at this dose have been reported. Less than 5% of volunteers feel the need to treat these symptoms with medicines such as paracetamol/acetaminophen or non-steroidal agents by mouth or IV (e.g., aspirin, ibuprofen). Such agents may alter the inflammatory response and their use should be recorded. Other adverse signs include fever, increase or decrease in heart rate and decrease in blood pressure (hypotension), medications required to treat adverse effects will be readily available (e.g. intravenous fluid in the event of a significant episode of hypotension or paracetamol in the event of significant symptoms of fever). Significant hypotension only occurs rarely (less than 1 in 1000 volunteers) and is rapidly treated by the administration of an intravenous fluid bolus.

Vital signs will be measured at baseline, then every 30 minutes until six hours following LPS administration and then hourly thereafter until participants are allowed home. Symptoms scores for nausea, muscle aches, headache and chills will be recorded.

3. Burden of time

Volunteers wil be required to attend for a screening visit, a ten hour study day for induction of inflammation, observation and sampling, followed by two further study visits for follow-up and sampling. Volunteers will required to attend the Integrated Critical Care Unit of City Hospitals Sunderland for study visits. The host institution and follow-up study visits will facilitate safety monitoring for volunteers. In recognition of the burden on the volunteer in terms of time, volunteers will be offered £250 on study completion.

Adverse Events

Our research group has experience with the LPS challenge models. We have successfully administered 60µg of inhaled LPS to young, healthy participants with no significant adverse events recorded (16).

Intravenous LPS has been administered to healthy volunteers for over 40 years. There is a substantial body of evidence to support the safe administration of higher doses (4ng/kg) of intravenous LPS than we intend to use in this study. Nevertheless, we feel that we must remain vigilant in detecting and recording any adverse events as a result of exposure to LPS or other procedures undertaken. Whilst recognising that this current work is not a clinical trial, our groups' previous work (which included a clinical trial) benefited from classifying

and monitoring adverse events in the manner described below, and we have elected to continue using this terminology in this research.

Definitions
An ***adverse event*** (AE) is any untoward medical occurrence in a study participant.
A ***serious adverse event*** (SAE) is any untoward medical occurrence in a study participant or effect that:

- results in death
- is life threatening (i.e. the subject was at risk of death at the time of the event; it does not refer to an event which hypothetically might have caused death if it were more severe)
- requires admission to hospital as an in-patient beyond one calendar day or requires the volunteer to stay longer than 12 hours following injection of lipopolysaccharide
- results in persistent or significant disability or incapacity
- is a congenital anomaly or birth defect

If an adverse event is detected, a member of the research team will make an assessment of seriousness as defined by the above definitions. If the event is deemed to be serious (SAEs) a member of the research team will then consider if the event was:

- **Related** – that is it resulted from administration of research procedures and/or
- **Unexpected –** that is a type of event that is not identified as an expected occurrence

Detecting and reporting AE and SAEs

All AEs and SAEs will be recorded from the time a participant consents to join the study until 24 hours after completing the final study assessments. A medically qualified member of the research team will ask about the occurrence of AEs/SAEs during the study. Information to be collected includes type of event, onset date, researcher assessment of implications, if any, for safety of participants and how these will be addressed, date of resolution as well as treatment required, investigations needed and outcome. All information will be recorded in the participants study file.
An AE/SAE may necessitate discontinuation of a given part of the study (but progression through the remainder of the study) or complete and immediate discontinuation of any further participation. All participants will maintain the right to discontinue or completely withdraw from the study at any time for any reason, or without stating a reason. The reason and circumstances for premature discontinuation (where known) will be documented in the participant's study file.

If a SAE has occurred, the research team must report the information to Newcastle upon Tyne Hospitals R&D within 24 hours. The SAE form must be completed as thoroughly as possible with all available details of the event, signed by the Investigator or designee. The SAE form should be transmitted by fax or by hand to the office.

NUTH R&D is responsible for reporting SAEs that are considered to be related and unexpected as described above to the Research Ethics Committee (REC) that approved the study (main REC) within 15 days of becoming aware of the event using the NRES Reporting of SAE Form. The Co-ordinator of the main REC should acknowledge receipt of related, unexpected safety report within 30 days.

Discharge criteria

The attending clinician will ensure that the participant's symptoms have settled and that their observations are trending to normal (all altered parameters, e.g., elevated heart rate and temperature, demonstrating consistent reduction toward baseline values) prior to sanctioning the end of observation and subsequent discharge. After bolus injection of 2ng/kg LPS symptoms normally fully abate by 6 - 8 hr. Individual observations follow overlapping but discrete time-courses. These have normally returned to baseline by 10hr. All monitoring and venous cannulae will be removed and haemostasis will be ensured. It will be confirmed that the participant is happy to be discharged home and has the contact details of the research team in case of any concern.

End of study

The study will be completed on day 7 of the final volunteer. Scientific analysis of samples in accordance with the experimental plan may be performed after this.

Process if new information is available

If any further information becomes available that leads to further studies then consent will be obtained from volunteers and ethic approval will be sought.

Criteria for terminating the project

In the event of a serious adverse event, the project will be terminated.

References

1. Angus DC, van der Poll T. Severe sepsis and septic shock. Vol. 369, The New England journal of medicine. United States; 2013. p. 2063.
2. Vincent JL, Marshall JC, Ñamendys-Silva S a., François B, Martin-Loeches I, Lipman J, et al. Assessment of the worldwide burden of critical illness: The Intensive Care Over Nations (ICON) audit. Lancet Respir Med. 2014;2(5):380–6.
3. Anel R, Kumar A. Human endotoxemia and human sepsis: limits to the model. Crit Care. England; 2005 Apr;9(2):151–2.
4. Vincent J, Marshall J, Anzueto A, Martin CD, Gomersall C. International

Study of the Prevalence and Outcomes of Infection in Intensive Care Units. J Am Med Assoc. 2009;302(21):2323–9.
5. Cohen J, Cristofaro P, Carlet J, Opal S. New method of classifying infections in critically ill patients. Crit Care Med. United States; 2004 Jul;32(7):1510–26.
6. Rautanen A, Mills TC, Gordon AC, Hutton P, Steffens M, Nuamah R, et al. Genome-wide association study of survival from sepsis due to pneumonia: an observational cohort study. Lancet Respir Med. England; 2015 Jan;3(1):53–60.
7. Brealey D, Singer M. Mitochondrial Dysfunction in Sepsis. Curr Infect Dis Rep. 2003 Oct;5(5):365–71.
8. Baudouin S V, Saunders D, Tiangyou W, Elson JL, Poynter J, Pyle A, et al. Mitochondrial DNA and survival after sepsis: a prospective study. Lancet (London, England). England; 2005 Dec;366(9503):2118–21.
9. Chinnery PF, Taylor GA, Howell N, Andrews RM, Morris CM, Taylor RW, et al. Mitochondrial DNA haplogroups and susceptibility to AD and dementia with Lewy bodies. Neurology. UNITED STATES; 2000 Jul;55(2):302–4.
10. Hotchkiss RS, Monneret G, Payen D. Sepsis-induced immunosuppression: from cellular dysfunctions to immunotherapy. Nat Rev Immunol. England; 2013 Dec;13(12):862–74.
11. Gingles NA, Alexander JE, Kadioglu A, Andrew PW, Kerr A, Mitchell TJ, et al. Role of genetic resistance in invasive pneumococcal infection: identification and study of susceptibility and resistance in inbred mouse strains. Infect Immun. UNITED STATES; 2001 Jan;69(1):426–34.
12. Xiao W, Mindrinos MN, Seok J, Cuschieri J, Cuenca AG, Gao H, et al. A genomic storm in critically injured humans. J Exp Med. United States; 2011 Dec;208(13):2581–90.
13. Seok J, Warren HS, Cuenca AG, Mindrinos MN, Baker H V, Xu W, et al. Genomic responses in mouse models poorly mimic human inflammatory diseases. Proc Natl Acad Sci U S A. United States; 2013 Feb;110(9):3507–12.
14. Calvano SE, Coyle SM. Experimental human endotoxemia: a model of the systemic inflammatory response syndrome? Surg Infect (Larchmt). United States; 2012 Oct;13(5):293–9.
15. Andreasen AS, Krabbe KS, Taudorf S, Pedersen BK, Møller K. Human Endotoxemia as a Model of Systemic Inflammation. 2008;1697–705.
16. Barr LC, Brittan M, Morris AC, McAuley DF, McCormack C, Fletcher AM, et al. A randomized controlled trial of peripheral blood mononuclear cell depletion in experimental human lung inflammation. Am J Respir Crit Care Med. United States; 2013 Aug;188(4):449–55.

Poisson linear mixed model for cell type composition analysis

1.1 Log linear model for two-way tables

Let Y_{ij} be the cell type count observed from the sample i ($i = 1, \ldots, N$) for the cell type j ($j = 1, \ldots, J$). A simple test of independence between samples and cell types (to make sure there is no differential cell type abundance among samples) would be to fit a log-linear model for two-way tables[66]:

$$Y_{ij} \overset{i.i.d.}{\sim} \mathrm{Pois}(\lambda_{ij}),$$
$$\log \lambda_{ij} = \mu + a_i + b_j + \varepsilon_{ij},$$

for $i = 1, \ldots, N$ and $j = 1, \ldots, J$. Here we assume Y_{ij} follows a Poisson distribution with a mean λ_{ij}, the logarithm of which can be decomposed into the grand mean μ, the sample mean a_i, the cell type mean b_j and the interaction term ε_{ij} (between sample i and cell type j). In order to assess the two-way table is independent, we assume $\{a_i, b_j, \varepsilon_{ij}\}$ follow the independent normal distributions with variance parameters $\{\nu^2, \omega^2, \sigma^2\}$, such that

$$a_i \overset{i.i.d.}{\sim} \mathcal{N}(0, \nu^2), \quad b_j \overset{i.i.d.}{\sim} \mathcal{N}(0, \omega^2), \quad \varepsilon_{ij} \overset{i.i.d.}{\sim} \mathcal{N}(0, \sigma^2),$$

for $i = 1, \ldots, N$ and $j = 1, \ldots, J$, where the variance σ^2 is the parameter of interest. If there is no interaction (*i.e.*, no differential cell type abundance among samples), the variance estimate should become $\hat{\sigma}^2 \longrightarrow 0$.

1.2 Variance explained by sample metadata

Suppose $\sigma^2 > 0$, this model enables us to explore the relative importance of a wide range of clinical/technical factors in determining cell type composition. Let x_{ik} be a value of the factor k ($k = 1, \ldots, K$) for the sample i, which is either a numerical value (*e.g.* patient's age) or a categorical value of L_k levels (*e.g.*, disease severity with $L_k = 6$: healthy, asymptomatic, mild, moderate, severe and critical). Then the mean of the poisson distribution can be extended with extra interaction terms between cell type and each of the K factors, such that,

$$\log \lambda_{ij} = \mu + a_i + b_j + \sum_{k=1}^{K} \eta_{ijk} + \varepsilon_{ij}$$
$$\eta_{ijk} = \begin{cases} \boldsymbol{z}_{ik}^T \boldsymbol{u}_{jk} & \text{factor } k \text{ is a categorical variable with } L_k \text{ levels,} \\ \tilde{x}_{ik} u_{jk} & \text{factor } k \text{ is a numerical variable } (L_k = 1), \end{cases}$$

where η_{ijk} denotes the interaction effect between the cell type j and the factor k for the sample i, which is modelled by the interaction effect $\boldsymbol{u}_{jk} = (u_{jk1}, \ldots, u_{jkL_k})^T$. Here $\tilde{x}_{ik}$ denotes the scaled value of x_{ik} (*i.e.*, sample mean and variance of the numerical factor k is 0 and 1) and $\boldsymbol{z}_{ik}^T = (z_{ik1}, \ldots, z_{ikL_k})$ is a design vector whose element is

$$z_{ikl} = \begin{cases} 1 & x_{ik} = l, \\ 0 & \text{otherwise,} \end{cases}$$

for $l = 1, \ldots, L_k$. The interpretation of u_{jkl} is the log fold change of the jth cell type abundance for the lth level of categorical factor k against the grand mean. For a numerical factor k, u_{jk} is a scalar value reflecting the log fold change of the jth cell type abundance in response to one unit change of scaled data $\tilde{x}_{ik}$.

Because the factors are colinear and often confounding each other (unless the study is the designed experiment), we further assume those interaction effects follow multivariate normal distributions:

$$\boldsymbol{u}_{jk} \overset{i.i.d.}{\sim} \mathcal{N}(\boldsymbol{\mu}_k, \delta_k^2 I_{L_k}),$$

where $\boldsymbol{\mu}_k$ denotes the mean vector around which the variance parameter δ_k^2 is estimated, which reflects the relative contribution of each factor on cell type composition variation. Here the mean vector $\boldsymbol{\mu}_k$ is not the parameter of interest, therefore for the categorical factors, we regressed out from the model by assuming another multivariate normal distribution:

$$\boldsymbol{\mu}_k \overset{i.i.d.}{\sim} \mathcal{N}(0, \gamma_k^2 I_{L_k})$$

so that the number of parameters can be significantly reduced from L_k to 1.

1.3 Likelihood ratio test

To properly assess the statistical significance of each factor that explains a significant amount of interaction variation, we compared the the following two models:

$$H_0 : \delta_k^2 = 0$$
$$H_1 : \delta_k^2 > 0$$

Then the likelihood ratio test statistics follows the χ^2 distribution with one degree of freedom under the null hypothesis (H_0). In order to adjust multiple testing, we used the number of factors (*i.e.*, K, which is the same as the number of variance parameters γ_k^2 for cell type interaction) for the total number of tests.

1.4 Posterior mean and variance of random effects

In general, the generalised linear mixed model has no closed form of the marginal likelihood, because the integral with respect to random effects is intractable. Therefore an approximation becomes one of the natural alternatives. A well-known method of approximate integrals is named after Laplace (used in `lme4` package on `R`). Let $Y^T = (Y_{11}, \ldots, Y_{NJ})$ be the vector of cell type counts and

$$\boldsymbol{u}^T = (a_1, \ldots, a_N, b_1, \ldots, b_J, \boldsymbol{\mu}_1^T, \ldots, \boldsymbol{\mu}_K^T, \boldsymbol{u}_{11}^T, \ldots, \boldsymbol{u}_{JK}^T, \varepsilon_{11}, \ldots, \varepsilon_{NJ})$$

be the vector of all random effects, the marginal likelihood can be approximated as

$$p(Y) = \int p(Y|\boldsymbol{u})p(\boldsymbol{u})d\boldsymbol{u} \approx c|H|^{-\frac{1}{2}}\exp\{L(\tilde{\boldsymbol{u}})\},$$

where $L(\boldsymbol{u}) = \log p(Y|\boldsymbol{u})p(\boldsymbol{u})$ denotes the complete log likelihood function whose maximum is attained at $\boldsymbol{u} = \tilde{\boldsymbol{u}}$ with the first derivative $L'(\tilde{\boldsymbol{u}}) = \mathbf{0}$ and the hessian matrix $H = -L''(\tilde{\boldsymbol{u}})$, and c denotes a constant multiplication. This gives an approximated posterior distribution of $\boldsymbol{u}$ given Y, such that

$$\boldsymbol{u}|Y \sim \mathcal{N}(\tilde{\boldsymbol{u}}, H^{-1}).$$

1.5 Standard error of model parameters

The log marginal likelihood $L(\boldsymbol{\theta}|Y) = \log p(Y)$ after integrating out the random effects $\boldsymbol{u}$ is a function of model parameters $\boldsymbol{\theta} = (\mu, \nu, \omega, \sigma, \gamma_1, \ldots, \gamma_K, \delta_1, \ldots, \delta_K)$. The standard error of $\boldsymbol{\theta}$ can be computed from the inverse matrix of the Fisher score matrix

$$I = -\left.\frac{\partial^2 L(\boldsymbol{\theta}|Y)}{\partial\boldsymbol{\theta}\partial\boldsymbol{\theta}^T}\right|_{\boldsymbol{\theta}=\hat{\boldsymbol{\theta}}}$$

where the likelihood function attains its maximum value at $\boldsymbol{\theta} = \hat{\boldsymbol{\theta}}$ with $L'(\hat{\boldsymbol{\theta}}|Y) = \mathbf{0}$.

1.6 Overdispersion due to technical variation

Although the Poisson model does not explicitly take account of the overdispersion in the cell type count data (unlike Negative Binomial distributions), the interaction term ε_{ij} between sample and cell type partly captures the discrepancy between $\mathrm{E}[Y_{ij}]$ and $\mathrm{Var}(Y_{ij})$, since

$$\mathrm{Var}(Y_{ij}|\boldsymbol{u}_{ij}) = \mathrm{E}[Y_{ij}|\boldsymbol{u}_{ij}] + \mathrm{E}[Y_{ij}|\boldsymbol{u}_{ij}]^2(e^{\sigma^2} - 1),$$

where $\boldsymbol{u}_{ij} = (a_i, b_j, \eta_{ij1}, \ldots, \eta_{ijK})^T$. This fact suggests the model becomes overdispersed when $e^{\sigma^2} > 1$ given $\boldsymbol{u}_{ij}$.

A

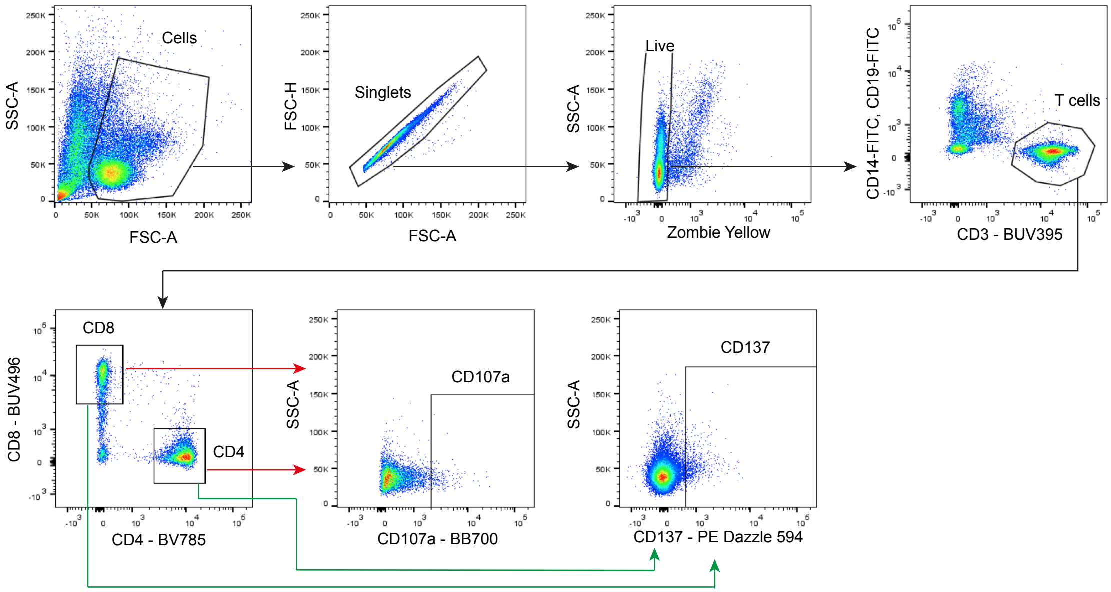

Cells
SSC-A
FSC-A
Singlets
FSC-H
FSC-A
Live
SSC-A
Zombie Yellow
T cells
CD14-FITC, CD19-FITC
CD3 - BUV395
CD8
CD4
CD8 - BUV496
CD4 - BV785
CD107a
SSC-A
CD107a - BB700
CD137
SSC-A
CD137 - PE Dazzle 594
