# Supplementary material for: Single-cell multi-omics analysis of the immune response in COVID-19
Source: Nat Med. 2021 Apr 20;27(5):904–16. doi: 10.1038/s41591-021-01329-2 (PMC8121667; doi:10.1038/s41591-021-01329-2)
Supplement: Supplementary file 2 — Reporting Summary [file 41591_2021_1329_MOESM2_ESM.pdf]

nature research

Corresponding author(s): Sarah A Teichmann, Menna R Clatworthy, John C Marioni, Berthold Gottgens, Muzlifah Haniffa

Last updated by author(s): 2021/03/17

Reporting Summary

Nature Research wishes to improve the reproducibility of the work that we publish. This form provides structure for consistency and transparency in reporting. For further information on Nature Research policies, see our Editorial Policies and the Editorial Policy Checklist.

Statistics

For all statistical analyses, confirm that the following items are present in the figure legend, table legend, main text, or Methods section.

n/a	Confirmed	
☐	☒	The exact sample size (*n*) for each experimental group/condition, given as a discrete number and unit of measurement
☐	☒	A statement on whether measurements were taken from distinct samples or whether the same sample was measured repeatedly
☐	☒	The statistical test(s) used AND whether they are one- or two-sided<br>*Only common tests should be described solely by name; describe more complex techniques in the Methods section.*
☐	☒	A description of all covariates tested
☐	☒	A description of any assumptions or corrections, such as tests of normality and adjustment for multiple comparisons
☐	☒	A full description of the statistical parameters including central tendency (e.g. means) or other basic estimates (e.g. regression coefficient) AND variation (e.g. standard deviation) or associated estimates of uncertainty (e.g. confidence intervals)
☐	☒	For null hypothesis testing, the test statistic (e.g. *F*, *t*, *r*) with confidence intervals, effect sizes, degrees of freedom and *P* value noted<br>*Give P values as exact values whenever suitable.*
☒	☐	For Bayesian analysis, information on the choice of priors and Markov chain Monte Carlo settings
☒	☐	For hierarchical and complex designs, identification of the appropriate level for tests and full reporting of outcomes
☐	☒	Estimates of effect sizes (e.g. Cohen's *d*, Pearson's *r*), indicating how they were calculated

Our web collection on statistics for biologists contains articles on many of the points above.

Software and code

Policy information about availability of computer code

Data collection

No software was used for data collection.

Data analysis

All data analysis scripts are available on https://github.com/scCOVID-19/COVIDPBMC

Alignment and quantification
Droplet libraries were processed using Cellranger v4.0. Reads were aligned to the GRCh38 human genome concatenated to the SARS-Cov-2 genome (NCBI SARS-CoV-2 isolate Wuhan-Hu-1) using STAR50 (v2.5.1b) and unique molecular identifiers (UMIs) deduplicated. CITE-seq UMIs were counted for GEX and ADT libraries simultaneously to generate feature X droplet UMI count matrices.
Doublet identification
Newcastle:
Scrublet (v0.2.1) was applied to each sample to generate a doublet score. These formed a bimodal distribution so the tool’s automatic threshold was applied.

Cambridge:
Non-empty droplets were called within each multiplexed pool of donors using the emptyDrops function implemented in the Bioconductor package DropletUtils (v1.10.3), using a UMI threshold of 100 and FDR of 1%. The probability of being a doublet was estimated for each cell per sample (that is one 10x lane) using the “doubletCells” function in scran based on highly variable genes (HVGs). Next, we used “cluster_walktrap” on the SNN-Graph that was computed on HVGs to form highly resolved clusters per sample. Per-sample clusters with either a median doublet score greater than the median + 2.5 x MAD or clusters containing more than the median + 2.5 MAD genotype doublets were tagged as doublets. This was followed by a second round of highly-resolved clustering across the whole data set, in which again cells belonging to clusters with a high proportion (> 60%) of cells previously labelled as doublets were also defined as doublets.

London:

For pooled donor CITE-seq samples, the donor ID of each cell was determined by genotype-based demultiplexing using souporcell version 251. Souporcell analyses were performed with 'skip_remap' enabled and a set of known donor genotypes given under the 'common_variants' parameter. The donor ID of each souporcell genotype cluster was annotated by comparing each souporcell genotype to the set of known genotypes. Droplets that contained more than one genotype according to souporcell were flagged as 'ground-truth' doublets for heterotypic doublet identification. Ground-truth doublets were used by DoubletFinder 2.0.352 to empirically determine an optimal 'pK' value for doublet detection. DoubletFinder analysis was performed on each sample separately using 10 principal components, a 'pN' value of 0.25, and the 'nExp' parameter estimated from the fraction of ground-truth doublets and the number of pooled donors.
CITE-seq background signal removal
Background antibody- and non-specific staining was subtracted from ADT counts in each data set from the 3 data acquisition sites separately. ADT counts for each protein were first normalised using counts per million (CPM) and log transformed, with a +1 pseudocount. To estimate the background signal for each protein, a 2-component gaussian mixture model (GMM), implemented in the mclust (v5.4.7) R package function Mclust, was fit across the droplets with a total UMI count > 10 and < 100 from each experimental sample separately. The mean of the first GMM component for each protein was then subtracted from the log CPM from the QC-passed droplets in the respective experimental sample.

Quality control, normalisation, embedding and clustering
Combined raw data from the three centres was filtered to remove those that expressed fewer than 200 genes and >10% mitochondrial reads. Data was normalised (scanpy: normalize_total), log+1 corrected (scanpy: log1p) and highly variable genes identified using the Seurat vst algorithm (scanpy: highly_variable_genes). Harmony was used to adjust principal components by sample ID and used to generate the neighbourhood graph and embedded using UMAP. Clustering was performed using the Leiden algorithm with an initial resolution of 3. For initial clustering, differentially expressed genes were calculated using Wilcoxon rank-sum test.
Relative importance of metadata on cell type composition
The number of cells for each sample (N=110 samples in total with complete metadata) and cell type (18 different cell types in total) combination was modelled with a generalised linear mixed model with a Poisson outcome (see Supplementary File 2 for detail). The 5 clinical factors (COVID-19 swab result, age, sex, disease severity at day 0 and days from onset) and the 2 technical factors (patient and sequencing centre) were fitted as random effects to overcome the collinearlity among the factors. The effect of each clinical/technical factor on cell type composition was estimated by the interaction term with the cell type . The likelihood ratio test was performed to assess the statistical significance of each factor on cell type abundance by removing one interaction term from the full model at a time. The number of factors was used to adjust multiple testing with the Bonferroni approach. The 'glmer' function in the lme4 package implemented on R was used to fit the model. The standard error of variance parameter for each factor was estimated using the numDeriv package.

Cydar Analysis
We utilized cydar to identify changes in cell composition across the different severity groups based on the protein data alone. First, the background-corrected protein counts from the three different sites were integrated using the 'fastMNN' method (k = 20, d = 50, cos.norm = TRUE) in batchelor (v1.6.2). The batch-corrected counts for 188 proteins (4 rat/mouse antibody isotypes were removed) were then used to construct hyperspheres using the 'countCells' function (downsample = 7) with the tolerance parameter chosen so that each hypersphere has at least 20 cells which was estimated using the 'neighborDistances' function. To assess whether the abundance of cells in each hypersphere are associated with disease status, hypersphere counts were analyzed using the quasi-likelihood (QL) method in edgeR (v3.32.1). After filtering out hyperspheres with an average count per sample below 5 we fitted a mean-dependent trend to the NB dispersion estimates. The trended dispersion for each hypersphere was used to fit a NB GLM using the log-transformed total number of cells as the offset for each sample and blocking for sex, age and batch. The QL F-test was used to compute P values for each hypersphere which were corrected for multiple testing using the spatial FDR method in cydar.

Comparisons of PBMC annotation using the Azimuth tool
The final annotation of PBMCs was compared to a published PBMC annotation using the Azimuth tool (http://azimuth.satijalab.org/app/azimuth). Because of size restrictions of 100,000 cells, our data was subsampled to 10% of the total cells. After running the algorithm, results with a prediction score < 0.5 were removed (5.8% of total removed). For each cluster in the COVID-19 PBMC data, the percentage of cells mapped to each cluster in the Azimuth annotation was calculated.

Interferon, TNF and JAK-STAT response scoring
A list of genes related to response to type I interferons was obtained from the GSEA Molecular Signatures Database (MSigDB) (GO: 0034340). Enrichment of the interferon score was measured using the tl.score_genes tool in scanpy which subtracts the average expression of all genes in the dataset from the average expression of the genes in this list. The scores were averaged across clusters and clinical status and expressed as a fold-change over the interferon score in the equivalent healthy cluster.

kBET analysis
The kBET53 algorithm (https://github.com/theislab/kBET) was run for each cluster defined in Fig. 1 using the Uniform manifold and projection (UMAP) coordinates generated from Harmony-adjusted principal components, and the sample number as the batch factor. The same procedure was then performed using the same annotation but using the UMAP coordinates generated from non-Harmony-adjusted principal components. The resultant rejection rates were averaged across clusters and compared using a Wilcoxon paired signed rank test.

Bronchoalveolar lavage data analysis
ScRNAseq data from BAL was obtained from GEO (accession number GSE14592622). Raw data was analysed using the same pipeline as PBMC data, specifically using the same quality control cut-offs (min of 200 genes and <10% mitochondrial reads/cell) and batch-corrected using Harmony by donor ID. To gain greater resolution of mononuclear phagocytes the DC and macrophages were analysed with further rounds of sub-clustering to identify DC1, DC2 and mature DC.

PAGA analysis of blood monocytes and BAL macrophages
Annotated raw expression datasets of BAL macrophages and COVID-19 PBMCs were merged and data log-normalised and scaled as for the original datasets. The top 3000 highly variable genes were chosen using the Seurat "vst" method and used for downstream analysis. Principal components were batch corrected by donor and used to build a neighborhood graph. The PAGA tool in scanpy (tl.paga) was used to generate the abstracted graph between clusters.

CellphoneDB
CellphoneDB54 was used to assess putative interactions between monocytes (CD14_mono, CD83_CD14_mono, C1_CD16_mono, CD16_mono, Prolif_mono) and platelets. The tool was run for 100 iterations and an expression threshold of 0.25 (limiting the analysis to

genes expressed by 25% of cells). For downstream analysis we focused on interactions between platelets and any monocyte subset.

Platelet activation
Differentially expressed genes in platelets between healthy controls and COVID-19 were filtered for those predicted to be involved in platelet activation (https://www.gsea-msigdb.org/gsea/msigdb/cards/ REACTOME_PLATELET_ACTIVATION_SIGNALING_AND_AGGREGATION)

HPSC commitment scoring
HPSCs were subsetted from the data and Leiden clusters generated using the same pipeline and parameters as for the whole PBMC dataset. Differentially expressed genes between the HSPC clusters that showed evidence of lineage commitment (MK, Erythroid and Myeloid) were calculated using FindAllMarkers tool in Seurat (with thresholds of genes expressed by 25% of cells and with a log fold-change of 0.25) and genes with an adjusted p-value cut-off of 0.05 were used to generate gene signatures for each. Enrichment of these signatures in the CD38 negative and CD38 positive HSPC clusters were calculated using the tl.score_genes in scanpy (v1.6.0). The average expression of these enrichment scores in the CD38 negative and CD38 positive HSPC clusters was averaged by donor then compared across clinical states. Differences between groups were assessed using ANOVA with pairwise comparisons using Tukey's test.

Multiplex cytokine analysis
The values of each analyte were detected using the MAGPIX® system and analysed using the ProcartaPlex Analyst version 1.0 Software (ThermoFisher Scientific).

Flow Cytometry of stimulated cells
Data were analysed by FlowJo V10 (BD Biosciences).

GSEA analysis
Pre-ranked gene set analysis (prGSEA) on MSigDB v7.2 Hallmark genesets55 was performed using pre-ranked gene lists with fgsea56 in R. Genes were pre-ranked according to signed -log10 P-values for all prGSEA procedures. For B cells, generation of rank gene list was performed using Wilcoxon rank sum test (via tl.rank_genes_groups in scanpy) with each Day 0 COVID statuses (asymptomatic to symptomatic critical) as the "tests" versus Day 0 Healthy samples as "reference/control".

T cell clustering, annotation and visualisation
Droplets labelled as T cells ("CD4", "CD8", "Treg", "MAIT", "gdT") were subset from those in Fig. 1B and re-clustered using a set of HVGs calculated within each batch, the union of which were used to estimate the first 50 principal components across cells using the irbla R package (v2.3.3). Batch effects were removed across the first 30 PCs using the fastMNN57 implementation in the Bioconductor package batchelor (v1.6.2) (k=50). A k-nearest neighbour graph (k=20) was computed across these 30 batch-integrated PCs using the buildKNNGraph function implemented in the Bioconductor package scran (v1.18.3), which was then used to group cells into connected communities using Louvain58 clustering implemented in the R package igraph (v1.2.6). Clusters that displayed mixed profiles of T and other lymphoid lineages, i.e. CD19, CD20 and immunoglobulin genes, were classed as doublets and removed from down-stream analyses. Clusters indicative of NK cells (CD3-CD56+) were subsequently annotated as such and removed from T cell analyses. Remaining clusters were annotated using a combination of canonical protein & mRNA (italicised) markers for major αβ T cells (CD4, CD8, CCR7, CD45RA, CD45RO, CD62L, CD27, CD38, CD44, CXCR5, CD40LG CCR7, FOXP3, IKZF2), γδT cells (Vγ9, Vγ2, TRGV9, TRDV2) and invariant T cells; MAIT (Vα24-Jα18, TRAV1.2), NKT (CD3, CD16, CD56, NCAM1, NCR1, FCGR3A). Polarized CD4+ T cell annotations were refined using the combination of transcription factor genes and expressed cytokines for the respective helper T cell types: Th1 (IFNG, TBX21, TNFA), Th2 (GATA3, IL4, IL5), Th17 (RORC, IL17A, IL17F, IL21). Where clusters appeared heterogeneous in their expression of T cell lineage markers, single cell annotations were refined based on the co-expression of specific marker gene and protein pairs. Dot plots to visualise marker protein and mRNA expression across clusters were generated using the R package ggplot2 (v3.3.3). UMAP59 was used to project all single T cells into a 2D space (k=31) using the first 30 batch-integrated PCs as input using the R package umap. R version 4.0.3 and Bioconductor version 3.12 were used for all analyses.

T cell differential gene expression analysis
Differential gene expression (DGE) analysis was performed across COVID-19 disease severity groups, ordered from healthy > asymptomatic > mild > moderate > severe > critical. Donor pseudo-bulk samples were first created by aggregating gene counts for each annotated T cell type, within each donor, where there were at least 20 cells of that type. Genes with fewer than 3 counts in any given pseudo-bulk, or fewer than 5 counts in total across donor pseudo-bulk samples, were removed prior to analysis. DGE testing was performed using a negative binomial generalized linear model (NB GLM) implemented in the Bioconductor package edgeR60,61 (v3.32.1). Statistically significant DE genes were defined with FDR < 0.1. Functional annotation enrichment was performed using the Bioconductor package enrichR62(v3.0). Up- and down-regulated DE genes in each T cell type were used as input, testing separately against the MSigDB Hallmark 2020 and Transcription Factor Protein-Protein Interactions gene sets. Significant enrichments were defined with 1% FDR.

T cell receptor analysis
Single-cell TCRs were computed from the TCR-seq data using Cellranger v4.0.0. The unfiltered output of reconstructed TCR contigs across all 3 sites (Newcastle, Cambridge, London) were combined prior to filtering using: 1) full length CDR3, 2) droplet barcode matched a T cell droplet, 3) productive CDR3 spanning V+J genes. Chain-specific TCR clones were defined for each observed α and β chain by first concatenating the V, J and identical CDR3 nucleotide sequences. For each single T cell, these chains were then combined to form a single clonotype, removing cells that contained: 1) > 2 β chains and > 2 α chains, 2) a single α or a single β chain only. T cells with exactly 2 β chains and 1 α chain, or those with exactly 2 α chains and 1 β chain were retained. TCR clonotypes were counted within each donor sample, and expanded clones were defined where > 1 cell was assigned to the TCR clonotype.

The proportion of expanded clones as a function of a linear trend across disease severity groups was modelled using logistic regression, adjusted for age, gender and batch. A separate model was run for each T cell subtype which contained at least 5 cells assigned to the expanded TCR clonotypes. Linear trend p-values were corrected for multiple testing using the Benjamini & Hochberg procedure63.

The TE:EM ratio was calculated within each donor, using the number of observed expanded clonotypes. The TE:EM ratio change across COVID-19 severity was tested using a robust linear model implemented in the R package robustbase (v0.93-7), regressing TE:EM ratio on either disease severity as an ordered linear variable (asymptomatic > mild > moderate > severe > critical) or symptom duration, adjusted for age, gender and batch. Statistical significance was defined based on the linear trend across disease severity ($p \leq 0.01$). An equivalent analysis was performed, restricted to patients with a shorter symptom duration (≤24 days).

Differential correlation analysis

Changes in the correlations between PBMC cell types were computed using a differential correlation analysis, implemented in the R package DCARS64 (v0.3.5). Cell type proportions were computed by normalizing the counts of each cell type within each donor by the total number of cells captured for that donor sample. Donor samples were ranked according to their disease severity (healthy > asymptomatic > mild > moderate > severe > critical). Differential correlation analysis was then performed between CD4.Tfh vs all B cell types. Statistically significant differentially correlated cell types were defined with empirical p-value ≤ 0.1, estimated from 10,000 permutations.

BCR V(D)J analysis
Single-cell V(D)J data from the 5’ Chromium 10x kit were initially processed with cellranger-vdj (4.0.0). BCR contigs contained in filtered_contigs.fasta and filtered_contig_annotations.csv from all three sites were then pre-processed using immcantation inspired preprocessing pipeline65 implemented in the dandelion python package; dandelion is a novel single cell BCR-seq analysis package for 10x Chromium 5' data. All steps outlined below are performed using dandelion v0.0.27.post2 and is available at https://github.com/clatworthylab/dandelion.

BCR preprocessing
Individual BCR contigs were re-annotated with igblastn v1.1.15 using the IMGT reference database (date downloaded: 30-June-2020)66 by calling changeo’s AssignGenes.py script and re-annotated contigs in blast format were parsed into the Adaptive Immune Receptor Repertoire (AIRR) standards 1.3 format with changeo’s MakeDB.py script. Amino acid sequence alignment information not present in the output from blast format were retrieved from re-annotation with igblastn in airr format. Heavy chain V-gene alleles were corrected for individual genotypes with TIgGER67 (v1.0.0) using a modified tigger-genotype.R script from immcantation suite. Germline sequences were reconstructed based on the genotype corrected V-gene assignments using changeo’s (v1.0.1) CreateGermines.py script; contigs which fail germline sequence reconstruction were removed from further analysis. Constant genes were re-annotated using blastn (v2.10.0+) with CH1 regions of constant gene sequences from IMGT followed by pairwise alignment against curated sequences to correct assignment errors due to insufficient length of constant regions.

BCR filtering
Contigs assigned to cells that passed quality control on the transcriptome data were retained for further quality control assessment, which includes checks for: i) contigs with mismatched locus, V-, J- and constant gene assignments were removed from the analysis; ii) cell barcodes with multiple heavy chain contigs were flagged for filtering. Exceptions to this would be when a) the multiple heavy chain contigs were assessed to have identical V(D)J sequences but assigned as different contigs belonging to the same cell by cellranger-vdj, b) when there is a clear dominance (assessed by difference in UMI count) by a particular contig, and c) if and when there is presence of one IgM and one IgD contig assigned to a single cell barcode. In the first two cases, the contig with the highest UMI count is retained; iii) cell barcodes with multiple light chain contigs were flagged for filtering; iv) in situations where cell barcodes are matched with only light chain contigs, the contigs would be dropped from the V(D)J data but transcriptome barcode will be retained.

B cell clone/clonotype definition
BCRs were grouped into clones/clonotypes based on the following sequential criterion that applies to both heavy chain and light chain contigs – i) identical V- and J- gene usage, ii) identical junctional CDR3 amino acid length, and iii) at least 85% amino acid sequence similarity at the CDR3 junction (based on hamming distance). Light chain pairing is performed using the same criterion within each heavy chain clone. Only samples collected at day 0 of the study were analyzed from this step onwards and clones/clonotypes were called across the entire dataset; the sample from one of the donors who was subsequently found to have a B cell malignancy was separated from the analysis and processed independently.

B cell clone/clonotype network
Single-cell BCR networks were constructed using adjacency matrices computed from pairwise Levenshtein distance of the full amino acid sequence alignment for BCR(s) contained in every pair of cells within each disease severity cohort. Construction of the Levenshtein distance matrices were performed separately for heavy chain and light chain contigs and the sum of the total edit distance across all layers/matrices was used as the final adjacency matrix. To construct the BCR neighborhood graph, a minimum spanning tree was constructed on the adjacency matrix for each clone/clonotype, creating a simple graph with edges indicating the shortest edit distance between a B cell and its nearest neighbor. Cells with identical BCRs i.e. cells with a total pairwise edit distance of zero are then connected to the graph to recover edges trimmed off during the minimum spanning tree construction step. Fruchterman-reingold graph layout was generated using a modified method to prevent singletons from flying out to infinity in networkx (v2.5). Visualisation of the resulting single-cell BCR network is achieved via transferring the graph to relevant anndata slots, allowing for access to plotting tools in scanpy.

The use of the BCR network properties for computing gini indices was inspired from bulk BCR-seq network analysis methods where distribution of clone sizes and vertex sizes (sum of identical BCR reads) in BCR clone networks were used to infer the relationships between BCR clonality, somatic hypermutation and diversity68. However, there are challenges with native implementation of this approach for single-cell data. Firstly, to enable calculation of network-based clone/cluster and vertex/node size distribution, BCR networks needed to be reduced such that nodes/cells with identical BCRs had to be merged and counted; this required the re-construction of BCR networks per sample and discarding single-cell level information. Furthermore, the process of node contraction and counting of merging events requires significant computation time and resource. Secondly, this approach is dependent on sufficient coverage of the BCR repertoire, as the BCRs from the number of cells sampled (post-QC) may not necessarily recapitulate the entire repertoire, which may under- or over-represent merged counts for gini index calculation. We propose the use of node closeness centrality computed on each expanded clone (clone size > 1) as an alternative metric to emulate the statistics to adapt to the single-cell nature of the data; closeness centrality defines how close and central each node is with respect to other nodes in the graph and therefore cells with identical BCRs will have high closeness centrality scores, due to the way the BCR network is constructed in dandelion. Thus, we can quickly calculate if cells across clones, and/or samples overall, in the entire graph display proportionately/disproportionately high or low closeness centrality scores. One caveat to the current implementation is that it is only meaningful if there are clonotypes with at least two cells as scores will only be computed for non-singleton components of the graph. Gini indices are computed using skbio.diversity.alpha.gini_index (scikit-bio v0.5.6) with the trapezoids method after clone definition and network generation. Summary visualisation was performed using plotting tools in seaborn (v0.11.0).

Definition of BCR convergence across patients
BCR overlap was determined by collapsing sharing incidence of V- and J- gene usage and CDR3 amino acid sequences, in both heavy and light chains, between individuals into a binarized format (1 or 0). The information is turned into an adjacency matrix where an edge is created between two individuals if there is at least one clonotype (at least 1 cell from each individual displays an identical combination of heavy and light chain V- and J- gene usage with allowance for somatic hypermutation at the CDR3 junctional region) that is similar between the two

individuals. Visualisation is achieved using the CircosPlot function from nxviz package (v0.6.2).

For manuscripts utilizing custom algorithms or software that are central to the research but not yet described in published literature, software must be made available to editors and reviewers. We strongly encourage code deposition in a community repository (e.g. GitHub). See the Nature Research guidelines for submitting code & software for further information.

Data

Policy information about availability of data

All manuscripts must include a data availability statement. This statement should provide the following information, where applicable:

- Accession codes, unique identifiers, or web links for publicly available datasets
- A list of figures that have associated raw data
- A description of any restrictions on data availability

The dataset from our study can be explored interactively through a web portal: https://covid19cellatlas.org. The data object, as a h5ad file, can also be downloaded from https://covid19cellatlas.org. The processed data is available to download from Array Express using accession number E-MTAB-10026.

Field-specific reporting

Please select the one below that is the best fit for your research. If you are not sure, read the appropriate sections before making your selection.

☒ Life sciences ☐ Behavioural & social sciences ☐ Ecological, evolutionary & environmental sciences

For a reference copy of the document with all sections, see nature.com/documents/nr-reporting-summary-flat.pdf

Life sciences study design

All studies must disclose on these points even when the disclosure is negative.

Sample size

The sample size was not predetermined and all samples that were available were processed. Data from samples across the three sites were pooled to increase sample size for statistical power.

Data exclusions

Doublets:
Any cells labeled as doublets (as defined below) were excluded from the analysis.
Newcastle:
Scrublet (v0.2.1) was applied to each sample to generate a doublet score. These formed a bimodal distribution so the tool's automatic threshold was applied.
Cambridge:
Non-empty droplets were called within each multiplexed pool of donors using the emptyDrops function implemented in the Bioconductor package DropletUtils (v1.10.3), using a UMI threshold of 100 and FDR of 1%. The probability of being a doublet was estimated for each cell per sample (that is one 10x lane) using the "doubletCells" function in scran based on highly variable genes (HVGs). Next, we used "cluster_walktrap" on the SNN-Graph that was computed on HVGs to form highly resolved clusters per sample. Per-sample clusters with either a median doublet score greater than the median + 2.5 x MAD or clusters containing more than the median + 2.5 MAD genotype doublets were tagged as doublets. This was followed by a second round of highly-resolved clustering across the whole data set, in which again cells belonging to clusters with a high proportion (> 60%) of cells previously labelled as doublets were also defined as doublets.
London:
For pooled donor CITE-seq samples, the donor ID of each cell was determined by genotype-based demultiplexing using souporcell version 251. Souporcell analyses were performed with 'skip_remap' enabled and a set of known donor genotypes given under the 'common_variants' parameter. The donor ID of each souporcell genotype cluster was annotated by comparing each souporcell genotype to the set of known genotypes. Droplets that contained more than one genotype according to souporcell were flagged as 'ground-truth' doublets for heterotypic doublet identification. Ground-truth doublets were used by DoubletFinder 2.0.352 to empirically determine an optimal 'pK' value for doublet detection. DoubletFinder analysis was performed on each sample separately using 10 principal components, a 'pN' value of 0.25, and the 'nExp' parameter estimated from the fraction of ground-truth doublets and the number of pooled donors.

Low quality cells:
Combined raw data from the three centres was filtered to remove those that expressed fewer than 200 genes and >10% mitochondrial reads.

Myeloid cells:
Due to compositional differences across sites, when analysing differential abundance of myeloid populations (Figure 2), only samples from Newcastle and London were included.

Replication

Similar results were obtained using orthogonal methodological approaches including experiments performed across three laboratories. Due to sample availability, we were unable to include technical replicates.

Randomization

Samples were not allocated into experimental groups.

Blinding

Blinding was not relevant as it is a single arm study.

Reporting for specific materials, systems and methods

We require information from authors about some types of materials, experimental systems and methods used in many studies. Here, indicate whether each material, system or method listed is relevant to your study. If you are not sure if a list item applies to your research, read the appropriate section before selecting a response.

Materials & experimental systems

n/a	Involved in the study
☐	☒ Antibodies
☒	☐ Eukaryotic cell lines
☒	☐ Palaeontology and archaeology
☒	☐ Animals and other organisms
☐	☒ Human research participants
☒	☐ Clinical data
☒	☐ Dual use research of concern

Methods

n/a	Involved in the study
☒	☐ ChIP-seq
☐	☒ Flow cytometry
☒	☐ MRI-based neuroimaging

Antibodies

Antibodies used

anti-CD107a-BB700 antibody (1:50, clone H4A3, BD Bioscience, 566558), anti-CD14-FITC (1:50, clone M5E2, BD Biosciences, 555397), anti-CD19-FITC (1:50, clone 4G7, BD Biosciences, 345776), anti-CD137-Pe-Dazzle594 (1:50, clone 4B4-1, Biolegend, 309826), anti-CCR7-PE-Cy7 (1:50, clone G043H7, Biolegend, 353226), anti-CD45RO-APC-H7 (1:50, clone UCHL1, BD Biosciences, 561137), anti-CD28-BV480 (1:50, clone CD28.2, BD Biosciences, 566110), anti-CD4-BV785 (1:100, clone SK3, Biolegend, 344642), anti-CD3-BUV395 (1:50, clone UCHT1, BD Biosciences, 563546), anti-CD8-BUV496 (1:100, clone RPA-T8, BD Biosciences, 564804), anti-CD25-BUV737 (1:100, clone 2A3, BD Biosciences, 612806) and viability dye Zombie Yellow (1:200, Biolegend, 423104) anti-IL10-PE (1:10, clone JES3-19F1, BD Biosciences, 559330), anti-IFN-APC (1:25, Miltenyi Biotec, 130-090-762), anti-TNF-AF700 (1:50, clone MAb11, Biolegend, 502928), anti-IL2-BV421 (1:100, clone 5344.111, BD Biosciences, 562914), anti-CD154-BV605 (1:50, clone 24-31, Biolegend, 310826). TotalSeq™-C Human Universal Cocktail, V1.0, Biolegend.

Validation

The following antibodies have been validated to use as a 1:10 dilution.
anti-IL10-PE (clone JES3-19F1, BD Biosciences, 559330)

The following antibodies have been validated to use as a 1:25 dilution.
anti-IFN-APC (1:25, Miltenyi Biotec, 130-090-762)

The following antibodies have been validated to use as a 1:50 dilution.
anti-CD107a-BB700 antibody (clone H4A3, BD Bioscience, 566558)
anti-CD14-FITC (clone M5E2, BD Biosciences, 555397)
anti-CD19-FITC (clone 4G7, BD Biosciences, 345776)
anti-CD137-Pe-Dazzle594 (clone 4B4-1, Biolegend, 309826)
anti-CCR7-PE-Cy7 (clone G043H7, Biolegend, 353226)
anti-CD45RO-APC-H7 (clone UCHL1, BD Biosciences, 561137)
anti-CD28-BV480 (clone CD28.2, BD Biosciences, 566110)
anti-CD3-BUV395 (clone UCHT1, BD Biosciences, 563546)
anti-TNF-AF700 (clone MAb11, Biolegend, 502928)
anti-CD154-BV605 (clone 24-31, Biolegend, 310826)

The following antibodies have been validated to use as a 1:100 dilution.
anti-CD4-BV785 (clone SK3, Biolegend, 344642)
anti-CD8-BUV496 (clone RPA-T8, BD Biosciences, 564804)
anti-CD25-BUV737 (clone 2A3, BD Biosciences, 612806)
anti-IL2-BV421 (1:100, clone 5344.111, BD Biosciences, 562914)

We validated the following antibody to use 1 vial per 1 million cells.
TotalSeq™-C Human Universal Cocktail, V1.0, Biolegend

Human research participants

Policy information about studies involving human research participants

Population characteristics

Population characteristics are outlined in Supplementary Table 2.
We collected samples from patients aged 21-86 and both male and female.
Other characteristics were not used as covariates in the study.

Recruitment

Newcastle:
Patients were recruited and consented under the Newcastle Biobank (REC 17/NE/0361, IRAS 233551) study and ethical governance.

Cambridge:
Study participants were recruited between 31/3/2020 and 20/7/2020 from patients attending Addenbrooke's Hospital with a suspected or nucleic acid amplification test (NAAT) confirmed diagnosis of COVID-19 (including point of care testing (Collier et al., 2020; Mlcochova et al., 2020)), patients admitted to Royal Papworth Hospital NHS Foundation Trust or Cambridge and Peterborough Foundation Trust with a confirmed diagnosis of COVID-19, together with Health Care Workers identified through staff screening as PCR positive for SARS-CoV-2 (Rivett et al., 2020). Controls were recruited among hospital staff

	attending Addenbrooke's serology screening programme, and selected to cover the whole age spectrum of COVID-19 positive study participants, across both genders. Only controls with negative serology results (45 out of 47) were subsequently included in the study. Recruitment of inpatients at Addenbrooke's Hospital and Health Care Workers was undertaken by the NIHR Cambridge Clinical Research Facility outreach team and the NIHR BioResource research nurse team. Ethical approval was obtained from the East of England – Cambridge Central Research Ethics Committee ("NIHR BioResource" REC ref 17/EE/0025, and "Genetic variation AND Altered Leukocyte Function in health and disease - GANDALF" REC ref 08/H0308/176). All participants provided informed consent.<br><br>London:<br>Subjects 18 years and older were recruited from two large hospital sites in London, United Kingdom, namely University College London Hospitals NHS Foundation Trust and Royal Free London NHS Foundation Trust during the height of the pandemic in the United Kingdom (April to July 2020) and gave informed consent.
Ethics oversight	Newcastle Biobank (REC 17/NE/0361, IRAS 233551)<br>IV-LPS: REC (17/YH/0021)<br>("NIHR BioResource" REC ref 17/EE/0025, and "Genetic variation AND Altered Leukocyte Function in health and disease - GANDALF" REC ref 08/H0308/176)<br>UCL Great Ormond Street Institute of Child Health (REC reference: 19/NW/0171, IRAS project ID 261511)

Note that full information on the approval of the study protocol must also be provided in the manuscript.

Flow Cytometry

Plots

Confirm that:

☒ The axis labels state the marker and fluorochrome used (e.g. CD4-FITC).

☒ The axis scales are clearly visible. Include numbers along axes only for bottom left plot of group (a 'group' is an analysis of identical markers).

☒ All plots are contour plots with outliers or pseudocolor plots.

☒ A numerical value for number of cells or percentage (with statistics) is provided.

Methodology

Sample preparation	PBMCs were isolated from blood samples using Lymphoprep (StemCell Technologies) density gradient centrifugation as per manufacturer's instructions. Single cell suspensions were then washed with Dulbecco's phosphate buffered saline (PBS) (Sigma) and frozen in 5-10 million cell aliquots in 90% (v/v) heat inactivated fetal calf serum (FCS) (Gibco) 10% (v/v) DMSO (Sigma Aldrich). Purified PMBC were thawed at 37°C, transferred into a 15 mL tube with 10 mL pre-warmed complete culture media RPMI-1640 medium (Sigma Aldrich, R0883) supplemented with 10% (v/v) FCS (Gibco, 10270-106), 1% (v/v) Penicillin/ Streptomycin (100 U/mL and 100 µg/mL respectively; Sigma Aldrich, P0781) and 1% (v/v) L-Glutamine (2 mM; Sigma Aldrich, G7513), referred as RPMI10, followed by centrifugation at 500 g for 5 min. Cell pellet was resuspended in 500 µL RPMI10 with added DNAse (1 µg/mL, Merck, 10104159001), divided into 5 wells of round bottom 96-well plate and left to rest at 37° C for an hour. Cells were stimulated with SARS-CoV-2 PepTivator peptide S for pan-HLA (2 µg/mL, Miltenyi Biotech, 136-126-700) and PMA/Ionomycin as a control (2 µL/mL, Cell Activation cocktail, Biolegend, 423301), and incubated at 37°C for 2 h. Negative controls were left untreated. Brefeldin A (2 µg/mL, GolgiPlug, BD Bioscience, 555029) and anti-CD107a-BB700 antibody (1:50, clone H4A3, BD Bioscience, 566558) was added for additional 4 h into all conditions. Cells were stained for detection of activation induced markers and intracellular cytokines 6 h after stimulation and subjected to flow cytometry. PBMC stimulated for 6 h with the SARS-Cov-2 peptide were washed with PBS, and cell surface stained for 1 h at room temperature: anti-CD14-FITC (1:50, clone M5E2, BD Biosciences, 555397), anti-CD19-FITC (1:50, clone 4G7, BD Biosciences, 345776), anti-CD137-Pe-Dazzle594 (1:50, clone 4B4-1, Biolegend, 309826), anti-CCR7-PE-Cy7 (1:50, clone G043H7, Biolegend, 353226), anti-CD45RO-APC-H7 (1:50, clone UCHL1, BD Biosciences, 561137), anti-CD28-BV480 (1:50, clone CD28.2, BD Biosciences, 566110), anti-CD4-BV785 (1:100, clone SK3, Biolegend, 344642), anti-CD3-BUV395 (1:50, clone UCHT1, BD Biosciences, 563546), anti-CD8-BUV496 (1:100, clone RPA-T8, BD Biosciences, 564804), anti-CD25-BUV737 (1:100, clone 2A3, BD Biosciences, 612806) and viability dye Zombie Yellow (1:200, Biolegend, 423104). Cells were washed with PBS 2% (v/v) FCS, fixed with 4% (w/v) paraformaldehyde (ThermoFisher Scientific, 28908) and kept at 4°C overnight. Subsequently, cells were washed with PBS, permeabilized with Perm/Wash buffer (BD Biosciences, 554723) according manufacturer's instruction, and stained with intracellular antibodies for 1 h on ice: anti-IL10-PE (1:10, clone JES3-19F1, BD Biosciences, 559330), anti-IFN-APC (1:25, Miltenyi Biotec, 130-090-762), anti-TNF-AF700 (1:50, clone MAb11, Biolegend, 502928), anti-IL2-BV421 (1:100, clone 5344.111, BD Biosciences, 562914), anti-CD154-BV605 (1:50, clone 24-31, Biolegend, 310826). Cells were washed, transferred to flow cytometry 5 mL tubes.
Instrument	Symphony A5 flow cytometer (BD Biosciences)
Software	FlowJo V10 (BD Biosciences)
Cell population abundance	No sorting was performed
Gating strategy	Cells were gated using the following strategy. Live, single > Lineage, including CD3, CD4 and CD8 > Non "dump" including CD14 and CD19 > CD107a or CD137

☒ Tick this box to confirm that a figure exemplifying the gating strategy is provided in the Supplementary Information.
